# New Deoxyenhygrolides from *Plesiocystis pacifica* Provide Insights into Butenolide Core Biosynthesis

**DOI:** 10.3390/md20010072

**Published:** 2022-01-14

**Authors:** Joachim J. Hug, Louise Kjaerulff, Ronald Garcia, Rolf Müller

**Affiliations:** 1 Helmholtz-Institute for Pharmaceutical Research Saarland (HIPS), Helmholtz Centre for Infection Research (HZI), Saarland University, Campus E8 1, 66123 Saarbrücken, Germany; Joachim.Hug@helmholtz-hips.de (J.J.H.); Louisek@sund.ku.dk (L.K.); Ronald.Garcia@helmholtz-hips.de (R.G.); 2 German Center for Infection Research (DZIF), Partner Site Hannover-Braunschweig, 38124 Braunschweig, Germany; 3 Helmholtz International Labs, Department of Microbial Natural Products, Saarland University, Campus E8 1, 66123 Saarbrücken, Germany

**Keywords:** myxobacteria, *Plesiocystis pacifica*, enhygrolides, deoxyenhygrolides, natural products, butenolide, furanolide

## Abstract

Marine myxobacteria present a virtually unexploited reservoir for the discovery of natural products with diverse biological functions and novel chemical scaffolds. We report here the isolation and structure elucidation of eight new deoxyenhygrolides (**1**–**8**) from the marine myxobacterium *Plesiocystis pacifica* DSM 14875^T^. The herein described deoxyenhygrolides C–J (**1**–**8**) feature a butenolide core with an ethyl residue at C-3 of the γ-lactone in contrast to the previously described derivatives, deoxyenhygrolides A and B, which feature an isobutyl residue at this position. The butenolide core is 2,4-substituted with a benzyl (**1**, **2** and **7**), benzoyl (**3** and **4**) or benzyl alcohol (**5, 6** and **8**) moiety in the 2-position and a benzylidene (**1**–**6**) or benzylic hemiketal (**7** and **8**) in the 4-position. The description of these new deoxyenhygrolide derivatives, alongside genomic in silico investigation regarding putative biosynthetic genes, provides some new puzzle pieces on how this natural product class might be formed by marine myxobacteria.

## 1. Introduction

Natural products are an indispensable source for the development of new therapeutics for human and animal healthcare in the battle against various diseases [1]. Challenges such as the emergence of antimicrobial resistance, the increased difficulty in accessing novel bioactive chemical scaffolds and the exploitation of known terrestrial producers of natural products show the necessity to explore new reservoirs of producers [2]. The marine habitat represents an underexploited environment for natural product discovery [3,4], which in its ecosystems accommodates numerous marine organisms, invertebrates, fishes and plants. Endosymbiotic marine bacteria—which have been often revealed as the “true” producers of sponge-derived natural products—show, alongside other marine microorganisms, huge potential for the discovery of new and chemically diverse marine drugs [5]. Besides numerous species from *Actinobacteria* and *Cyanobacteria*, members of the phylum *Proteobacteria* from the ocean such as myxobacteria are known to produce complex natural products. Marine myxobacteria can be considered an almost untapped reservoir of marine drugs with equal potential to produce bioactive natural products featuring unique structures as terrestrial myxobacteria [6]. Currently, most of the halotolerant and obligate marine myxobacteria can be classified into the four genera *Enhygromyxa, Haliangium*, *Plesiocystis* and *Pseudenhygromyxa*.

While different natural products from the genera of *Enhygromyxa* and *Haliangium* have been isolated [7], no report of an isolated secondary metabolite from the genus *Pseudenhygromyxa* has been described [8]. Two strains belonging to the genus of *Plesiocystis*, namely, the type strain *Plesiocystis pacifica* DSM 14875^T^ (SIR-1^T^) and *Plesiocystis* sp. SHI-1 (DSM 14876), have been described as producing dehydrogenated menaquinone together with different fatty acids, such as the long-chain polyunsaturated fatty acid eicosatetraenoic acid (C20:4) [8]. In addition, in silico biosynthetic analysis on the draft genome of *P. pacifica* DSM 14875^T^ combined with metabolome analysis revealed numerous biosynthetic gene clusters (BGCs) accounting for the prospective production of natural products [9,10,11]. Hence, the genus *Plesiocystis* presents promising potential for the discovery of novel marine natural products. Examples of marine-derived myxobacterial natural products with potent biological activity are the cytotoxic miuraenamides from “*Paraliomyxa miuraensis”* SMH-27-4 [12,13] and the antifungal haliangicin [14,15].

**Figure 1:**
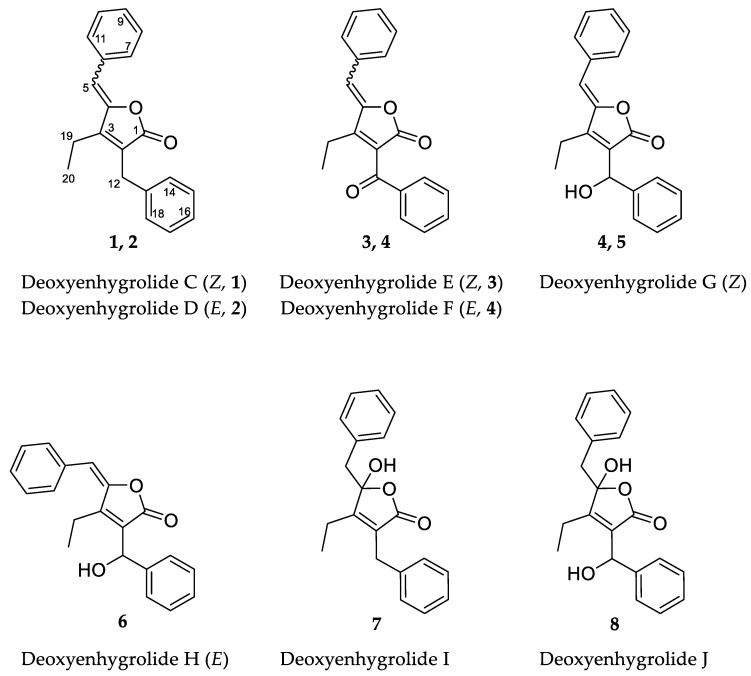
Chemical structures and carbon numbering of deoxyenhygrolides C–J (**1**–**8**).

In the course of our ongoing metabolome-guided screening and chemical profiling of marine myxobacterial strains, the analysis of the crude extract of *P. pacifica* DSM 14875^T^ revealed numerous unassigned secondary metabolites. We report here the isolation and structure elucidation of eight new deoxyenhygrolides (**1–8**) (Figure 1) [16,17] from this marine myxobacterium and provide a proposed biosynthetic pathway leading to the formation of deoxyenhygrolides.

## 2. Results and Discussion

### 2.1. Isolation and Structure Elucidation of ***1***–***8***

**Figure 2:**
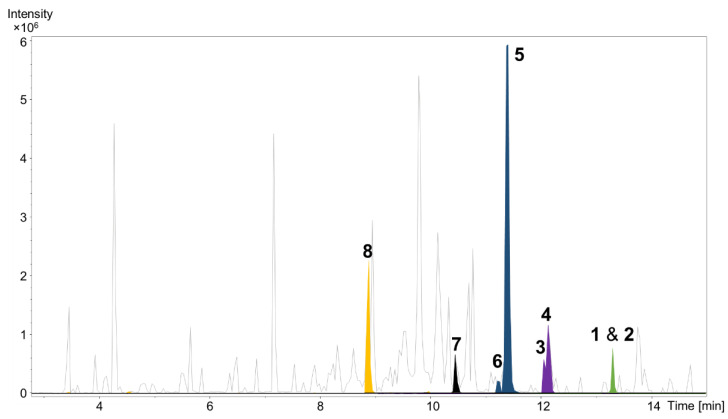
High performance liquid chromatography–mass spectrometry base peak chromatogram (HPLC–MS BPC) (grey) and extracted ion chromatograms (EICs) of **1** and **2** ([M + H]^+^ 291.1378 *m/z*, green), **3** and **4** ([M + H]^+^ 305.1174 *m/z*, purple), **5** and **6** ([M + H]^+^ 307.1331 *m/z*, blue), **7** ([M + H]^+^ 309.1487 *m/z*, black) and **8** ([M + H]^+^ 325.1436 *m/z*, orange) from *Plesiocystis pacifica* DSM 14875^T^ ethyl acetate extract.

Cultivation of *P. pacifica* DSM 14875^T^ was performed in RG224 medium with supplementation of adsorber resin XAD-16. The cell pellet and resin were used to perform liquid–liquid extraction to concentrate the deoxyenhygrolides in the ethyl acetate (EtOAc) phase for further analysis of the secondary metabolome via liquid chromatography (LC) coupled with high-resolution mass spectrometry (HRMS). The secondary metabolome of *P. pacifica* DSM 14875^T^ revealed—according to our in-house LC–MS metabolome database termed Myxobase [18]—eight previously uncharacterized secondary metabolites (Figure 2).

A cultivation volume of 13.2 L containing bacterial cells and adsorber resin XAD-16 was extracted with acetone followed by liquid–liquid extraction to yield a semi-crude EtOAc extract. The extract was separated on a Sephadex column and the fractions containing compounds **1–8** were further purified by semi-preparative HPLC.

High-resolution electrospray ionization mass spectrometry (HRESIMS) and isotopic pattern analysis of the molecular ions [M + H]^+^ *m/z* 291.1378 (calcd. for C_20_H_19_O_2,_ 291.1380, Δ = 0.6 ppm) and [M + Na]^+^ *m/z* 313.1207 (calcd. for C_20_H_18_O_2_Na, 313.1200, Δ = 2.2 ppm) indicated a molecular formula of C_20_H_18_O_2_ for **1** and **2**. Compounds **1** and **2** were isolated as a mixture of two inseparable *Z/E* isomers in a 3:2 ratio (from NMR integration). The NMR data of **1–2** resemble that of the previously isolated deoxyenhygrolides A and B from “*Enhygromyxa* *niigataensis”* SNB-1 [17]. The almost identical mass spectral data and high degree of similarity in the 1D and 2D NMR data of compounds **1** and **2** clearly connects these compounds as stereoisomers.

The ^1^H NMR spectrum of **1** contained resonances for protons that were attributed to two benzene rings (H-7/11, *δ*_H_ 7.78; H-8/10, *δ*_H_ 7.38; H-9, *δ*_H_ 7.31; H-14/18, *δ*_H_ 7.27; H-15/17, *δ*_H_ 7.29; H-16, *δ*_H_ 7.21), two methylenes (H-12, *δ*_H_ 3.74; H-19, *δ*_H_ 2.58), one methyl (H-20, *δ*_H_ 1.15) and one methine (H-5, *δ*_H_ 6.02). The ^13^C NMR spectrum of **1** showed signals for all 20 carbon atoms consisting of 11 methine groups, 2 methylenes, 1 methyl carbon and 6 fully substituted sp^2^-hybridized carbon atoms. Two of the six fully substituted carbons were associated with the benzene rings (C-6, *δ*_C_ 133.3; C-13, *δ*_C_ 138.1), whereas the other four constituted the fully substituted α,β-unsaturated γ-lactone (C-1, *δ*_C_ 170.7; C-2, *δ*_C_ 126.1; C-3, *δ*_C_ 154.9; C-4, *δ*_C_ 148.1). Ten of the eleven methine groups accounted for the remaining benzene ring constituents (C7/11, *δ*_C_ 130.6; C-8/10, *δ*_C_ 128.9; C-9, *δ*_C_ 128.9; C-14/18, *δ*_C_ 128.6; C-15/17, *δ*_C_ 128.9; C-16, *δ*_C_ 126.8). The last sp^2^-hybridized methine (C5, *δ*_C_ 109.1) is bound to C-4 of the lactone ring as well as one of the phenyls and is shielded by the neighboring groups. One methylene group (C-12, *δ*_C_ 29.8) is benzyl and bound to C-2, the other remaining methylene constitutes an ethyl group together with the sp^3^-hybridized methyl (C-20, *δ*_C_ 30.7).

**Figure 3:**
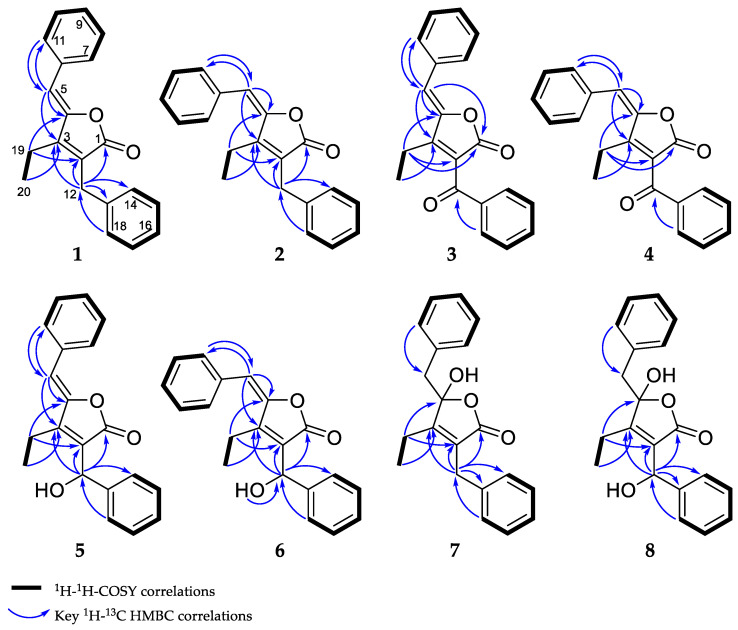
Observed correlations of **1**–**8**.

The combination of double-quantum filtered correlation spectroscopy (DQF–COSY), heteronuclear single quantum, coherence (HSQC) and heteronuclear multiple bond correlation (HMBC) experiments revealed the connectivity of the various segments within the skeletal structure of **1** and **2** (Figure 3). The partial structure of two monosubstituted phenyl moieties were revealed by HMBC correlations of **1** and **2**. Through the interpretation of further HMBC correlations, the connectivity of these aromatic substructures to five fully substituted sp^2^-hybridized carbons (C-2, C-3, C-4, C-6, C-13), one carbonyl (C-1), one methylene (C-12) and one sp^2^-hybridized methine (C-5) was determined. The remaining structural elements were assigned from HMBC correlations to give the butenolide moiety, which features an ethyl residue at position C-3 of the γ-lactone in contrast to the previously described derivatives, deoxyenhygrolides A and B with an isobutyl residue, at this position.

The assignment of the *Z* and *E* configuration of the C-4/5 double bond of **1** and **2** were done by comparison to previously identified signature resonance signals exhibited by the olefinic H-5, the carbon C-5 and the ethyl residues C-19 and C-20 [16,17]. The structure of **1** was assigned as the *Z*-configured isomer, since the resonance signal for the olefinic proton H-5 (**1**: *δ*_H_ 6.02, deoxyenhygrolide A: *δ*_H_ 5.70, enhygrolide A: *δ*_H_ 6.31) and C-5 (**1**: *δ*_C_ 109.1, deoxyenhygrolide A: *δ*_C_ 109.0, enhygrolide A: *δ*_C_ 111.0) were significantly shielded in comparison to the same positions in the *E*-configured isomers H-5 (**2**: *δ*_H_ 6.86, deoxyenhygrolide B: *δ*_H_ 6.58, enhygrolide B: *δ*_H_ 6.95) and C-5 (**2**: *δ*_C_ 114.7, deoxyenhygrolide B: *δ*_C_ 113.8, enhygrolide B: *δ*_C_ 116.4) [16,17].

**Figure 4:**
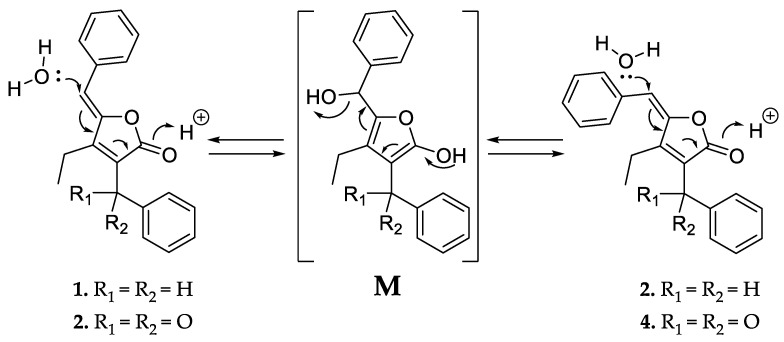
Proposed equilibrium of the tautomer pairs **1**/**2** and **3**/**4**, which includes a transient quinone methide-like intermediate (**M**).

Like **1** and **2**, compounds **3** and **4** were assigned as *Z*/*E* stereoisomers, which were found in a 2:3 ratio (from NMR integration). Compounds **3** and **4** were isolated as an inseparable mixture and isotopic pattern analysis of the molecular ions [M + H]^+^ *m/z* 305.1174 (calcd. for C_20_H_17_O_3,_ 305.1173, Δ = 0.3 ppm) and [2M + Na]^+^ *m/z* 631.2097 (calcd. for C_40_H_32_O_6_Na_,_ 631.2092, Δ = 0.7 ppm) indicated a molecular formula of C_20_H_16_O_3_ for **3** and **4**. The 1D and 2D NMR spectra obtained for **3** and **4** exhibited most of the structural features of **1** and **2**, except the missing methylene at C-12. The spectra of **3** and **4** instead feature a carbonyl resonance at *δ*_C_ 190.0/190.2 (**3**/**4**). The H-14/18 resonances were further deshielded, indicative of conjugation beyond the benzene ring, and the HMBC correlations from H-14/18 to *δ*_C_ 190.0/190.2 settle the position of the ketone at C-12, thus **3** and **4** are oxidized derivatives of **1** and **2**. The assignment is consistent with the earlier retention time of **3** and **4**, compared to **1** and **2**. Compound **3** could be assigned as the *Z*-configured derivative due to the observed shielded shifts at H-5 and C-5 in comparison to **4**, as well as a weak ROESY correlation between H-5 and H-19 in **3**; consequently, **4** was assigned as the *E*-configured stereoisomer. Since **1**/**2** and **3**/**4** could only be isolated as a mixture of *Z*/*E* stereoisomers, we cannot exclude the possibility that both **1**/**2** and **3**/**4** might be two pairs of tautomers, which are proceeded via a transient quinone methide-like intermediate (Figure 4).

Compounds **5** and **6** were isolated individually, since chromatographic separation and the abundance of **5** simplified the isolation and purification workflow, and no tautomerism was observed. The molecular formula was determined as C_20_H_18_O_3_ by HRESIMS (*m/z* [M + H]^+^ 307.1331 (calcd. for C_20_H_19_O_3,_ 307.1329, Δ = 0.7 ppm) and [M − H_2_O + H]^+^ 289.1226 (calcd. for C_20_H_17_O_2,_ 289.1224, Δ = 0.7 ppm)). The NMR spectra of **5** and **6** were particularly discriminated from those of **1**–**4** by the presence of an additional broad proton signal at *δ*_H_ 3.44/3.64 (**5**/**6**) without a HSQC correlation, as well as a methine signal at *δ*_H_ 5.78/5.69 (**5**/**6**) and *δ*_C_ 68.9 to which H-14/18 correlated in the HMBC spectra. Thus, **5** and **6** were assigned as the 12-hydroxylated derivatives of **1** and **2**, a single oxidation step towards the ketones of **3** and **4**. The most abundant isomer **5** showed strong ROESY correlations between H-5 ↔ H-19 ↔ H-12, which was in good agreement with the NMR data for the other isomers and pinpointed **5** as the *Z*-configured isomer. Thus, **5** was assigned as the deoxyenhygrolide derivative with a benzyl alcohol moiety in the 2-position with the systematic chemical name (*Z*)-5-benzylidene-4-ethyl-3-(hydroxy(phenyl)methyl)furan-2(5H)-one; consequently, **6** was assigned as the *E*-configured benzyl alcohol derivative. Compound **5** had a positive specific rotation with an [*α*]^20^_D_ value of +138° (CHCl_3_, *c* = 1.0) and **6** an [*α*]^20^_D_ value of 0° (CHCl_3_, *c* = 0.1). The absolute configuration of C-12 was not resolved, since an attempt to determine the absolute configuration of the hydroxyl group by Mosher ester derivatization, according to the procedure of Hoye et al., did not succeed [19].

Compound **7** has a molecular formula of C_20_H_20_O_3_, as determined by HRESIMS (*m/z* [M + H]^+^ 309.1487 (calcd. for C_20_H_21_O_3,_ 309.1486, Δ = 0.3 ppm) and [M − H_2_O + H]^+^ 291.1379 (calcd. for C_20_H_19_O_2,_ 291.1380, Δ = 0.3 ppm)). The acquired ^1^H NMR spectrum of compound **7** resembled the spectra of **1**–**6**; however, the presence of two broad proton resonances at *δ*_H_ 3.21 and 3.35 accounts for a new methylene functionality at C-5. In addition, the inspection of the recorded HMBC and HSQC spectra of **7** revealed significant different chemical shifts at C-4 (*δ*_C_ 109.1) and C-5 (*δ*_C_ 43.2), indicating that the C-4/5 double bond seen in the other isomers was missing. The chemical shift of the fully substituted C-4 suggested oxygenation at this position, which also fits well with the molecular formula suggesting addition of H_2_O in comparison to **1**/**2**; thus, **7** was assigned as their benzylic hemiketal derivative. Compound **7** featured a negative specific rotation with an [*α*]^20^_D_ value of –4° (CH_3_OH, *c* = 1.0).

HRESIMS and isotopic pattern analysis of the molecular ions [M + H]^+^ *m/z* 325.1436 (calcd. for C_20_H_21_O_4,_ 325.1435, Δ = 0.3 ppm) and [M − H_2_O + H]^+^ *m/z* 307.1331 (calcd. for C_20_H_21_O_4,_ 307.1329, Δ = 0.7 ppm) indicated the molecular formula of C_20_H_20_O_4_ for **8.** The acquired ^1^H NMR and ^13^C NMR spectra of compound **8** resembled the spectra of **7**, but the carbon resonance of the sp^3^-hybridized benzylidene carbon C-12 was deshielded to 68.2 ppm. Furthermore, the proton resonance of H-12 also shifted to 5.43 ppm, suggesting hydroxylation at C-12, as observed for **5** and **6**. The additional oxygen atom in the molecular formula relative to **7** combined with 2D NMR analysis (though broad resonances for H-5 and H-14/18 resulted in some HMBC correlations missing) supported **8** as the 12-hydroxylated derivative of **7**. Compound **8**, similar to **7**, featured a negative specific rotation with an [*α*]^20^_D_ value of –10° (CH_3_OH, *c* = 0.1).

DQF–COSY and key HMBC correlations for compounds **1**–**8** are shown in Figure 3 (see supporting information for NMR tables (Tables S1–S5) and spectra (Figures S28–S58)).

### 2.2. Bioactivity Testing

**Figure 5:**
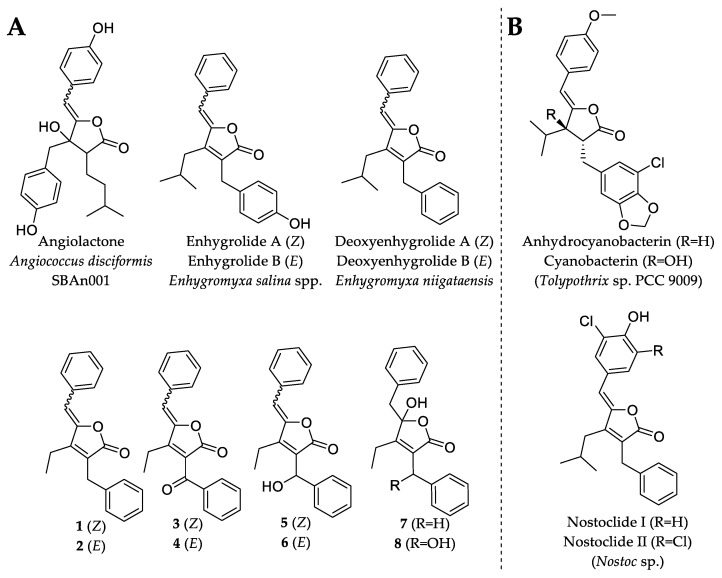
Chemical structures of natural products that feature the butenolide substructure. (**A**) Myxobacterial natural products with the butenolide substructure are angiolactone [22], enhygrolide A and B [16] and the herein described deoxyenhygrolides A–J. (**B**) Selected examples of natural products featuring the butenolide substructure are anhydrocyanobacterin, cyanobacterin [21] and nostoclide I and II, which originate from cyanobacteria [20].

Due to the diminutive production and availability of **1**, **2**, **3**, **4**, **6** and **7**, only the major compounds **5** and **8** were investigated for antimicrobial activity. Compound **5** exhibited weak antibacterial and antifungal activity against TolC-deficient *Escherichia coli* and *Mucor hiemalis* with minimal inhibitory concentrations (MIC) of 64 µg/mL. On the other hand, **5** showed no inhibitory activity against *E. coli* DSM 1116^T^, *Bacillus subtilis* DSM 10^T^, *Micrococcus luteus* DSM 1790 and *Wickerhamomyces anomalus* DSM 6766. The lack of any observed relevant microbial inhibitory activity in **5** or **8** was not completely unexpected, since deoxyenhygrolide A and B from *“Enhygromyxa niigataensis”* SNB-1 also exhibited no antimicrobial activity [17], in contrast to enhygrolide A (MIC value of 4 µg/mL against *Arthrobacter cristallopoietes*), which features a phenol group (Figure 5) [16]. Other natural products containing a butenolide core have been shown to exhibit biological activities, such as the cytotoxic nostoclides (IC_50_ = 10 µg/mL, neuro-2a CCL 131 and KB CCL17) [20] or the antibacterial cyanobacterin [21].

### 2.3. In Silico Investigation and Proposed Biosynthesis of ***1***–***8***

Although natural products that feature the butenolide scaffold are known from different microorganisms (Figure 5), the biosynthetic formation of these compounds has remained largely elusive. The genetic locus involved in the biosynthesis of cyanobacterins and nostoclides was identified by Gagunashivili et al. [23] during a genomic investigation of lichen-associated *Nostoc* strains. Due to the genetic homology between *Nostoc* spp. 210A/232 and *Tolypothrix* sp. PCC 9009 (the producer of cyanobacterins), the biosynthetic gene clusters (BGCs) of cyanobacterins and nostoclides have been annotated in silico; however, no proposal or hypothesis of the biosynthetic formation was provided. The cyanobacterin BGC from *Tolypothrix* sp. PCC 9009 was further characterized in a recent study in which the authors investigated the recombinant key enzymes involved in the biosynthetic formation of the butenolide (termed furanolide) core in the natural product cyanobacterin [23].

The cyanobacterin BGC comprises 11 genes termed *cybA–K*, of which the genes encoding CybC (long-chain acyl-CoA synthetase), CybE (thiamine pyrophosphate (TPP)-binding protein), CybF (3-oxoacyl-[acyl-carrier-protein]-synthase), and CybG (uncharacterized protein with NAD(P) binding site) seem to be of critical importance for the formation of the butenolide scaffold. In contrast to that, the enzymes encoded by the genes *cybA, B, D, H–J* are involved in amino acid (tyrosine) metabolism and tailoring reactions such as the flavin-dependent halogenase CybI, which seems to be specific for cyanobacterin biosynthesis.

In the publicly available draft genome of *P. pacifica* DSM 14875^T^, no homolog of the described *cybA–J* operon was found. However, two co-localized gene homologs of *cybE* and *cybF* were identified. Closely related homologs of these genes are also present in the genome of the enhygrolide A and B producers *Enhygromyxa salina* SWB005 and *Enhygromyxa salina* SWB007 [16] (see supporting information for amino acid sequence alignments (Figures S20–S25)).

**Figure 6:**
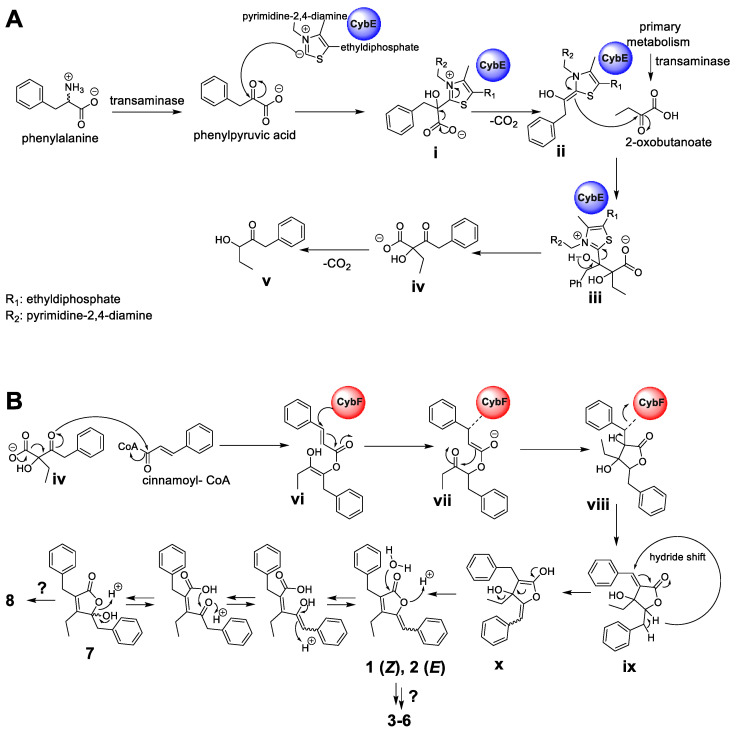
Putative biosynthetic formation of the deoxyenhygrolides C–J (**1**–**8**). (**A**) Formation of the acyloin intermediate **iv** by the thiamine pyrophosphate-(TPP)-dependent enzyme CybE homolog starting from the substrates phenylpyruvic acid and 2-oxobutanoate. (**B**) Proposed furanolide formation leading to **1**–**8** catalyzed by the identified CybF homolog from *P. pacifica* DSM 14875^T^. The proposed furanolide formation uses **iv** and cinnamoyl-CoA as substrate and performs a reaction cascade, which includes an acylation, C,C-bond formation and a hydride shift reaction. The proposed biosynthetic formation of **1**–**8** was adapted from Agostino et al. [24], which describes the underlying furanolide formation mechanism in the structurally related natural product cyanobacterin.

Hence, according to the retrobiosynthetic considerations of the core butenolide structure displayed by **1**–**8,** the formation would require three biosynthetic building blocks, namely, cinnamic acid (activated as cinnamoyl-coenzyme A (CoA)), 2-oxobutanoate and phenylpyruvic acid, and might involve the biosynthetic action of the CybE and CybF homologs identified in *P. pacifica* DSM 14875^T^. Similar to the proposed formation of the cyanobacterins [24], the biosynthesis of **1**–**8** might be initiated by CybE using the substrate phenylpyruvic acid and 2-oxobutanoate to perform an acyloin condensation-like reaction (Figure 6A). The substrates phenylpyruvic acid and 2-oxobutanoate are provided by transaminases from myxobacterial primary metabolism. Phenylpyruvic acid is proposed to be attached by the TPP-ylide cofactor via a nucleophilic attack to yield intermediate **i**. The decarboxylation of **i** leads to intermediate **ii**, which with its enamine, performs a nucleophilic attack at the ketone moiety of 2-oxobutanoate to deliver **iii.** The intermediate **iii** might then be released from the enzyme CybE to form the carboxylic acid **iv**. Similar reaction cascades have been previously described from different homologous enzymes [25,26]. In analogy to the hypothesis by Agostino et al. [24], the spontaneous decarboxylation of **iv** leads to the shunt product **v**. In contrast to cyanobacterin biosynthesis, cinnamoyl-CoA (instead of 4-coumaroyl-CoA) and the biosynthetic intermediate **iv** are further processed by the enzyme CybF to yield **vi** after *O*-acylation (Figure 6B). Intermediate **vi** undergoes a nucleophilic attack by the active-site residue of CybF, yielding the CybF-tethered enolate **vii**. *C*,*C*-bond formation of **vii** (via a nucleophilic attack of the enolate moiety) leads to **viii**, which is subsequently released from the biosynthetic enzyme CybF to yield **ix**. Despite significant efforts, we were unfortunately unable to genetically modify the producing strain and thus currently cannot provide direct evidence for the involvement of the respective genes in the hypothetical biosynthesis.

Since this furanolide biosynthetic formation resembles a Morita–Baylis–Hillman (MBH) reaction mechanism, Agostino et al. [24] identified CybF as the first MBH-catalyzing enzyme. The positions of the double bonds in the furanolide scaffold are not consistent with those observed in **1**–**8**. We therefore propose, similar to Agostino et al. [24], that a unique 1,4-hydride shift installs the required exocyclic double bond between C-5 and C-6 in **x**. In parallel, the proposed 1,4-hydride removes the double bond at the former coumaric acid residue between C-11 and C-12 (see intermediate **ix**). This hydride-shift does not only deliver enolate **x**, but might also be responsible for the occurrence of the benzylic hemiketal moiety in **7** and **8,** formed by the subsequent nucleophilic attack of water. Further modification by additional Red/Ox-enzyme might yield the structures of **3**–**6** and **8**.

The proposed formation might parallel the described biosynthesis of cyanobacterin (especially the formation of **1** and **2**) [24]; however, the herein presented structures of **3**–**8** would require additional tailoring steps. Whether the installation of the ketone functionality (displayed in **3** and **4**) or a hydroxyl group at the same position (like in **5**, **6** and **8**) is catalyzed by different enzymes remains elusive. A further deviation from the cyanobacterial pathway is the benzylic hemiketal moiety in **7** and **8**, which might reflect a biosynthetic intermediate during myxobacterial butenolide formation. In summary, the herein described natural products **1**–**8** and the biosynthetic insights provided by Agostino et al. [24] set the stage for further in-depth biochemical analysis of the butenolide/furanolide biosynthesis in microbial organisms.

## 3. Conclusions

We describe here the discovery of eight new deoxyenhygrolides from *P. pacifica* DSM 14875^T^ and further corroborate the recently investigated furanolide core biosynthesis. The putatively required tailoring reactions to obtain **1**–**8** set the stage for further in-depth biochemical analysis to elucidate the butenolide/furanolide biosynthesis in myxobacteria. In addition, **1**–**8** display the first group of natural products, which have been isolated and structurally elucidated from the marine myxobacterial genus *Plesiocystis*.

## 4. Materials and Methods

### 4.1. Maintenance of Myxobacterial Bacterial Cultures

*P. pacifica* DSM 14875^T^ was maintained in VY2-SWS agar (fresh Baker’s yeast 5 g/L, NaCl 20 g/L, Bacto Agar (Difco) 15 g/L [8], HEPES 2.38 g/L, pH adjusted 7.2 with NaOH). The commercial yeast was prepared by washing five times with 50 mL Milli-Q deionized water (MQ-H_2_O, obtained with Milli-Q Reference A + System^®^, Merck Millipore, Darmstadt, Germany). The sea water salt (SWS) solution was prepared according a previous study with a reduced iron(III) citrate concentration [8]. Liquid cultivations were prepared in RG244 medium (NaCl 20 g/L, ferric citrate 0.01 g/L, MgSO_4_·7H_2_O 8 g/L, CaCl_2_·2H_2_O 1 g/L, KCl 0.5 g/L, NaHCO_3_ 0.16 g/L, H_3_BO_3_ 0.02 g/L, KBr 0.08 g/L, SrCl_2_·6H_2_O 0.03 g/L, di-Na-β-glycerophosphate 0.01 g/L, 1 mL trace element solution (ZnSO_4_·7H_2_O 0.10 g/L, MnCl_2_·4H_2_O 0.03 g/L, H_3_BO_3_ 0.30 g/L, CoCl_2_·6H_2_O 0.20 g/L, CuCl_2_·2H_2_O 0.01 g/L, NiCl_2_·6H_2_O 0.02 g/L, NaMoO_4_·2H_2_O 0.03 g/L, EDTA 0.5 g/L, FeSO_4_·7H_2_O 0.2 g/L) skim milk 2 g/L, HEPES 2.38 g/L, pH adjusted to 7.2 with NaOH before autoclaving).

### 4.2. Standardized UHPLC–MS Conditions

UHPLC–HRMS analysis was performed on a Dionex UltiMate 3000 rapid separation liquid chromatography (RSLC) system (Thermo Fisher Scientific, Waltham, MA, USA) coupled to a Bruker maXis 4G ultra-high-resolution quadrupole time-of-flight (UHR-qTOF) mass spectrometer (MS) or a Bruker amaZon iontrap MS equipped with a high-resolution electrospray ionization (HRESI) source (Bruker Daltonics, Billerica, MA, USA). Separation of a 1 µL sample was achieved with a linear 5–95% gradient of acetonitrile with 0.1% formic acid in MQ–H_2_O with 0.1% formic acid on an ACQUITY BEH C_18_ column (100 × 2.1 mm, 1.7 µm d_p_) (Waters, Eschborn, Germany) equipped with a Waters VanGuard BEH C18 1.7 µm guard column at a flow rate of 0.6 mL/min and 45 °C over 18 min with UV detection by a diode array detector at 200–600 nm.

Mass spectrometry was acquired in centroid mode ranging from 150–2500 *m/z* at an acquisition rate of 2 Hz in positive MS mode. Source parameters were set to 500 V end plate offset; 4000 V capillary voltage; 1 bar nebulizer gas pressure; 5 L/min dry gas flow; and 200 °C dry gas temperature. Ion transfer and quadrupole parameters were set to 350 V_PP_ funnel RF; 400 V_PP_ multipole RF; 5 eV ion energy and 300 *m/z* low mass cut. Collision cell was set to 5.0 eV and pre-pulse storage set to 5 µs. Calibration was conducted automatically before every HPLC–MS run by injection of sodium formate and calibration on the respective clusters formed in the ESI source. All MS analyses were acquired in the presence of the lock masses C_12_H_19_F_12_N_3_O_6_P_3_, C_18_H_19_O_6_N_3_P_3_F_2_ and C_24_H_19_F_36_N_3_O_6_P_3_, which generated the [M + H]^+^ ions of 622.0289; 922.0098 and 1221.9906.

The HPLC–MS system was operated by HyStar 5.1 (Bruker Daltonics, Billerica, MA, USA) and LC chromatograms as well as UV spectra and mass spectrograms were analyzed with DataAnalysis 4.4 (Bruker Daltonics, Billerica, MA, USA). LC and MS conditions for the scheduled precursor list (SPL)-guided tandem MS data acquisitions were kept constant, according to section standardized UHPLC–MS conditions. Tandem MS data acquisition parameters were set to exclusively fragment SPL entries within a retention time tolerance of 0.2 min and a mass tolerance of 0.05 *m/z* for precursor ion selection. The method picked up to two precursors per cycle, applied smart exclusion after five spectra and performed CID and MS/MS spectra acquisition time ramping. CID energy was ramped from 35 eV for 500 *m/z* to 45 eV for 1000 *m/z* and 60 eV for 2000 *m/z*. The MS full scan acquisition rate was set to 2 Hz and MS/MS spectra acquisition rates were ramped from 1 to 4 Hz for precursor ion intensities of 10 kcts to 100 kcts.

### 4.3. Myxobacterial Fermentation and Extraction Procedure for LC–MS Analysis

*P. pacifica* DSM 14875^T^ was cultivated at 30 °C in 13.2L RG224 medium in Erlenmeyer flasks (six 5 L flasks each with a volume of 2 L medium and 200 mL pre-culture) for the isolation procedure of **1**–**8**. Each Erlenmeyer flask was inoculated with pre-culture inoculum (200 mL) in the same medium. After inoculation, the medium was supplemented with 2% (*v*/*v*) of sterile XAD-16 adsorber resin (Sigma-Aldrich Chemie GmbH, Taufkirchen, Germany) suspension in water to bind secondary metabolites in the culture medium. The cultures were shaken on a rotary shaker at 180 rpm for 14 days at 30 °C. Myxobacterial cells and adsorber resin were harvested together by centrifugation after fermentation (4 °C, 8000 rpm, 30 min), whereas the supernatant was discarded.

### 4.4. Isolation of ***1***–***8*** by Semi-Preparative HPLC

The cell pellet and XAD-16 resin were extracted with 4 × 400 mL acetone, then re-dissolved in 500 mL 90% MeOH and extracted with 130 mL hexane to remove fats and lipids. The polar fraction was concentrated in vacuo and partitioned between EtOAc (500 mL) and Milli-Q water (200 mL). The aqueous layer was then extracted twice with EtOAc (200 mL). The combined EtOAc extracts were dried in vacuo to yield 0.7 g crude extract. This crude extract was re-dissolved in 12 mL MeOH, centrifuged (3 min, 4000 rpm) and loaded on a Sephadex column (Ø = 2.5 cm, L = 135 cm), and eluted slowly with MeOH at 3 s per drop into fractions of 650 droplets.

Compound **7** and **8** was eluted in fractions 45–47 from the Sephadex column (71 mg) and was further purified by RP–HPLC (Luna II C18, Phenomenex, 250 × 10 mm, 5 µm, 100 Å) using H_2_O/MeCN with 0.1% FA added at 4 mL/min going from 15% to 60% MeCN in 12 min, then to 90% in 33 min. This yielded pure **8** (18.4 min, 3.2 mg) and **7** (22.1 min, 0.8 mg).

The remaining compounds (**1**–**6**) were eluted in fractions 52–56 from Sephadex (22 mg), which were similarly pooled and purified by RP-HPLC (Kinetex biphenyl, Phenomenex, 250 × 10 mm, 5 µm, 100 Å) using H_2_O/MeCN with 0.1% FA added at 4 mL/min going from 25% to 60% MeCN in 45 min, then 60% to 95% in 2 min. This yielded pure **6** (38.2 min, 0.5 mg), **5** (39.2 min, 11.6 mg), a mixture of **3** and **4** (45.3 min, 0.8 mg) and a mixture of **1** and **2** (49.8 min, 0.3 mg).

Deoxyenhygrolide C (**1**):

Colorless amorphous solid; UV λ_max_ 223 nm, 298 nm, HRESIMS *m/z* 291.1378 [M + H]^+^ (calcd. for C_20_H_19_O_2,_ 291.1380, Δ = 0.6 ppm), retention time 13.12 min.

Deoxyenhygrolide D (**2**):

Colorless amorphous solid; UV λ_max_ 223 nm, 330 nm, HRESIMS *m/z* 291.1378 [M + H]^+^ (calcd. for C_20_H_19_O_2,_ 291.1380, Δ = 0.6 ppm), retention time 13.27 min.

Deoxyenhygrolide E (**3**):

Colorless amorphous solid; UV λ_max_ 225 nm, 321 nm, HRESIMS *m/z* 305.1174 [M + H]^+^ (calcd. for C_20_H_17_O_3,_ 305.1173, Δ = 0.3 ppm), retention time 12.06 min.

Deoxyenhygrolide F (**4**):

Colorless amorphous solid; UV λ_max_ 224 nm, 352 nm, HRESIMS *m/z* 305.1174 [M + H]^+^ (calcd. for C_20_H_17_O_3,_ 305.1173, Δ = 0.3 ppm), retention time 12.14 min.

Deoxyenhygrolide G (**5**):

Colorless amorphous solid; UV λ_max_ 223 nm, 333 nm, HRESIMS *m/z* 307.1331 [M + H]^+^ (calcd. for C_20_H_19_O_3,_ 307.1329, Δ = 0.7 ppm), retention time 11.38 min.

Deoxyenhygrolide H (**6**):

Colorless amorphous solid; UV λ_max_ 222 nm, 303 nm, HRESIMS *m/z* 307.1331 [M + H]^+^ (calcd. for C_20_H_19_O_3,_ 307.1329, Δ = 0.7 ppm), retention time 11.22 min.

Deoxyenhygrolide I (**7**):

Colorless amorphous solid; UV λ_max_ 220 nm, HRESIMS *m/z* 309.1487 [M + H]^+^ (calcd. for C_20_H_21_O_3,_ 309.1486, Δ = 0.3 ppm), retention time 10.44 min.

Deoxyenhygrolide J (**8**):

Colorless amorphous solid; UV λ_max_ 213 nm, HRESIMS *m/z* 325.1436 [M + H]^+^ (calcd. for C_20_H_21_O_4,_ 325.1435, Δ = 0.3 ppm), retention time 8.83 min.

### 4.5. Structure Elucidation of **1**–**8** by NMR Spectroscopy

NMR spectra were acquired on a Bruker Ascend 700 and a Bruker Ultrashield 500 equipped with 5 mm cryoprobes using standard pulse sequences. All observed chemical shift values (*δ*) are given in in ppm and coupling constant values (*J*) in Hz. The signals of the residual solvent were used as internal reference (*δ*_H_ 3.31 and *δ*_C_ 49.0 for methanol-*d*_4_ and *δ*_H_ 7.26 and *δ*_C_ 77.16 for CDCl_3_). Standard pulse programs were used for the HMBC, HSQC and gCOSY experiments. The HMBC experiments were optimized for ^2,3^*J*_C-H_ = 6 Hz. To increase sensitivity, some measurements were conducted in 5 mm Shigemi tubes (Shigemi Inc., Allison Park, PA, USA). The NMR tables can be found in the supporting information. All structure formulae devised by NMR will be made publicly available under their corresponding name in NPatlas [27,28].

### 4.6. Chiroptical Measurements

Chiroptical measurements of **5**, **6**, **7** and **8** in CHCl_3_ (**5** and **6**) or MeOH (**7** and **8**) ([α]_D_) were obtained on a P-2000 polarimeter (JASCO, Easton, MD, USA) in a 10 mm QS quartz cuvette at 20 °C and at a wavelength of 589 nm.

### 4.7. Assessment of Antimicrobial Activities

All microorganisms used in this study were obtained from the German Collection of Microorganisms and Cell Cultures (DSMZ), the Coli Genetic Stock Center (CGSC), or were part of our internal collection, and were handled according to standard sterile microbiological procedures and techniques.

The major compounds **5** and **8** were tested in microbroth dilution assays on the following microorganisms: *E. coli* DSM 1116^T^, *E. coli tolC-deficient* efflux pump deletion mutant, *B. subtilis* DSM 10^T^, *M. luteus* DSM 1790, *W. anomalus* DSM 6766 and *M. hiemalis* DSM 2656. For microbroth dilution assays, the respective overnight cultures were prepared from cryogenically preserved cultures and were diluted to achieve a final inoculum of 10^4^–10^5^ colony-forming units (cfu)/mL. Serial dilutions of **5** and **8** in the respective growth medium (0.06 to 64 μg/mL) were prepared in sterile 96-well plates and the suspensions of bacteria or fungi were added. The cell suspension was added and microorganisms were grown for 24 h at either 30 °C or 37 °C. Minimum inhibitory concentrations (MIC) are defined as the lowest compound concentration where no visible growth is observed. **5** showed weak activity against *tolC*-deficient *E. coli* and *M. hiemalis*, with MICs of 64 µg/mL. The microbroth dilution assays were performed in triplicates.

### 4.8. Applied Software, DNA Sequence Analysis, and Bioinformatic Methods

The available genome sequences of the marine myxobacterial strains *P. pacifica* DSM 14875^T^ (GenBank accession number: ABCS00000000.1), *E. salina* SWB005 (GenBank accession number: PVNK00000000.1) and *E. salina* SWB007 (GenBank accession number: PVNL00000000.1) were screened for the presence of the cyanobacterin BGC *cybA–J* (GenBank accession number: ALWD01000000) originating from *Tolypothrix* *sp. PCC 9009* (also referred as *Scytonema hofmanni* UTEX B 2349) with the software Geneious Prime^®^ (Biomatters Ltd., Auckland, New Zealand, 2020.0.5) [29]. In order to find homologous genes or proteins, either the nucleotide or amino acid sequence of interest was aligned with the basic local alignment search tool (BLAST) against the in-house genome database or the publicly available nucleotide database. Sequence alignments were performed with the embedded Geneious alignment software with the following setups:

Pairwise alignments (alignment type: global alignment with free end gaps; cost matrix: Blosum62; gap open penalty: 12; gap extension penalty: 3.)

Multiple alignments (alignment type: global alignment with free end gaps; cost matrix: Blosum45; gap open penalty: 12; gap extension penalty: 3; refinement iterations: 2.)

The raw data from the alignments for in silico evaluation of the deoxyenhygrolide biosynthetic proteins were stored on the in-house server. The functional prediction of ORFs was performed by using protein blast and/or blastx programs and Pfam [30]. To obtain further information concerning the catalytic function of the identified biosynthetic proteins, the amino acid sequences were evaluated by the in silico protein homology analogy recognition engine 2 (Phyre2) [31].

## Data Availability

All data presented in this study are available from the corresponding author on reasonable request.

## Supplementary Materials

The following are available online at https://www.mdpi.com/article/10.3390/md20010072/s1. Figure S1. HPLC-MS BPC trace of purified **1** and **2** (top, blue) and UV/VIS (bottom, red) trace of **1** and **2**. Mass spectrum was acquired from 150–2000 *m/z*, in positive mode; UV/VIS detection by a DAD at 200–600 nm, Figure S2. HPLC-MS BPC trace of purified **3** and **4** (top, blue) and UV/VIS (bottom, red) trace of **3** and **4**. Mass spectrum was acquired from 150–2000 *m*/*z*, in positive mode; UV/VIS detection by a DAD at 200–600 nm, Figure S3. HPLC-MS BPC trace of purified **5** (top, blue) and UV/VIS (bottom, red) trace of **5**. Mass spectrum was acquired from 150–2000 *m*/*z*, in positive mode; UV/VIS detection by a DAD at 200–600 nm, Figure S4. HPLC-MS BPC trace of purified **6** (top, blue) and UV/VIS (bottom, red) trace of **6**. Mass spectrum was acquired from 150–2000 *m*/*z*, in positive mode; UV/VIS detection by a DAD at 200–600 nm, Figure S5. HPLC-MS BPC trace of purified **7** (top, blue) and UV/VIS (bottom, red) spectrum of **7**. Mass spectrum was acquired from 150–2000 *m*/*z*, in positive mode; UV/VIS detection by a DAD at 200–600 nm, Figure S6. HPLC-MS BPC trace of purified **8** (top, blue) and UV/VIS (bottom, red) spectrum of **8**. Mass spectrum was acquired from 150–2000 *m*/*z*, in positive mode; UV/VIS detection by a DAD at 200–600 nm, Figure S7. UV/VIS and partial ESI+MS spectra of purified **1** (291.1378 [M+H]^+^, 308.1647 [M+NH_4_]^+^, 313.1207 [M+Na]^+^, 603.2508 [2M+Na]^+^). RT: 13.05–13.15 min, Figure S8. UV/VIS and partial ESI+MS spectra of purified **2**. 291.1378 [M+H]^+^, 308.1648 [M+NH_4_]^+^, 313.1207 [M+Na]^+^, 603.2510 [2M+Na]^+^). RT: 13.20–13.30 min, Figure S9. UV/VIS and partial ESI+MS spectra of purified **3**. 305.1174 [M+H]^+^, 631.2097 [2M+Na]^+^. RT: 12.00–12.10 min, Figure S10. UV/VIS and partial ESI+MS spectra of purified **4**. 305.1174 [M+H]^+^, 631.2097 [2M+Na]^+^. RT: 12.10–12.20 min, Figure S11. UV/VIS and partial ESI+MS spectra of purified **5**. 289.1243 [M-H_2_O+H]^+^, 307.1331 [M+H]^+^, 635.2405 [2M+Na]^+^. RT: 11.30–11.45 min, Figure S12. UV/VIS and partial ESI+MS spectra of purified **6**. 289.1226 [M-H_2_O+H]^+^, 307.1331 [M+H]^+^, 635.2405 [2M+Na]^+^. RT: 11.15–11.25 min, Figure S13. UV/VIS and partial ESI+MS spectra of purified **7**. 291.1379 [M-H_2_O+H]^+^, 309.1487 [M+H]^+^, 326.1751 [M+NH_4_]^+^. RT: 10.38–10.48 min, Figure S14. UV/VIS and partial ESI+MS spectra of purified **8**. 289.12332 [M-2H_2_O+H]^+^, 261.1274 [M-2H_2_O-C_2_H_4_+H]^+^, 307.1331 [M-H_2_O+H]^+^, 279.1392 [M-H_2_O-C_2_H_4_+H]^+^, 325.1436 [M+H]^+^, 347.1255 [M+Na]^+^, 631.2710 [2M-H_2_O+H]^+^. RT: 8.80–8.90 min, Figure S15. Structure and carbon numbering of deoxyenhygrolide C and D (**1** and **2**), Figure S16. Structure and carbon numbering of deoxyenhygrolide E and F (**3** and **4**), Figure S17. Structure and carbon numbering of deoxyenhygrolide G and H (**5** and **6**), Figure S18. Structure and carbon numbering of deoxyenhygrolide I and J (**7** and **8**), Figure S19. General structure and carbon numbering of deoxyenhygrolide A–H (**1–8**). (**1**) R1: –H, R2: –H, (*Z*); (**2**) R1: –H, R2: –H, (*E*); (**3**) R1: –H, R2: =O, (*Z*); (**4**) R1: –H, R2: =O, (*E*); (**5**) R1: –H, R2: –OH, (*E*); (**6**) R1: –H, R2: –OH, (*Z*); (**7**) R1: –OH, R2: –H; (**8**) R1: –OH, R2: –OH, Figure S20. Amino acid alignment of myxobacterial CybE homologs from *P. pacifica* DSM 14875^T^ (CybE_Pacifica), *E. salina* SWB005 (CybE_SWB005) and *E. salina* SWB007 (CybE_SWB007). Pairwise identity: 45.0%, pairwise positive (BLSM62): 61.2%, Figure S21. Amino acid alignment of myxobacterial CybE homologs from *E. salina* SWB005 (CybE_SWB005) and *E. salina* SWB007 (CybE_SWB007). Pairwise identity: 57.3%, pairwise positive (BLSM62): 72.3%, Figure S22. Amino acid alignment of myxobacterial CybE homolog from from *P. pacifica* DSM 14875^T^ (CybE_Pacifica) and CybE from *Tolypothrix* *sp.* PCC 9009 (CybE_Tol9009). Pairwise identity: 44.0%, pairwise positive (BLSM62): 61.6%, Figure S23. Amino acid alignment of myxobacterial CybF homologs from *P. pacifica* DSM 14875^T^ (CybF_Pacifica), *E. salina* SWB005 (CybF_SWB005) and *E. salina* SWB007 (CybF_SWB007). Pairwise identity: 50.8%, pairwise positive (BLSM62): 66.5%, Figure S24. Amino acid alignment of myxobacterial CybF homologs from *E. salina* SWB005 (CybF_SWB005) and *E. salina* SWB007 (CybF_SWB007). Pairwise identity: 74.1%, pairwise positive (BLSM62): 86.5%, Figure S25. Amino acid alignment of myxobacterial CybF homolog from from *P. pacifica* DSM 14875^T^ (CybF_Pacifica) and CybF from *Tolypothrix* *sp.* PCC 9009 (CybE_Tol9009). Pairwise identity: 39.3%, pairwise positive (BLSM62): 59.6%, Figure S26. Phyre2 structure homology model of the thiamine pyrophosphate-binding protein (TPP) encoded by *cybE* from *Tolypothrix* *sp.* PCC 9009 (**A**) and the identified *cybE* homolog from *Plesiocystis pacifica* DSM 14875^T^ (models are based on the template c3ey9B; figure colored by rainbow *N* → *C* terminus), Figure S27. Phyre2 structure homology model of the furanolide encoded by *cybF* from *Tolypothrix* *sp.* PCC 9009 (**A**) and the identified *cybF* homolog from *Plesiocystis pacifica* DSM 14875^T^. (**A**) Model based on template c4x0oG. (**B**) Model based on template c6a9nA; figure colored by rainbow *N* → *C* terminus), Figure S28. ^1^H NMR spectrum of **1** and **2** (700 MHz, CDCl_3_), Figure S29. DQF-COSY spectrum of **1** and **2** (700 MHz, CDCl_3_), Figure S30. HSQC spectrum of **1** and **2** (700 MHz, CDCl_3_), Figure S31. HMBC spectrum of **1** and **2** (700 MHz, CDCl_3_), Figure S32. ^13^C NMR spectrum of **1** and **2** (700 MHz, CDCl_3_), Figure S33. ^1^H NMR spectrum of **3** and **4** (500 MHz, CDCl_3_), Figure S34. DQF-COSY spectrum of **3** and **4** (500 MHz, CDCl_3_), Figure S35. HSQC spectrum of **3** and **4** (500 MHz, CDCl_3_), Figure S36. HMBC spectrum of **3** and **4** (500 MHz, CDCl_3_), Figure S37. ^13^C NMR spectrum of **3** and **4** (125 MHz, CDCl_3_), Figure S38. ^1^H NMR spectrum of **5** (700 MHz, CDCl_3_), Figure S39. DQF-COSY spectrum of **5** (700 MHz, CDCl_3_), Figure S40. HSQC spectrum of **5** (700 MHz, CDCl_3_), Figure S41. HMBC spectrum of **5** (700 MHz, CDCl_3_), Figure S42. ROESY spectrum of **5** (700 MHz, CDCl_3_, 400 ms), Figure S43. ^13^C NMR spectrum of **5** (175 MHz, CDCl_3_), Figure S44. ^1^H NMR spectrum of **6** (700 MHz, CDCl_3_), Figure S45. DQF-COSY spectrum of **6** (700 MHz, CDCl_3_), Figure S46. HSQC spectrum of **6** (700 MHz, CDCl_3_), Figure S47. HMBC spectrum of **6** (700 MHz, CDCl_3_), Figure S48. ROESY spectrum of **6** (700 MHz, CDCl_3_, 300 ms), Figure S49. ^13^C NMR spectrum of **6** (175 MHz, CDCl_3_), Figure S50. ^1^H NMR spectrum of **7** (700 MHz, CD_3_OD), Figure S51. DQF-COSY spectrum of **7** (700 MHz, CD_3_OD), Figure S52. HSQC spectrum of **7** (700 MHz, CD_3_OD), Figure S53. HMBC spectrum of **7** (700 MHz, CD_3_OD), Figure S54. ^1^H NMR spectrum of **8** (500 MHz, CD_3_OD), Figure S55. DQF-COSY spectrum of **8** (500 MHz, CD_3_OD), Figure S56. HSQC spectrum of **8** (500 MHz, CD_3_OD), Figure S57. HMBC spectrum of **8** (500 MHz, CD_3_OD), Figure S58. ^13^C NMR spectrum of **8** (125 MHz, CD_3_OD), Table S1. Spectroscopic values of deoxyenhygrolide C and D (**1** and **2**) acquired in CDCl_3_ at 700 MHz, Table S2. Spectroscopic values of deoxyenhygrolide E and F (**3** and **4**) acquired in CDCl_3_ at 500 MHz, Table S3. Spectroscopic values of deoxyenhygrolide G and H (**5** and **6**) acquired in CDCl_3_ at 700 MHz, Table S4. Spectroscopic values of deoxyenhygrolide I and J (**7** and **8**) acquired in CD_3_OD at 700/500 MHz, respectively, Table S5. Comparison of ^13^C chemical shifts for **1–8**, Table S6. *cybE* and *cybF* homologs identified in different myxobacterial genome sequences, Table S7. Changed locus tag of *cybE* homolog.
