# Supplementary material for: New Deoxyenhygrolides from Plesiocystis pacifica Provide Insights into Butenolide Core Biosynthesis
Source: Mar Drugs. 2022 Jan 14;20(1):72. doi: 10.3390/md20010072 (PMC8777810; doi:10.3390/md20010072)
Supplement: Supplementary file 1 [file marinedrugs-20-00072-s001.zip › marinedrugs-1546166-supplementary.pdf]

marine drugs

# Supplementary Information

# New Deoxyenhygrolides from *Plesiocystis pacifica* Provide Insights into Butenolide Core Biosynthesis

**Joachim J. Hug [1,2,3†], Louise Kjaerulff [1,2†], Ronald Garcia[1,2,3] and Rolf Müller [1,2,3] ***

1 Helmholtz-Institute for Pharmaceutical Research Saarland (HIPS), Helmholtz Centre for Infection Research (HZI), Saarland University, Campus E8 1, 66123 Saarbrücken, Germany; Joachim.Hug@helmholtz-hips.de (J.J.H.), Louisek@sund.ku.dk (L.K.), Ronald.Garcia@helmholtz-hips.de (R.G.).

2 German Center for Infection Research (DZIF), Partner Site Hannover-Braunschweig, Germany

3 Helmholtz International Labs, Department of Microbial Natural Products, Saarland University, Campus E8 1, 66123 Saarbrücken, Germany

* Correspondence: rolf.mueller@helmholtz-hips.de

† These authors contributed equally to this work.

# Table of contents

1. MS spectra ... 3

1.1 Preparative LC-MS chromatograms ... 3

1.2 Partial ESI-MS spectra ... 6

2. NMR spectroscopic data for 1–8 ... 10

3. Genetic and biosynthetic investigations ... 15

3.1 Identification of *cybE* and *cybF* homologs in different myxobacteria ... 15

3.2 Sequence alignments and Phyre2 structures ... 17

4. $^{1}H$ and $^{13}C$ NMR spectra for 1–8 ... 20

4.1 NMR spectra of 1 and 2 ... 20

4.2 NMR spectra of 3 and 4 ... 26

4.3 NMR spectra of 5 ... 32

4.4 NMR spectra of 6 ... 39

4.5 NMR spectra of 7 ... 46

4.6 NMR spectra of 8 ... 51

5. References ... 57

# 1. MS spectra

## 1.1 Preparative LC-MS chromatograms

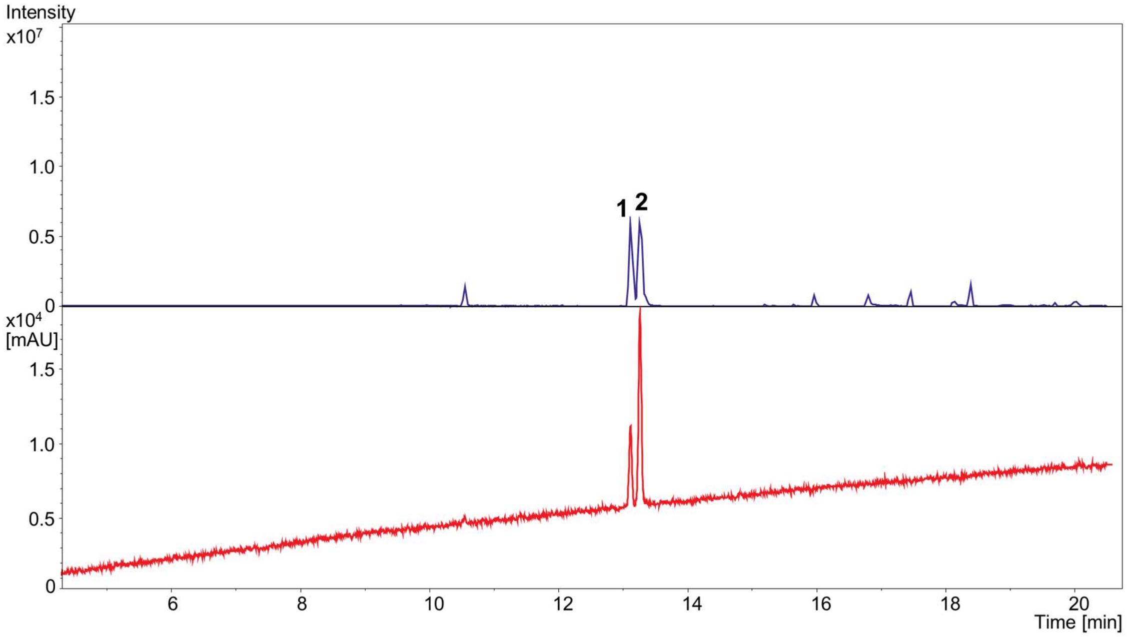


**Figure S1.** HPLC-MS BPC trace of purified **1** and **2** (top, blue) and UV/VIS (bottom, red) trace of **1** and **2**. Mass spectrum was acquired from 150–2000 *m/z*, in positive mode; UV/VIS detection by a DAD at 200–600 nm.

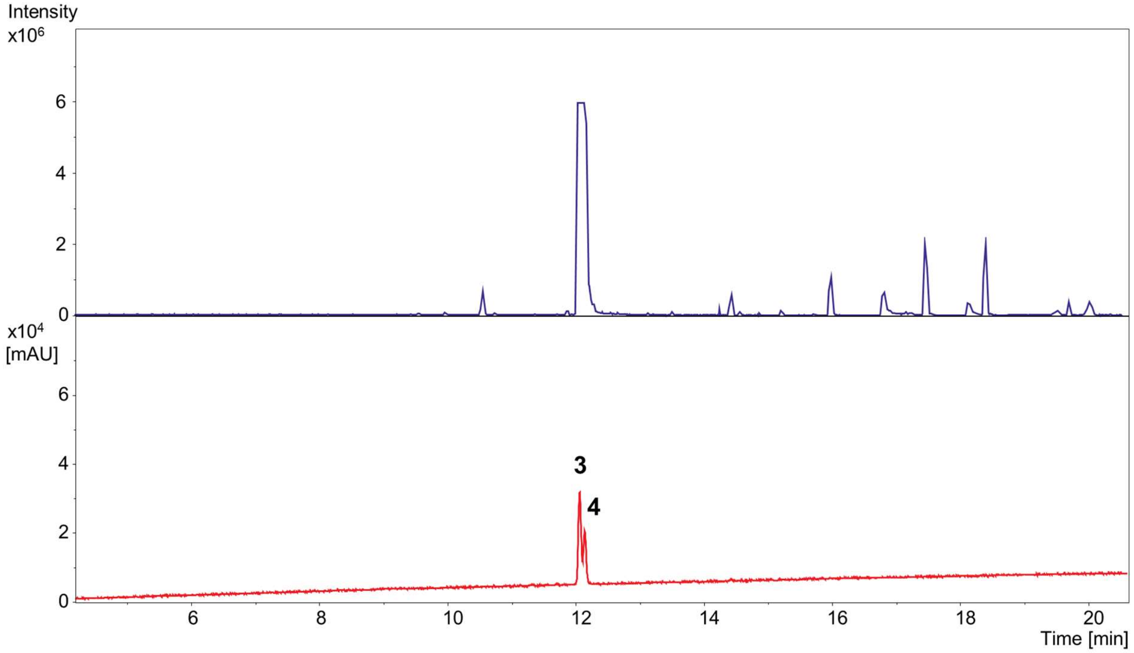


**Figure S2.** HPLC-MS BPC trace of purified **3** and **4** (top, blue) and UV/VIS (bottom, red) trace of **3** and **4**. Mass spectrum was acquired from 150–2000 *m/z*, in positive mode; UV/VIS detection by a DAD at 200–600 nm.

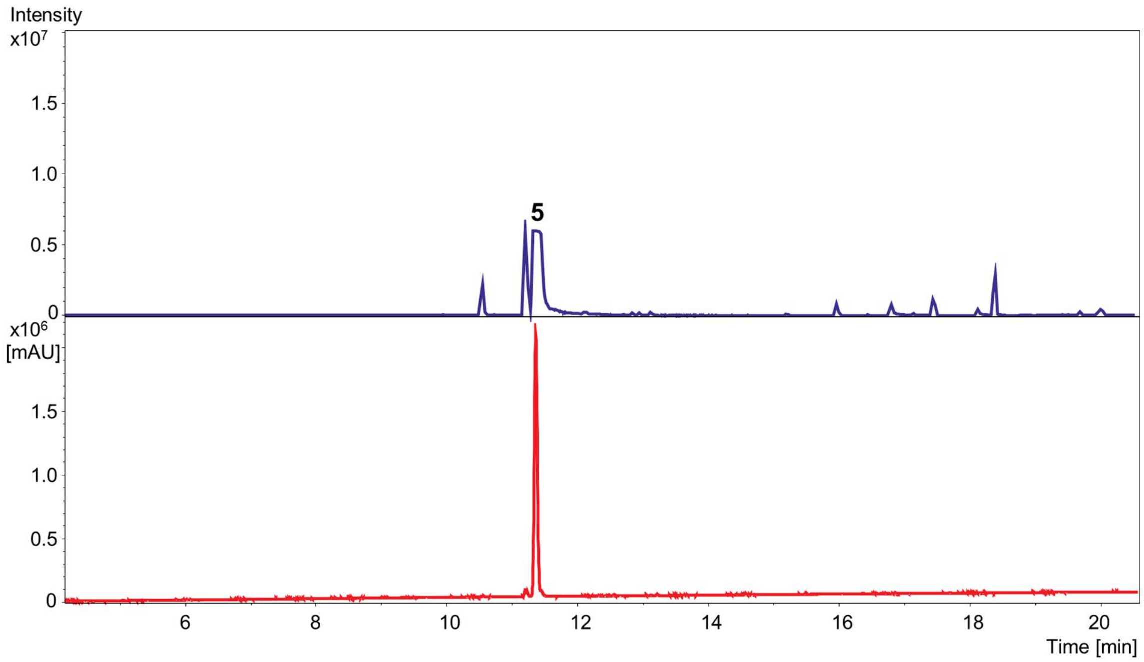


**Figure S3.** HPLC-MS BPC trace of purified **5** (top, blue) and UV/VIS (bottom, red) trace of **5**. Mass spectrum was acquired from 150–2000 *m*/*z*, in positive mode; UV/VIS detection by a DAD at 200–600 nm.

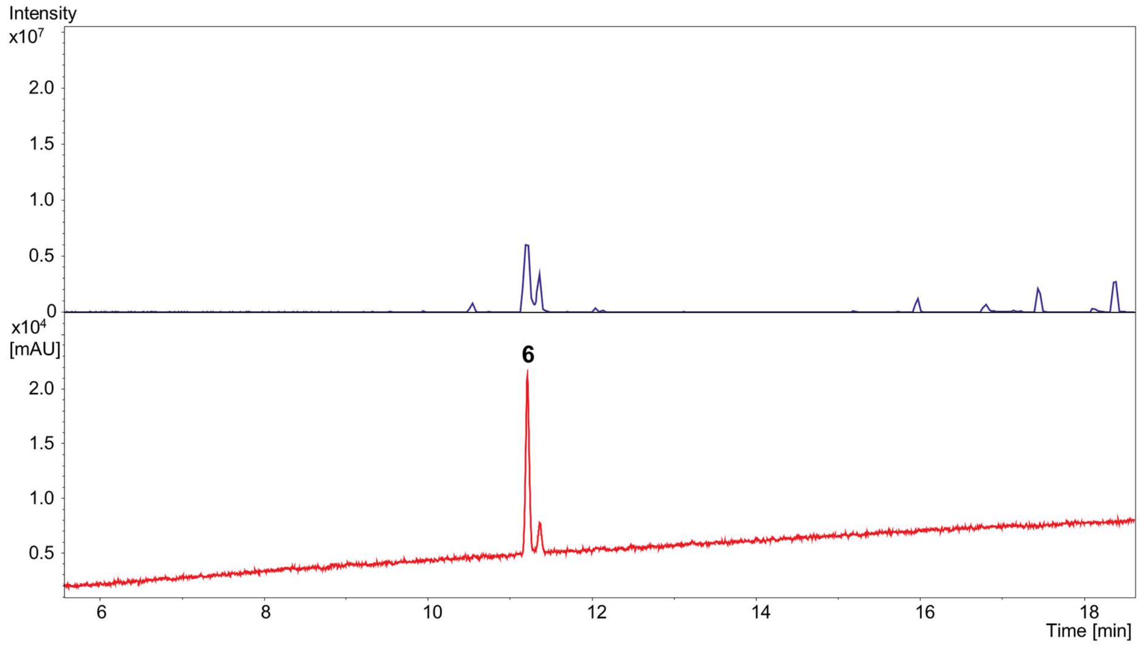


**Figure S4.** HPLC-MS BPC trace of purified **6** (top, blue) and UV/VIS (bottom, red) trace of **6**. Mass spectrum was acquired from 150–2000 *m*/*z*, in positive mode; UV/VIS detection by a DAD at 200–600 nm.

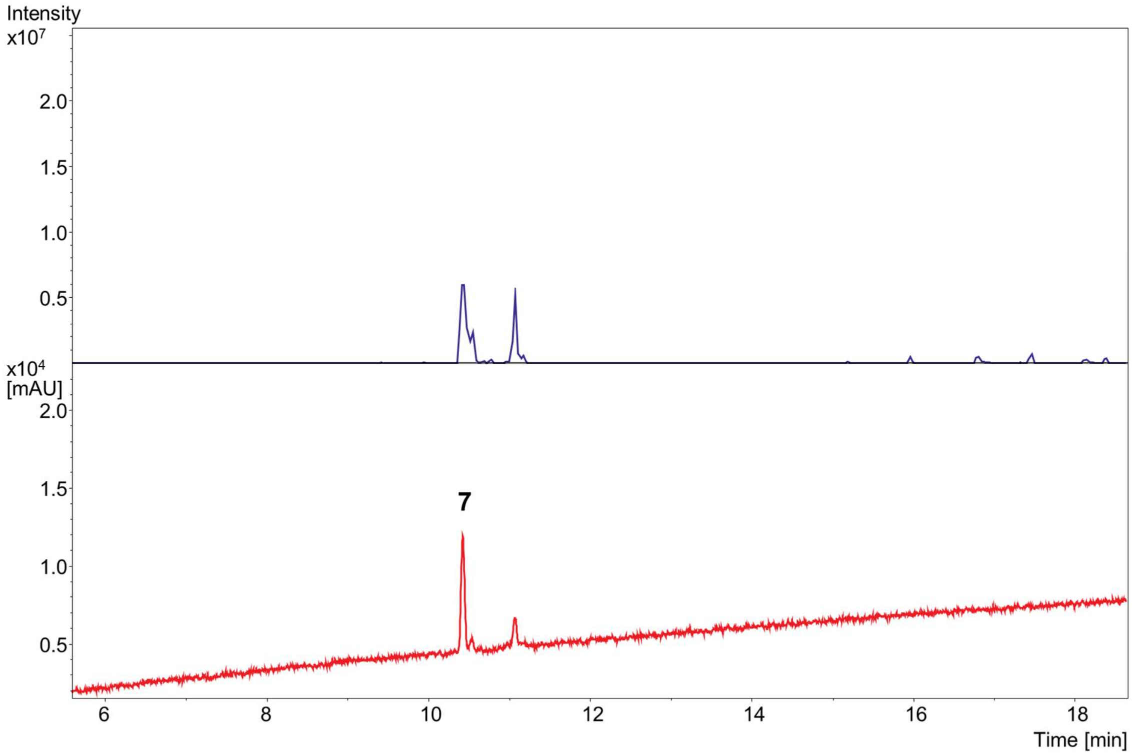


**Figure S5.** HPLC-MS BPC trace of purified **7** (top, blue) and UV/VIS (bottom, red) spectrum of **7**. Mass spectrum was acquired from 150–2000 *m*/*z*, in positive mode; UV/VIS detection by a DAD at 200–600 nm.

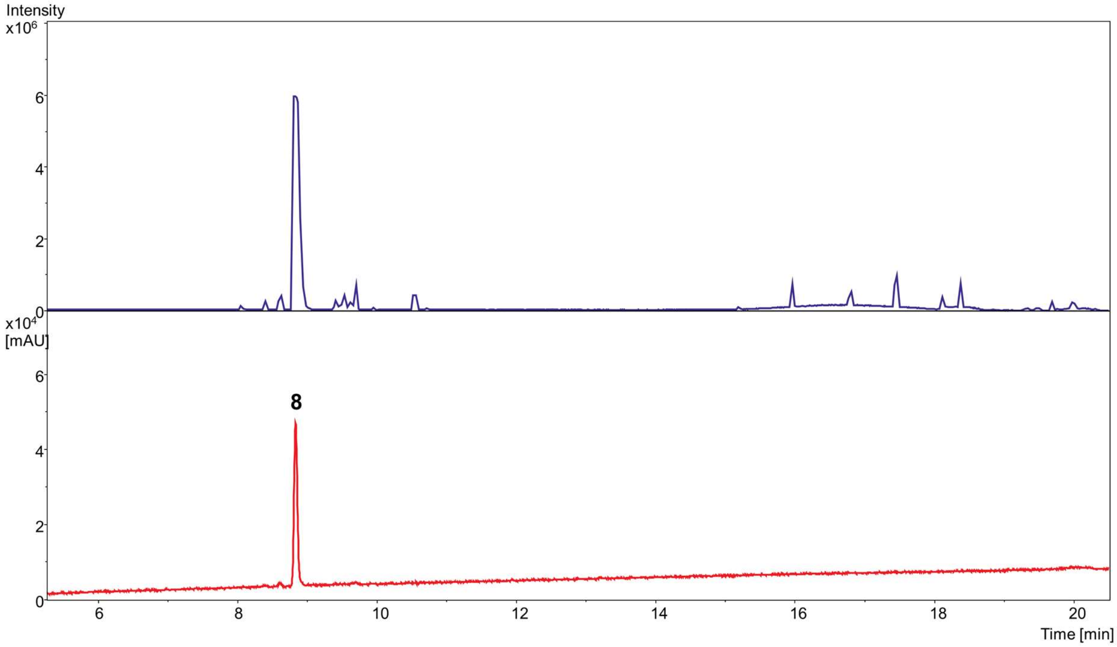


**Figure S6.** HPLC-MS BPC trace of purified **8** (top, blue) and UV/VIS (bottom, red) spectrum of **8**. Mass spectrum was acquired from 150–2000 *m*/*z*, in positive mode; UV/VIS detection by a DAD at 200–600 nm.

## 1.2 Partial ESI-MS spectra

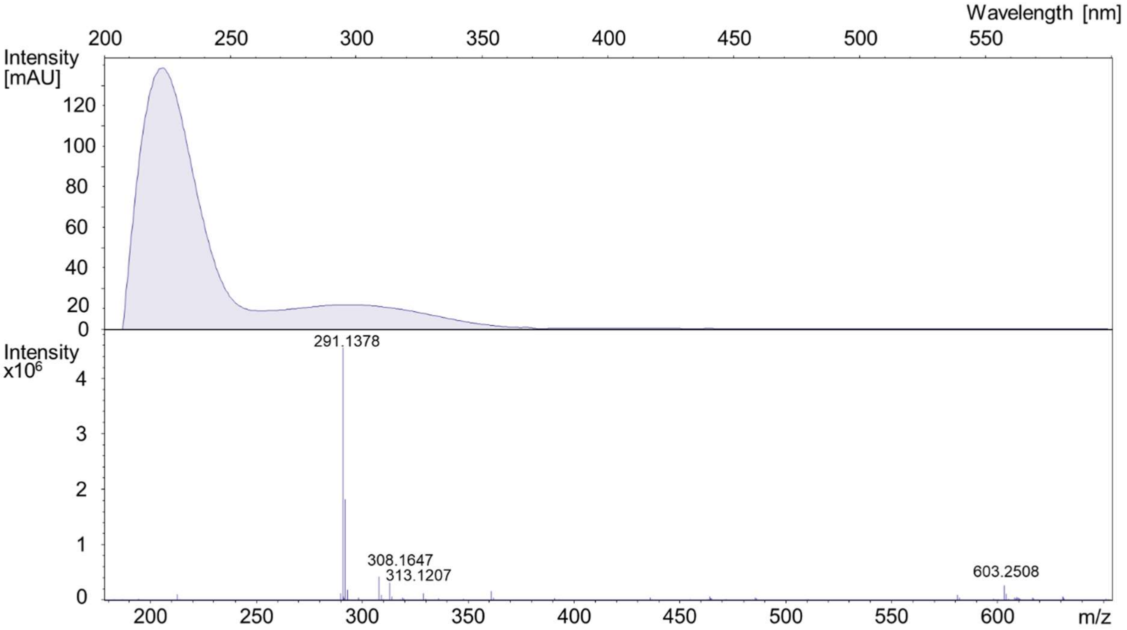


**Figure S7.** UV/VIS and partial ESI+MS spectra of purified **1** (291.1378 $[M+H]^+$, 308.1647 $[M+NH_4]^+$, 313.1207 $[M+Na]^+$, 603.2508 $[2M+Na]^+$). RT: 13.05–13.15 min.

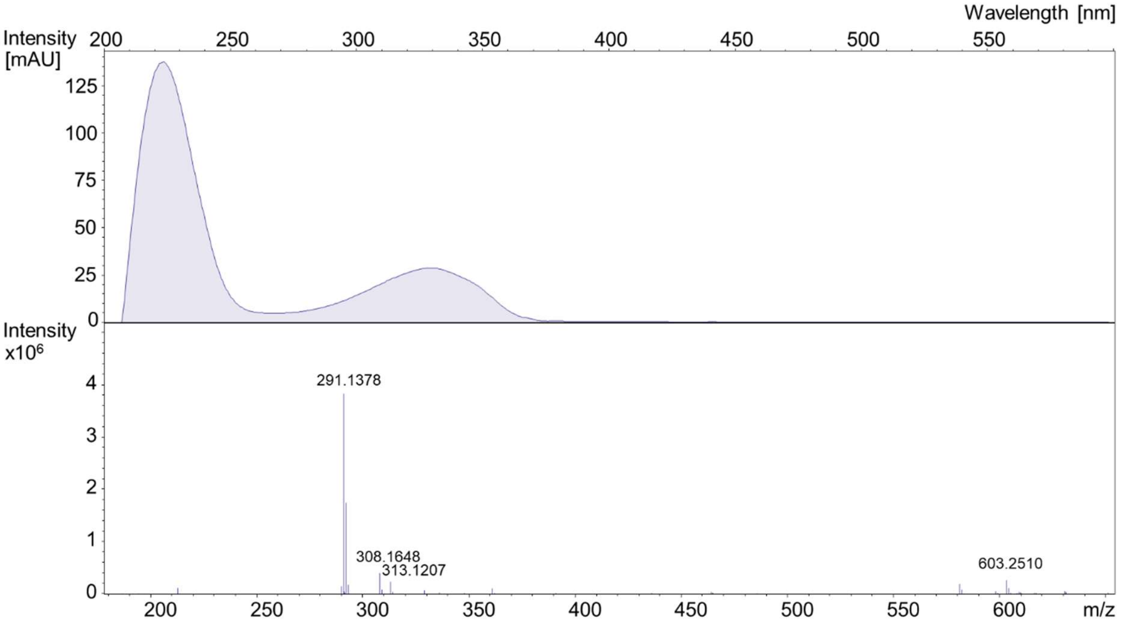


**Figure S8.** UV/VIS and partial ESI+MS spectra of purified **2**. 291.1378 $[M+H]^+$, 308.1648 $[M+NH_4]^+$, 313.1207 $[M+Na]^+$, 603.2510 $[2M+Na]^+$). RT: 13.20–13.30 min.

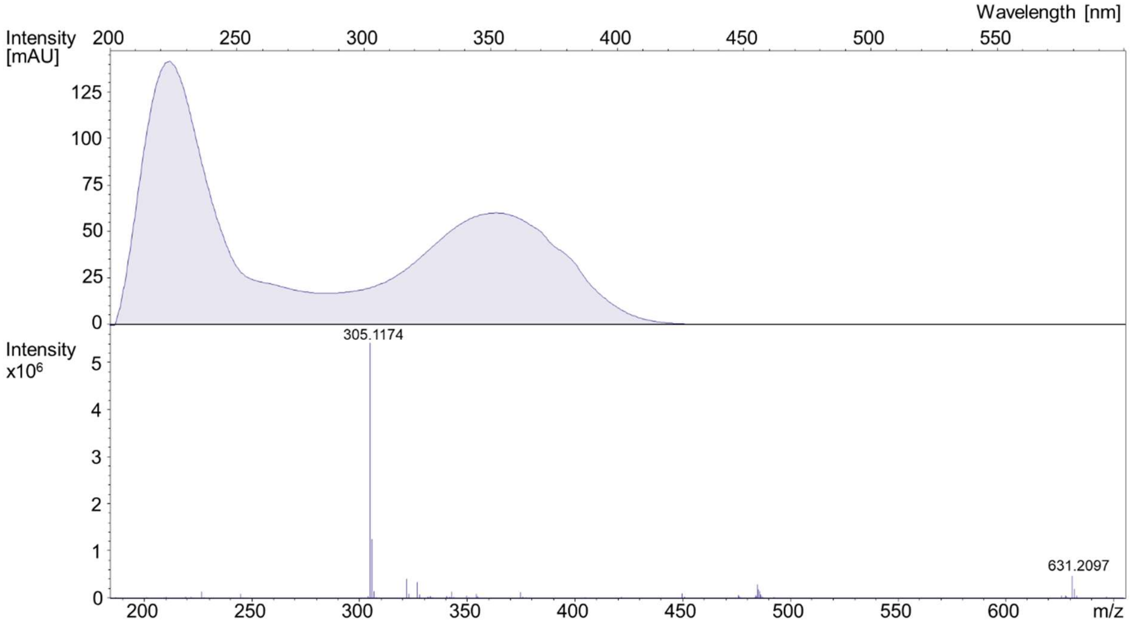


**Figure S9.** UV/VIS and partial ESI+MS spectra of purified **3**. 305.1174 $[M+H]^+$, 631.2097 $[2M+Na]^+$. RT: 12.00–12.10 min.

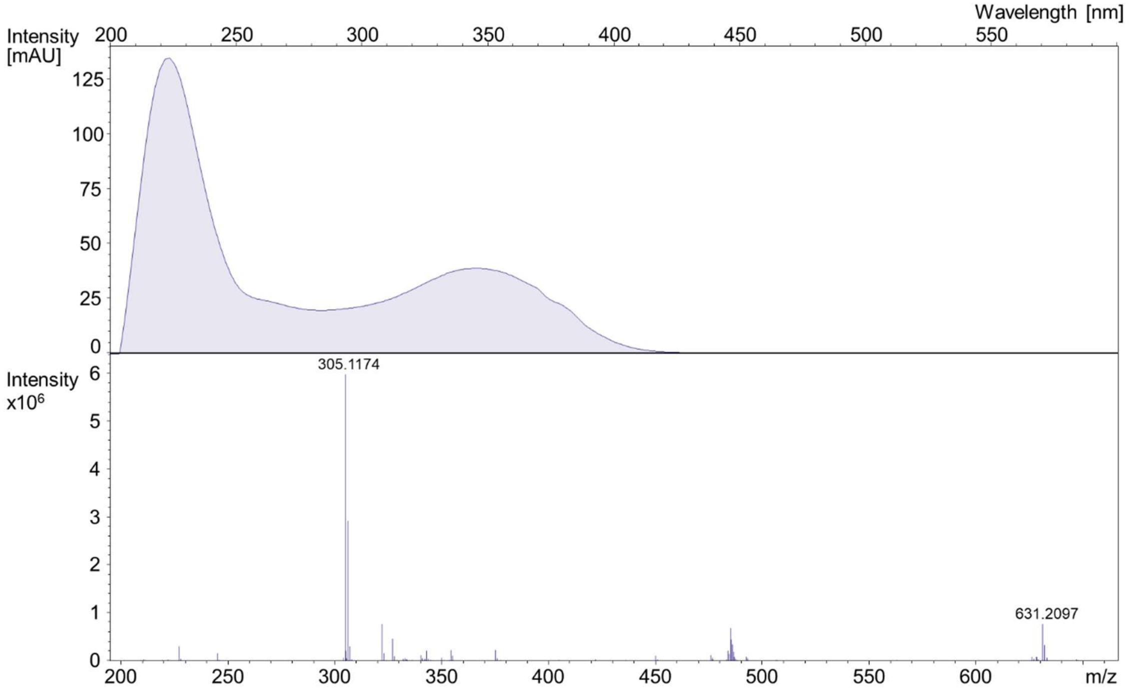


**Figure S10.** UV/VIS and partial ESI+MS spectra of purified **4**. 305.1174 $[M+H]^+$, 631.2097 $[2M+Na]^+$. RT: 12.10–12.20 min.

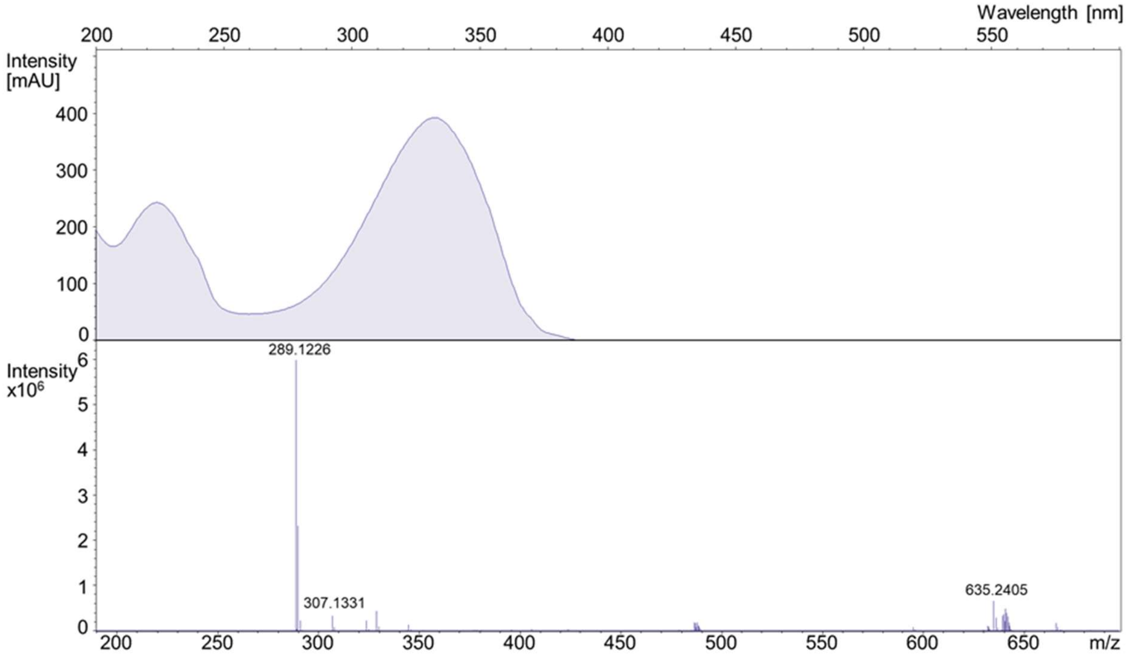


**Figure S11.** UV/VIS and partial ESI+MS spectra of purified **5**. 289.1243 [M-$H_2O$+H]$^+$, 307.1331 [M+H]$^+$, 635.2405 [2M+Na]$^+$. RT: 11.30–11.45 min.

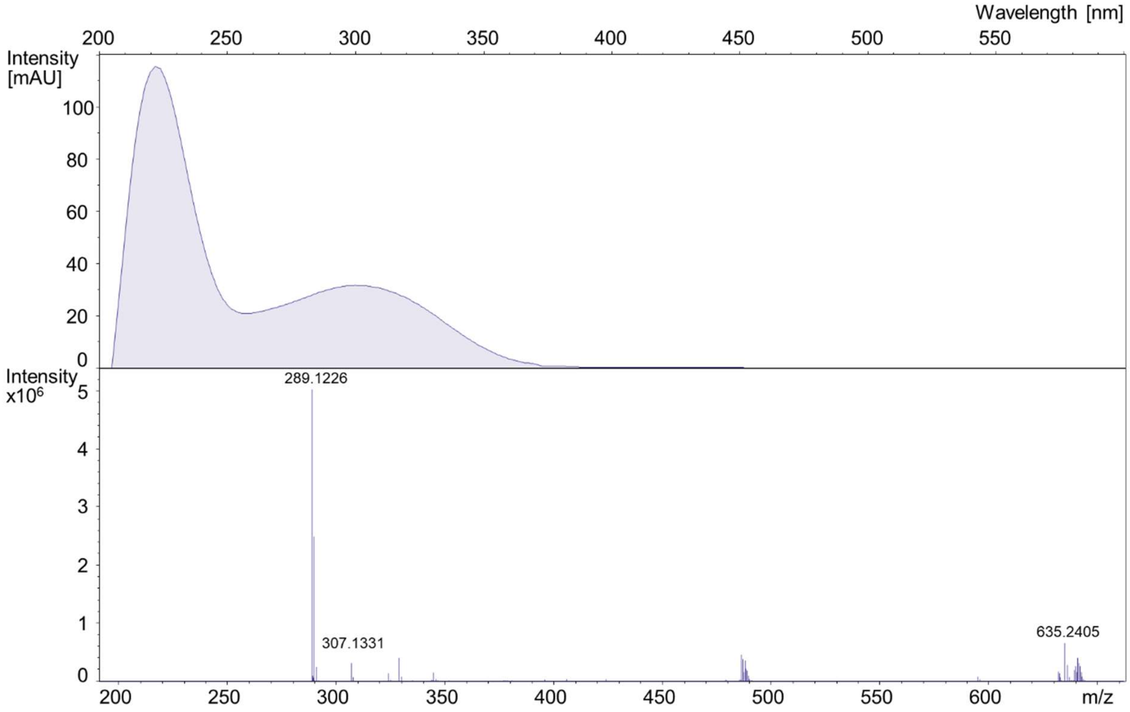


**Figure S12.** UV/VIS and partial ESI+MS spectra of purified **6**. 289.1226 [M-$H_2O$+H]$^+$, 307.1331 [M+H]$^+$, 635.2405 [2M+Na]$^+$. RT: 11.15–11.25 min.

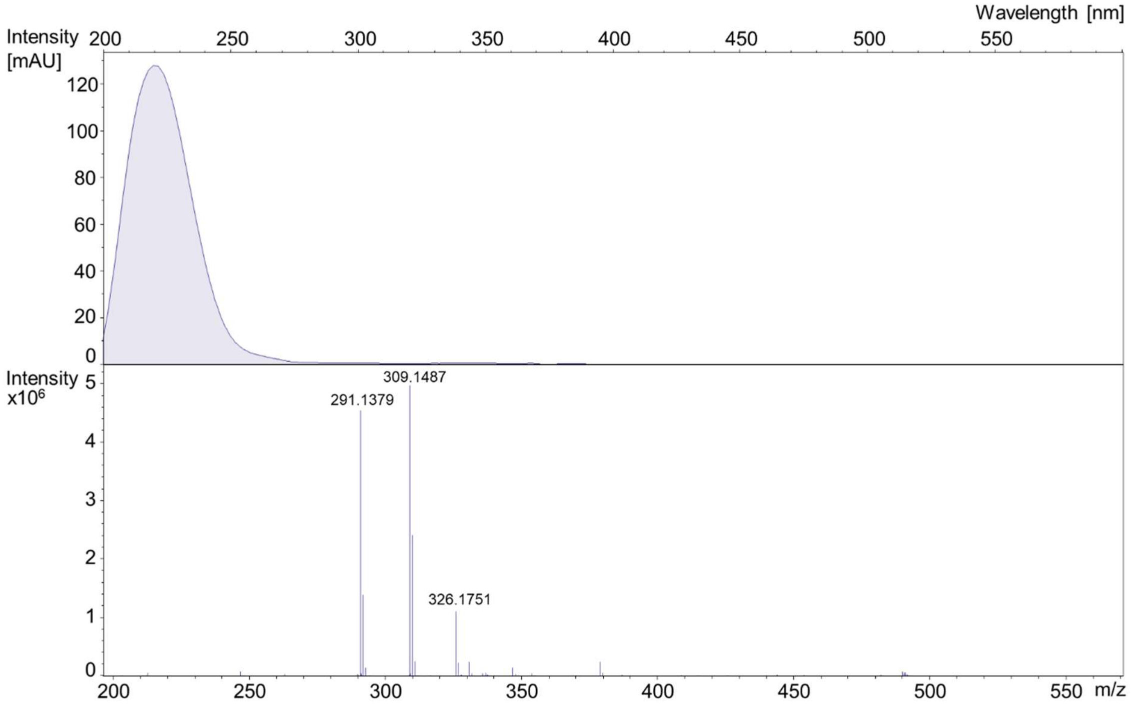


**Figure S13.** UV/VIS and partial ESI+MS spectra of purified **7**. 291.1379 [M-$H_2O$+H]$^+$, 309.1487 [M+H]$^+$, 326.1751 [M+$NH_4$]$^+$. RT: 10.38–10.48 min.

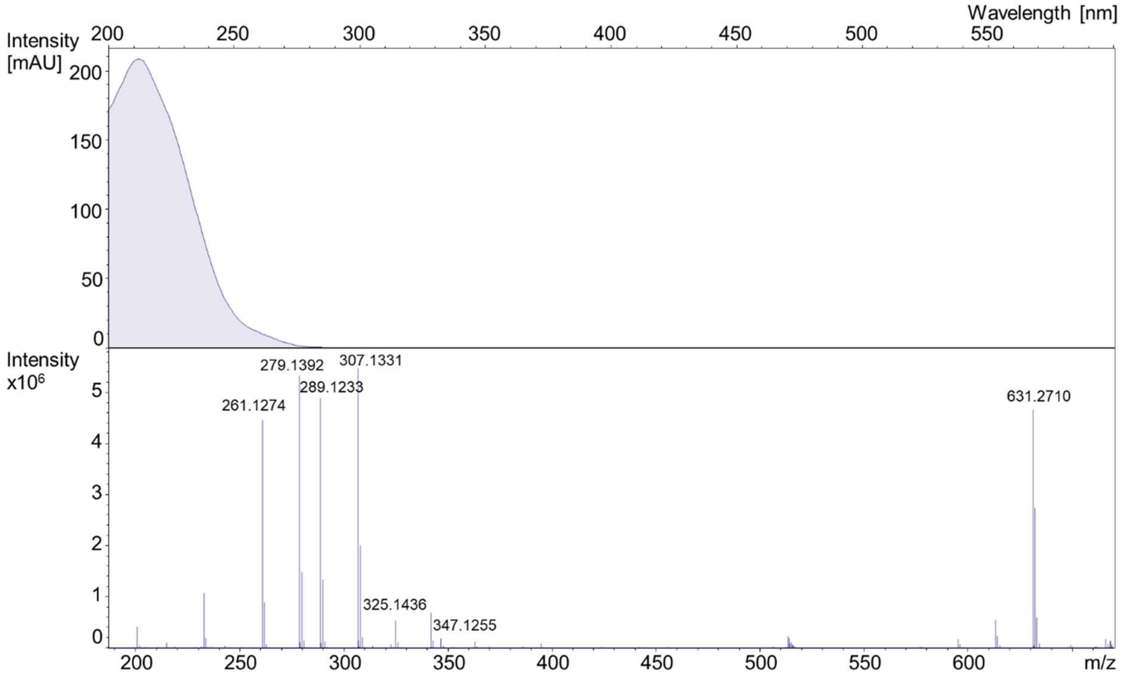


**Figure S14.** UV/VIS and partial ESI+MS spectra of purified **8**. 289.12332 [M-2$H_2O$+H]$^+$, 261.1274 [M-2$H_2O$-$C_2H_4$+H]$^+$, 307.1331 [M-$H_2O$+H]$^+$, 279.1392 [M-$H_2O$-$C_2H_4$+H]$^+$, 325.1436 [M+H]$^+$, 347.1255 [M+Na]$^+$, 631.2710 [2M-$H_2O$+H]$^+$. RT: 8.80–8.90 min.

## 2. NMR spectroscopic data for 1–8

**Table S1.** Spectroscopic values of deoxyenhygrolide C and D (**1** and **2**) acquired in $CDCl_3$ at 700 MHz.

| Position | Deoxyenhygrolide C (1) | | | | Deoxyenhygrolide D (2) | | | |
|---|---|---|---|---|---|---|---|---|
| | δC | δH [m, J (Hz)] | HMBC | ROESY | δC | δH [m, J (Hz)] | HMBC | ROESY |
| 1 | 170.7, | - | - | - | 170.1 | - | - | - |
| 2 | 126.1, | - | - | - | 130.8 | - | - | - |
| 3 | 154.9 | - | - | - | 152.8 | - | - | - |
| 4 | 148.0 | - | - | - | 149.5 | - | - | - |
| 5 | 109.1 | 6.02, s | 3, 4, (6), 7/11 | 19 | 114.7 | 6.86, s | 3, 4, (6), 7/11 | - |
| 6 | 133.3 | - | - | - | 133.2 | - | - | - |
| 7/11 | 130.6 | 7.78, m | 5, 7/11, 8/9/10 | - | 129.3 | 7.30, m | 5 | - |
| 8/10 | 128.9 | 7.38, m | 6, 7/11, 8/9/10 | - | 128.5 | 7.35, m | - | - |
| 9 | 128.9 | 7.31, m | 7/11 | - | 128.9 | 7.27, m | - | - |
| 12 | 29.8 | 3.74, s | 1, 2, 3, (4), 13, 14/18 | - | 29.7 | 3.69, s | 1, 2, 3, (4), 13, 14/18 | - |
| 13 | 138.1 | - | - | - | 137.9 | - | - | - |
| 14/18 | 128.6 | 7.27, m | 12 | - | 128.7 | 7.24, m | 12 | - |
| 15/17 | 128.9 | 7.29, m | 13 | - | 128.9 | 7.28, m | 13 | - |
| 16 | 126.8 | 7.21, m | (13), 14/18 | - | 128.9 | 7.27, m | - | - |
| 19 | 18.4 | 2.58, q, 7.7 | 2, 3, 4, 20 | 5 | 19.4 | 2.28, q, 7.6 | 2, 3, 4, 20 | - |
| 20 | 14.1 | 1.15, t, 7.7 | 3, 19 | - | 12.9 | 0.64, t, 7.6 | 3, 19 | - |

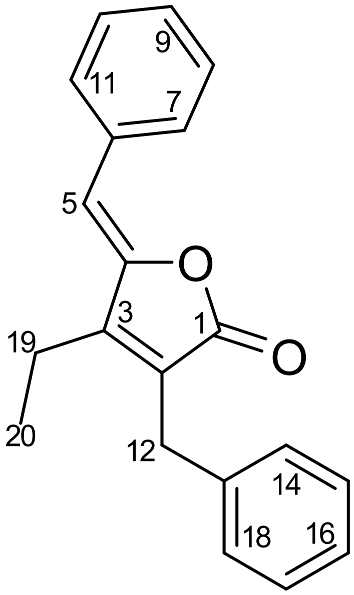


**1**

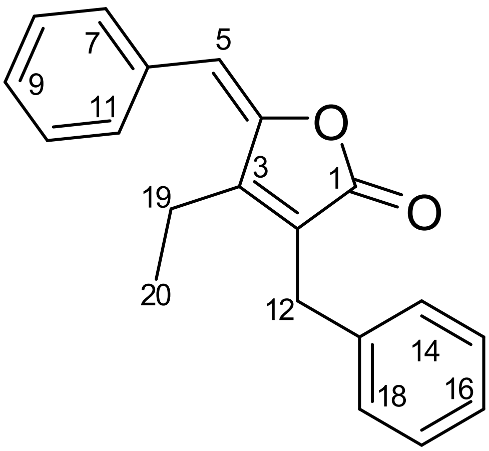


**2**

**Figure S15.** Structure and carbon numbering of deoxyenhygrolide C and D (**1** and **2**).

**Table S2.** Spectroscopic values of deoxyenhygrolide E and F (**3** and **4**) acquired in $CDCl_3$ at 500 MHz.

| **Position** | Deoxyenhygrolide E (**3**) | | | | Deoxyenhygrolide F (**4**) | | | |
|---|---|---|---|---|---|---|---|---|
| | **δC** | **δH [m, *J* (Hz)]** | **HMBC** | **ROESY** | **δC** | **δH [m, *J* (Hz)]** | **HMBC** | **ROESY** |
| 1 | 166.9 | - | - | - | 166.2 | - | - | - |
| 2 | 124.2 | - | - | - | 129.4 | - | - | - |
| 3 | 164.7 | - | - | - | 161.2 | - | - | - |
| 4 | 147.1 | - | - | - | 148.8 | - | - | - |
| 5 | 113.9 | 6.36, s | (1), 3, 4, (6), 7/11 | 19 | 119.6 | 7.16, s | 3, 4, (6), 7/11 | - |
| 6 | 132.6 | - | - | - | 132.4 | - | - | - |
| 7/11 | 131.4 | 7.88, m | 5, 7/11 | - | 129.2 | 7.37, m | 5, 6 | 19 |
| 8/10 | 129.2 | 7.45, m | 6, 8/10 | - | 129.2 | 7.44, m | 6, 8/10 | - |
| 9 | 130.1 | 7.41, m | - | - | 128.8 | 7.41, m | - | - |
| 12 | 190.0 | - | - | - | 190.2 | - | - | - |
| 13 | 136.7 | - | - | - | 136.4 | - | - | - |
| 14/18 | 129.7 | 7.88, m | 12, 14/18, 16 | - | 129.7 | 7.87, m | 12, 14/18, 16 | - |
| 15/17 | 128.8 | 7.50, m | 13, 14/18, 15/17 | - | 128.8 | 7.50, m | 13, 14/18, 15/17 | - |
| 16 | 134.2 | 7.63, m | 14/18 | - | 134.4 | 7.63, m | 14/18 | - |
| 19 | 19.2 | 2.78, q, 7.5 | (1), 2, 3, 4, 20 | 5 | 20.3 | 2.40, q, 7.5 | (1), 2, 3, 4, 20 | 7/11 |
| 20 | 15.0 | 1.32, t, 7.5 | 3, 19 | - | 13.6 | 0.80, t, 7.5 | 3, 19 | - |

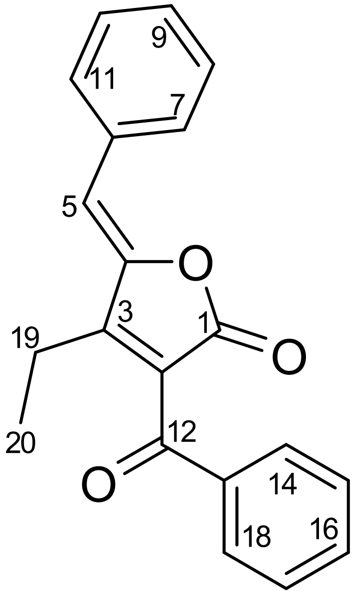


**3**

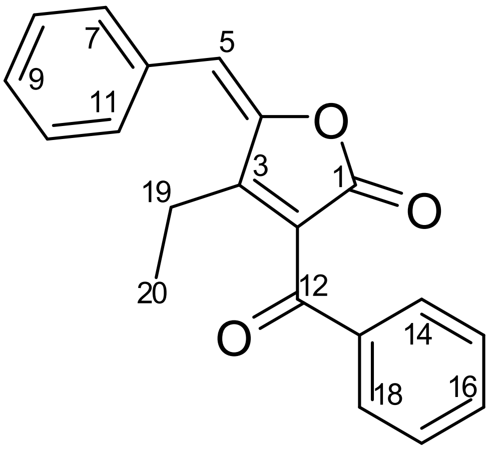


**4**

**Figure S16.** Structure and carbon numbering of deoxyenhygrolide E and F (**3** and **4**).

**Table S3.** Spectroscopic values of deoxyenhygrolide G and H (**5** and **6**) acquired in $CDCl_3$ at 700 MHz.

| **Position** | Deoxyenhygrolide G (**5**) | | | | Deoxyenhygrolide H (**6**) | | | |
|---|---|---|---|---|---|---|---|---|
| | $\delta_C$ | **$\delta_H$ [m, *J* (Hz)]** | **HMBC** | **ROESY** | $\delta_C$ | **$\delta_H$ [m, *J* (Hz)]** | **HMBC** | **ROESY** |
| 1 | 169.9 | - | - | - | 169.4 | - | - | - |
| 2 | 126.9 | - | - | - | 131.2 | - | - | - |
| 3 | 155.1 | - | - | - | 152.8 | - | - | - |
| 4 | 147.4 | - | - | - | 149.0 | - | - | - |
| 5 | 110.8 | 6.11, s | (1), 3, 4, 7/11 | 7/11, 19 | 116.8 | 6.97, s | 3, 4, (6), 7/11, (12) | - |
| 6 | 132.9 | - | - | - | 132.7 | - | - | - |
| 7/11 | 130.8 | 7.78, m | 5, 9, 7/11 | 5 | 129.2 | 7.31, m | 5, 9, 8/10 | - |
| 8/10 | 129.0 | 7.39, m | 6, 7/11 ,8/10 | - | 128.6 | 7.35–7.39, m | - | - |
| 9 | 129.3 | 7.33, m | 6, 7/11 | - | 128.8 | 7.35–7.39, m | - | - |
| 12 | 68.9 | 5.78, br s | 1, 2, 3, 13, 14/18 | 19 | 68.9 | 5.69, br s | 1, 2, 3, 13, 14/18 | 19 |
| 12-OH | - | 3.44, br s | (2) | - | - | 3.64, br s | 2, 12, (13) | - |
| 13 | 141.5 | - | - | - | 141.5 | - | - | - |
| 14/18 | 126.1 | 7.46, m | 12, 14/18, 16 | - | 126.2 | 7.42, m | 12, 14/18, 16 | - |
| 15/17 | 128.9 | 7.37, m | 13, (14/18), 15/17 | - | 128.9 | 7.36, m | 13, (14/18), 15/17 | - |
| 16 | 128.2 | 7.30, m | (13), 14/18 | - | 128.3 | 7.30, m | 14/18, 15/17 | - |
| 19 | 18.2 | 2.62, q, 7.7 | (1), 2, 3, 4, 20 | 5, 12 | 19.2 | 2.32, q, 7.6 | (1), 2, 3, 4, 20 | 12 |
| 20 | 14.4 | 1.17, t, 7.7 | 3, 19 | - | 13.0 | 0.67, t, 7.6 | 3, 19 | - |

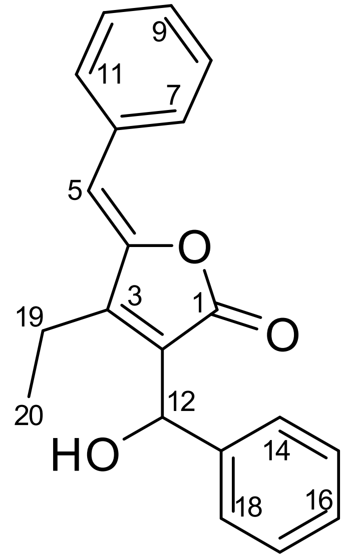


**5**

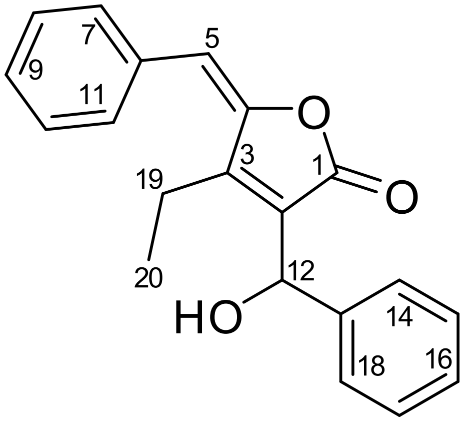


**6**

**Figure S17.** Structure and carbon numbering of deoxyenhygrolide G and H (**5** and **6**).

**Table S4.** Spectroscopic values of deoxyenhygrolide I and J (**7** and **8**) acquired in $CD_3OD$ at 700/500 MHz, respectively.

| **Position** | Deoxyenhygrolide I (**7**) | | | Deoxyenhygrolide J (**8**) | | |
|---|---|---|---|---|---|---|
| | **δC** | **δH [m, *J* (Hz)]** | **HMBC** | **δC** | **δH [m, *J* (Hz)]** | **HMBC** |
| 1 | 173.5 | - | - | 172.8 | - | - |
| 2 | 129.3 | - | - | 132.3 | - | - |
| 3 | 165.2 | - | - | 166.4 | - | - |
| 4 | 109.1 | - | - | 109.3 | - | - |
| 5 | 43.2 | 3.21, br s<br>3.35, br s | - | 43.6 | 3.21, br s<br>3.33, br s | - |
| 6 | 135.4 | - | - | 135.5 | - | - |
| 7/11 | 131.1 | 7.17, m | 5, 9, 7/11 | 128.2 | 7.23, m | (5), 9, 8/10 |
| 8/10 | 129.0 | 7.20, m | 6 ,8/10 | 129.4 | 7.20, m | 6 ,8/10 |
| 9 | 127.9 | 7.23, m | 7/11, (8/10) | 131.4 | 7.15, m | - |
| 12 | 29.3 | 3.42, br s | 1, 2, 3, 13, 14/18 | 68.2 | 5.43, br s | 1, 2, 3, 13, 14/18 |
| 13 | 138.5 | - | - | 142.7 | - | - |
| 14/18 | 128.8 | 6.65, m | 12, (13), 14/18, 16 | 126.9 | 6.81, br s | - |
| 15/17 | 129.1 | 7.09, m | 13, 15/17 | 129.2 | 7.15, m | (12), 13, 15/17 |
| 16 | 126.8 | 7.09, m | - | 128.1 | 7.15, m | 14/18 |
| 19 | 20.6 | 2.42, q, 7.7 | 2, 3, 4, 20 | 20.8 | 2.48, q, 7.6 | 2, 3, 4, 20 |
| 20 | 12.1 | 1.17, t, 7.7 | 3, 19 | 13.4 | 1.22, t, 7.6 | 3, 19 |

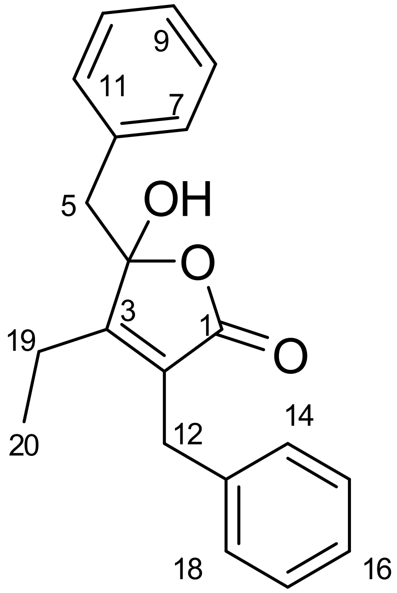


**7**

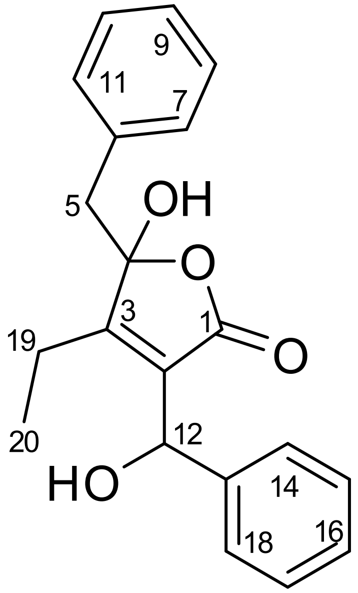


**8**

**Figure S18.** Structure and carbon numbering of deoxyenhygrolide I and J (**7** and **8**).

*marine drugs*

**Table S5.** Comparison of [13]C chemical shifts for **1–8**.

| | **1** | **2** | **3** | **4** | **5** | **6** | **7** | **8** |
|---|---|---|---|---|---|---|---|---|
| **Pos.** | **$CDCl_3$** | **$CDCl_3$** | **$CDCl_3$** | **$CDCl_3$** | **$CDCl_3$** | **$CDCl_3$** | **$CD_3OD$** | **$CD_3OD$** |
| 1 | 170.7 | 170.1 | 166.9 | 166.2 | 169.9 | 169.4 | 173.5 | 172.8 |
| 2 | 126.1 | 130.8 | 124.2 | 129.4 | 126.9 | 131.2 | 129.3 | 132.3 |
| 3 | 154.9 | 152.8 | 164.7 | 161.2 | 155.1 | 152.8 | 165.2 | 166.4 |
| 4 | 148.0 | 149.5 | 147.1 | 148.8 | 147.4 | 149.0 | 109.1 | 109.3 |
| 5 | 109.1 | 114.7 | 113.9 | 119.6 | 110.8 | 116.8 | 43.2 | 43.6 |
| 6 | 133.3 | 133.2 | 132.6 | 132.4 | 132.9 | 132.7 | 135.4 | 135.5 |
| 7/11 | 130.6 | 129.3 | 131.4 | 129.2 | 130.8 | 129.2 | 131.1 | 128.2 |
| 8/10 | 128.9 | 128.5 | 129.2 | 129.2 | 129.0 | 128.6 | 129.0 | 129.4 |
| 9 | 128.9 | 128.9 | 130.1 | 128.8 | 129.3 | 128.8 | 127.9 | 131.4 |
| 12 | 29.8 | 29.7 | 190.0 | 190.2 | 68.9 | 68.9 | 29.3 | 68.2 |
| 13 | 138.1 | 137.9 | 136.7 | 136.4 | 141.5 | 141.5 | 138.5 | 142.7 |
| 14/18 | 128.6 | 128.7 | 129.7 | 129.7 | 126.1 | 126.2 | 128.8 | 126.9 |
| 15/17 | 128.9 | 128.9 | 128.8 | 128.8 | 128.9 | 128.9 | 129.1 | 129.2 |
| 16 | 126.8 | 128.9 | 134.2 | 134.4 | 128.2 | 128.3 | 126.8 | 128.1 |
| 19 | 18.4 | 19.4 | 19.2 | 20.3 | 18.2 | 19.2 | 20.6 | 20.8 |
| 20 | 14.1 | 12.9 | 15.0 | 13.6 | 14.4 | 13.0 | 12.1 | 13.4 |

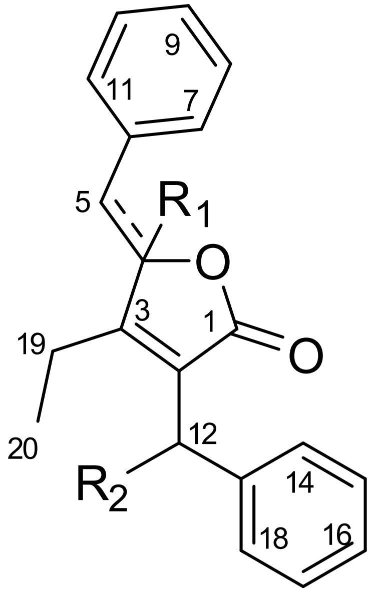


**1-8**

**Figure S19.** General structure and carbon numbering of deoxyenhygrolide A–H (**1**–**8**). (**1**) R1: –H, R2: –H, (*Z*); (**2**) R1: –H, R2: –H, (*E*); (**3**) R1: –H, R2: =O, (*Z*); (**4**) R1: –H, R2: =O, (*E*); (**5**) R1: –H, R2: –OH, (*E*); (**6**) R1: –H, R2: –OH, (*Z*); (**7**) R1: –OH, R2: –H; (**8**) R1: –OH, R2: –OH.

# 3. Genetic and biosynthetic investigations

## 3.1 Identification of *cybE* and *cybF* homologs in different myxobacteria

The myxobacterial gene homologs of *cybE* and *cybF* were identified in *Enhygromyxa salina* SWB005 (GenBank accession number: PVNK00000000.1) on contig000249 (GenBank accession number: PVNK01000249.1), in *Enhygromyxa salina* SWB007 (GenBank accession number: PVNL00000000.1) on contig000074 (GenBank accession number: PVNL01000074.1) and in *Plesiocystis pacifica* DSM 14875$^T$ (GenBank accession number: ABCS00000000.1) on contig1000004 (GenBank accession number: ABCS01000004.1). Interestingly, between the co-localized gene homologs *cybE* and *cybF* in *E. salina* SWB007 (not in *E. salina* SWB005), an open reading frame (ORF) is located, which is putatively encoding a homolog of a flavin-dependent tryptophan halogenase (PrnA) which was described in the biosynthesis of pyrrolnitrin [1]. The size in base pairs (bp) and locus tag of the respective *cybE* or *cybF* are given in **Table S6**.

**Table S6.** *cybE* and *cybF* homologs identified in different myxobacterial genome sequences.

| | *cybE* | | *cybF* | |
|---|---|---|---|---|
| **Organism** | **Locus Tag** | **Size [bp]** | **Locus Tag** | **Size [bp]** |
| *Tolypothrix sp.* PCC 9009 | *Tol9009DRAFT_00039900* | 1872 | *Tol9009DRAFT_00039890* | 1131 |
| *Plesiocystis pacifica* DSM 14875$^T$ | PPSIR1_40640 | 1536 | PPSIR1_40635 | 1107 |
| *Enhygromyxa salina* SWB005 | ENSA5_56740 | 1674 | ENSA5_56750 | 1113 |
| *Enhygromyxa salina* SWB007 | 151239–152885(1) | 1647 | ENSA7_38410 | 1125 |

(1) No gene annotation available; interval of open reading frame is provided

Since the gene annotation in the publicly available myxobacterial genome sequence display shorter *cybE* homologs (1536/1674 bp vs. 1872 bp) or no gene annotation at all (SWB007), we further investigated the genome sequence of *P. pacifica* DSM 14875$^T$ for the presence of a longer open reading frame and annotated the *cybE* homolog accordingly. The slightly different annotation is presented in **Table S7** alongside the pairwise sequence identity and positive percentage using BLSM62 substitution-scoring matrix [2] obtained by a pairwise alignment with *CybE* from *Tolypothrix sp.* PCC 9009.

**Table S7.** Changed locus tag of *cybE* homolog.

| Organism | Locus Tag | Size [bp] | Identity | Similarity |
|---|---|---|---|---|
| *Tolypothrix sp.* PCC 9009 | *Tol9009DRAFT_00039900* | 1872 | --- | --- |
| *P. pacifica* DSM 14875$^T$ | 134868–136562 | 1695 | 44.0% | 61.6% |
| *E. salina* SWB005 | ENSA5_56740 | 1674 | 43.3% | 60.8% |
| *E. salina* SWB007 | 151239–152885(1) | 1647 | 44.2% | 61.8% |

(1) (No) gene annotation available; interval of open reading frame is provided

Amino acid sequence alignment of the CybE/F homolog from *P. pacifica* DSM 14875$^T$ (see **3.2**) show comparatively low but significant similarity to the *in vitro* investigated thiamine pyrophosphate (TPP)-binding protein CybE from *Tolypothrix* sp. PCC 9009 [3]. In addition to support the assigned function of the identified CybE homolog from *P. pacifica* DSM 14875$^T$, both CybE sequences were evaluated by *in silico* protein homology analogy recognition engine 2 (Phyre2), and the results indeed underline the structural similarity of both CybE enzymes to a structurally characterized flavin-dependent pyruvate oxidase from *Escherichia coli* (*Ec*POX, PDB: 3EY9) [4]. In contrast, Phyre2 analysis of both CybF enyzmes led to slightly different homology models; the CybE homolog from *P. pacifica* DSM 14875$^T$ displays *in silico* structural similarity to a 3-Ketoacyl-ACP synthase **(**KAS III) from *Propionibacterium acnes* (PDB: 6A9N, [5]), whereas CybE from *Tolypothrix* sp. PCC 9009 seems structurally closer related to KAS III from *Vibrio cholerae* (PDB: 4X0O, [6]).

**3.2** Sequence alignments and Phyre2 structures.

**CybE**

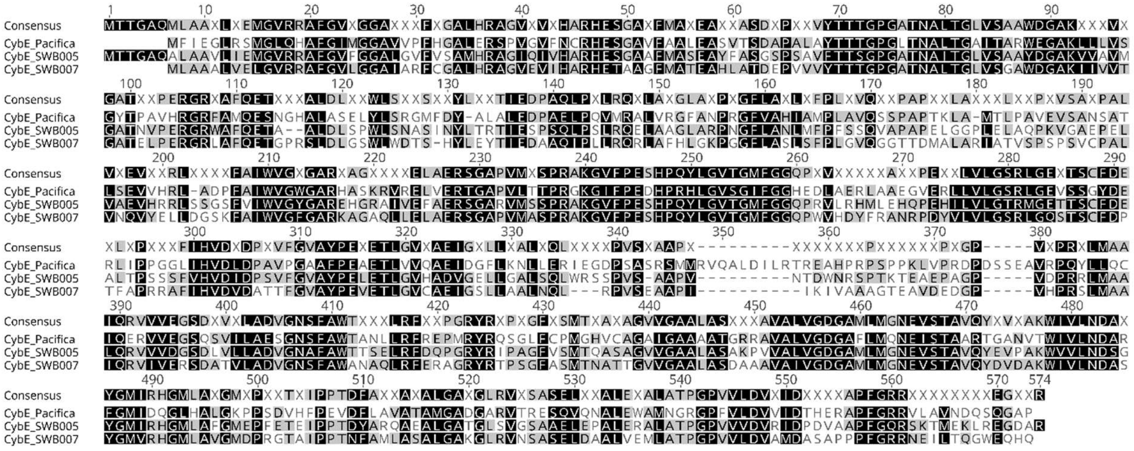

**Figure S20.** Amino acid alignment of myxobacterial CybE homologs from *P. pacifica* DSM 14875$^T$ (CybE_Pacifica), *E. salina* SWB005 (CybE_SWB005) and *E. salina* SWB007 (CybE_SWB007). Pairwise identity: 45.0%, pairwise positive (BLSM62): 61.2%.

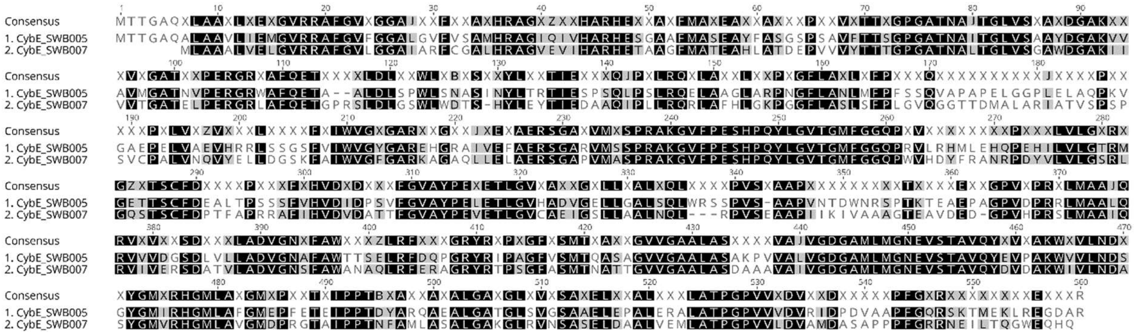

**Figure S21.** Amino acid alignment of myxobacterial CybE homologs from *E. salina* SWB005 (CybE_SWB005) and *E. salina* SWB007 (CybE_SWB007). Pairwise identity: 57.3%, pairwise positive (BLSM62): 72.3%.

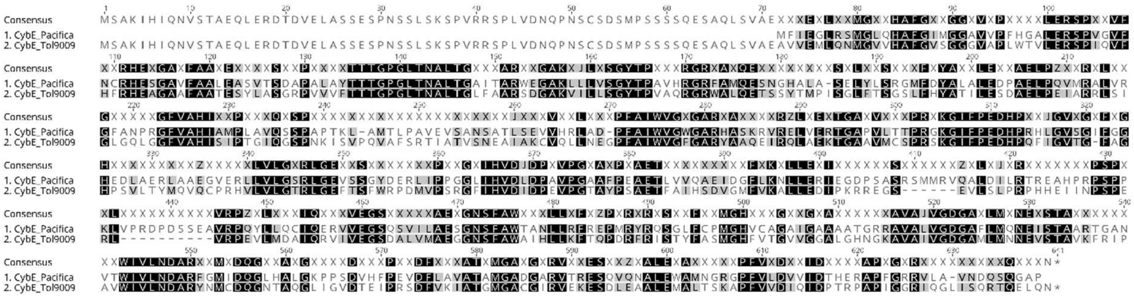

**Figure S22.** Amino acid alignment of myxobacterial CybE homolog from from *P. pacifica* DSM 14875$^T$ (CybE_Pacifica) and CybE from *Tolypothrix sp.* PCC 9009 (CybE_Tol9009). Pairwise identity: 44.0%, pairwise positive (BLSM62): 61.6%.

## CybF

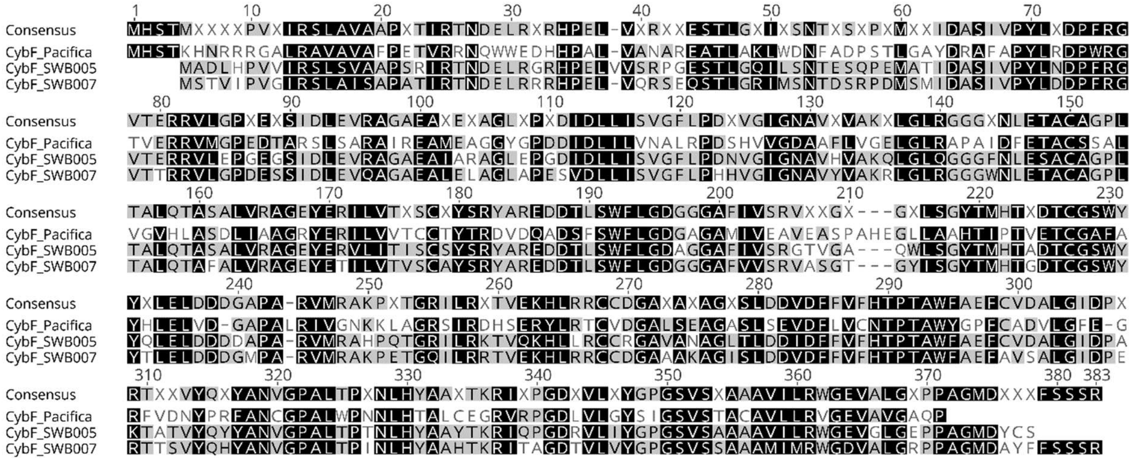


**Figure S23.** Amino acid alignment of myxobacterial CybF homologs from *P. pacifica* DSM 14875$^{T}$ (CybF_Pacifica), *E. salina* SWB005 (CybF_SWB005) and *E. salina* SWB007 (CybF_SWB007). Pairwise identity: 50.8%, pairwise positive (BLSM62): 66.5%.

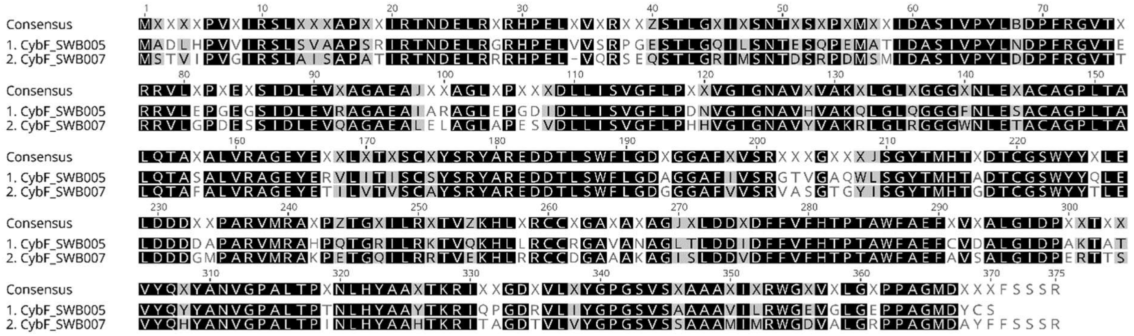


**Figure S24.** Amino acid alignment of myxobacterial CybF homologs from *E. salina* SWB005 (CybF_SWB005) and *E. salina* SWB007 (CybF_SWB007). Pairwise identity: 74.1%, pairwise positive (BLSM62): 86.5%.

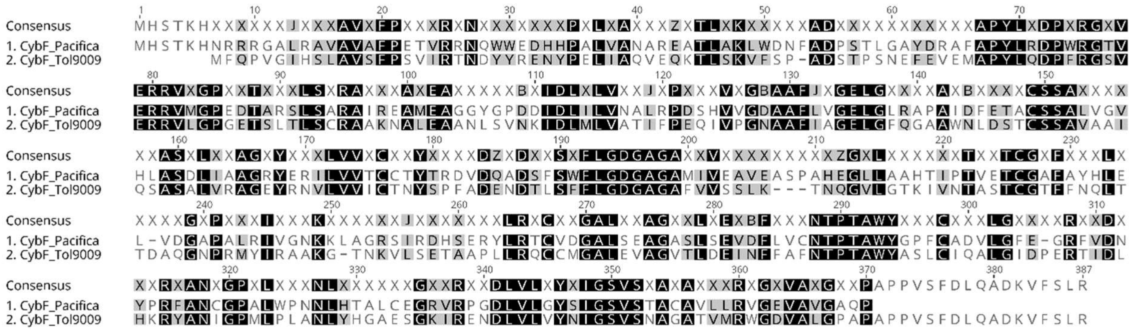


**Figure S25.** Amino acid alignment of myxobacterial CybF homolog from from *P. pacifica* DSM 14875$^{T}$ (CybF_Pacifica) and CybF from *Tolypothrix sp.* PCC 9009 (CybE_Tol9009). Pairwise identity: 39.3%, pairwise positive (BLSM62): 59.6%.

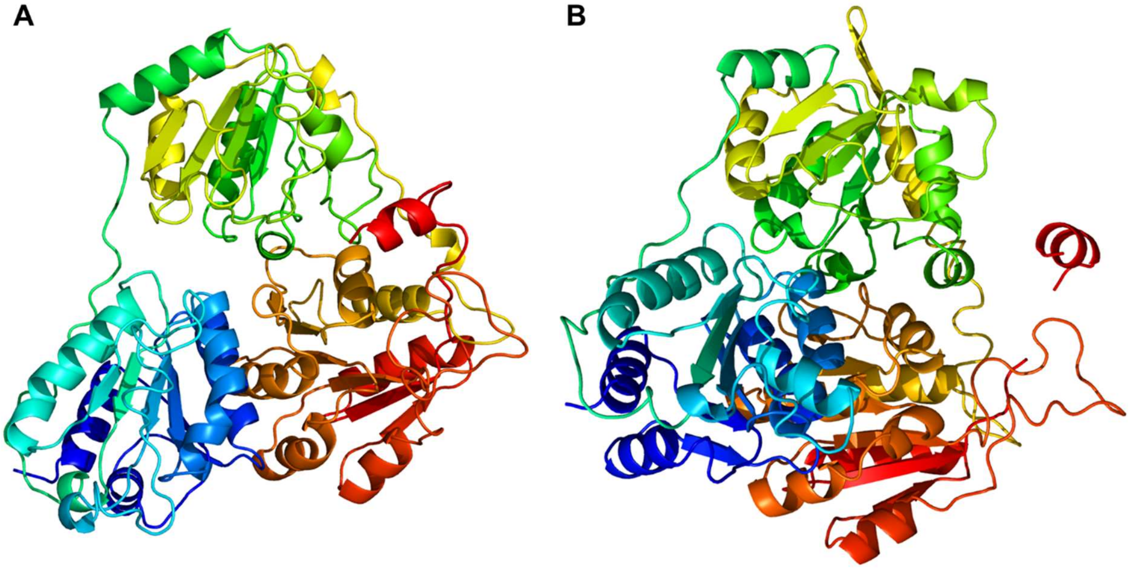


**Figure S26.** Phyre2 structure homology model of the thiamine pyrophosphate-binding protein (TPP) encoded by *cybE* from *Tolypothrix sp.* PCC 9009 (**A**) and the identified *cybE* homolog from *P. pacifica* DSM 14875$^{T}$ (models are based on the template c3ey9B; figure colored by rainbow $N \rightarrow C$ terminus).

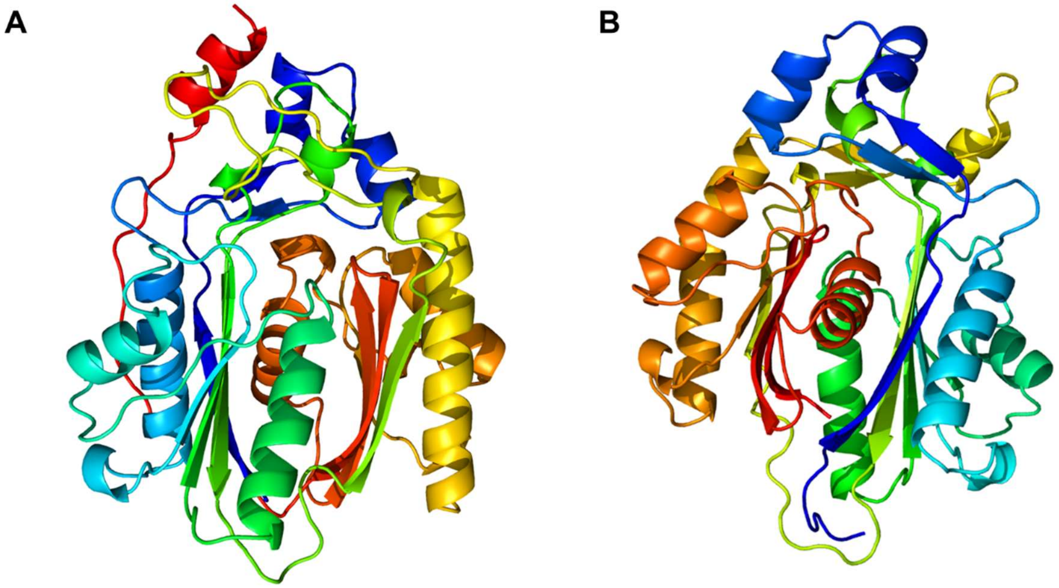


**Figure S27.** Phyre2 structure homology model of the furanolide encoded by *cybF* from *Tolypothrix sp.* PCC 9009 (**A**) and the identified *cybF* homolog from *Plesiocystis pacifica* DSM 14875$^{T}$. **A**) Model based on template c4x0oG. **B**) Model based on template c6a9nA; figure colored by rainbow $N \rightarrow C$ terminus).

# 4. $^{1}H$ and $^{13}C$ NMR spectra for **1–8**

## 4.1 NMR spectra of **1** and **2**

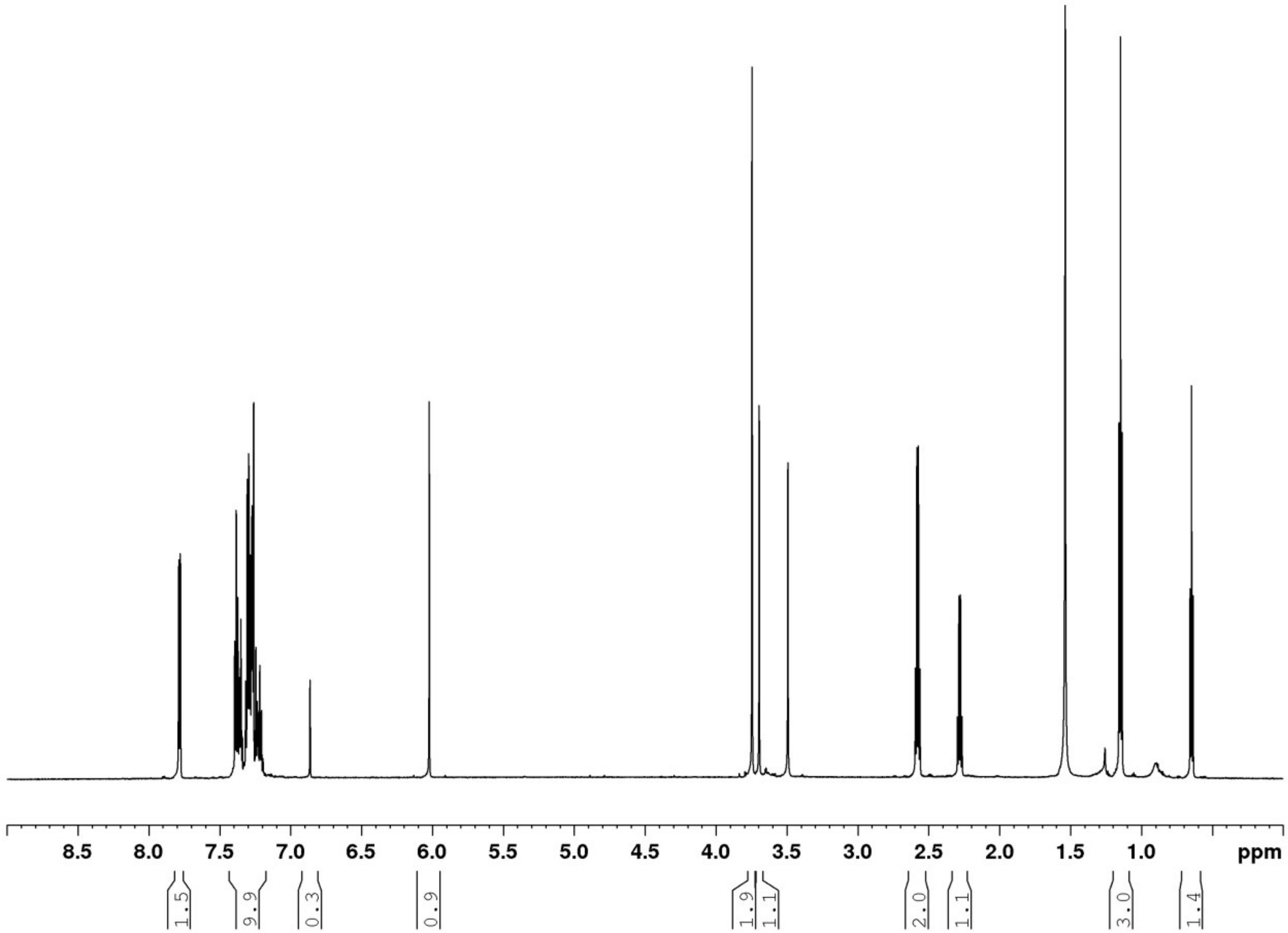


**Figure S28.** $^1$H NMR spectrum of **1** and **2** (700 MHz, $CDCl_3$).

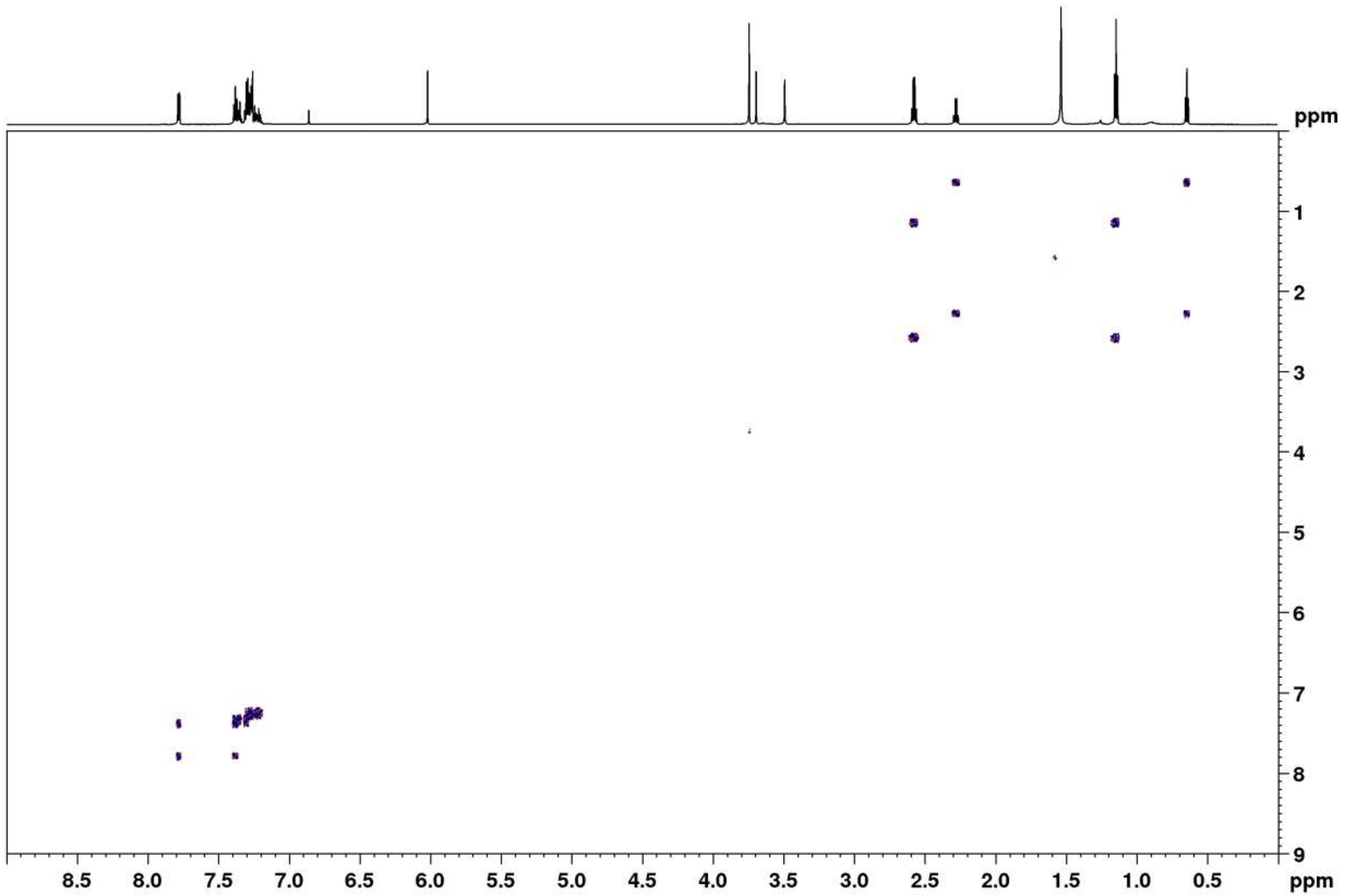


**Figure S29.** DQF-COSY spectrum of **1** and **2** (700 MHz, $CDCl_3$).

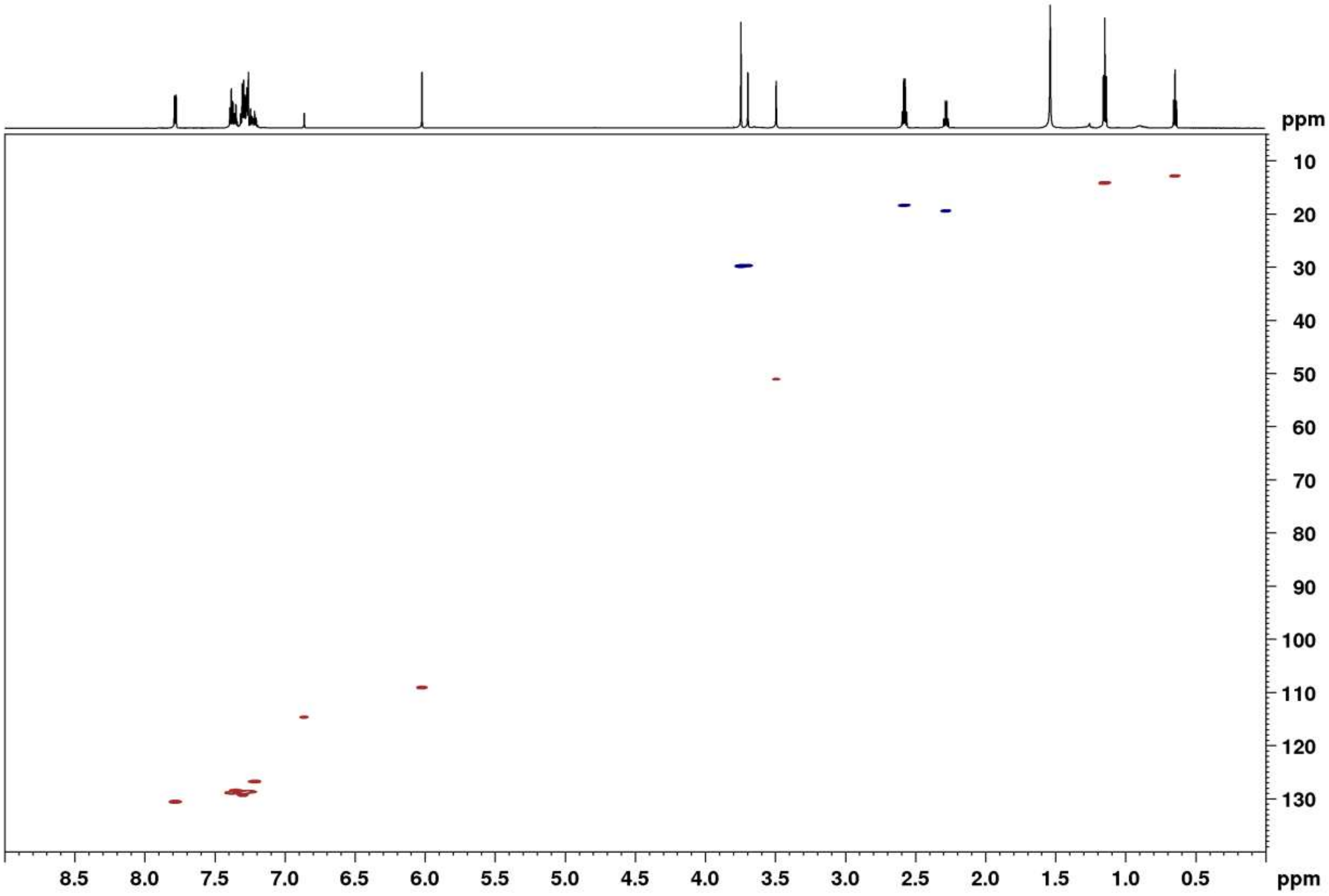


**Figure S30.** HSQC spectrum of **1** and **2** (700 MHz, $CDCl_3$).

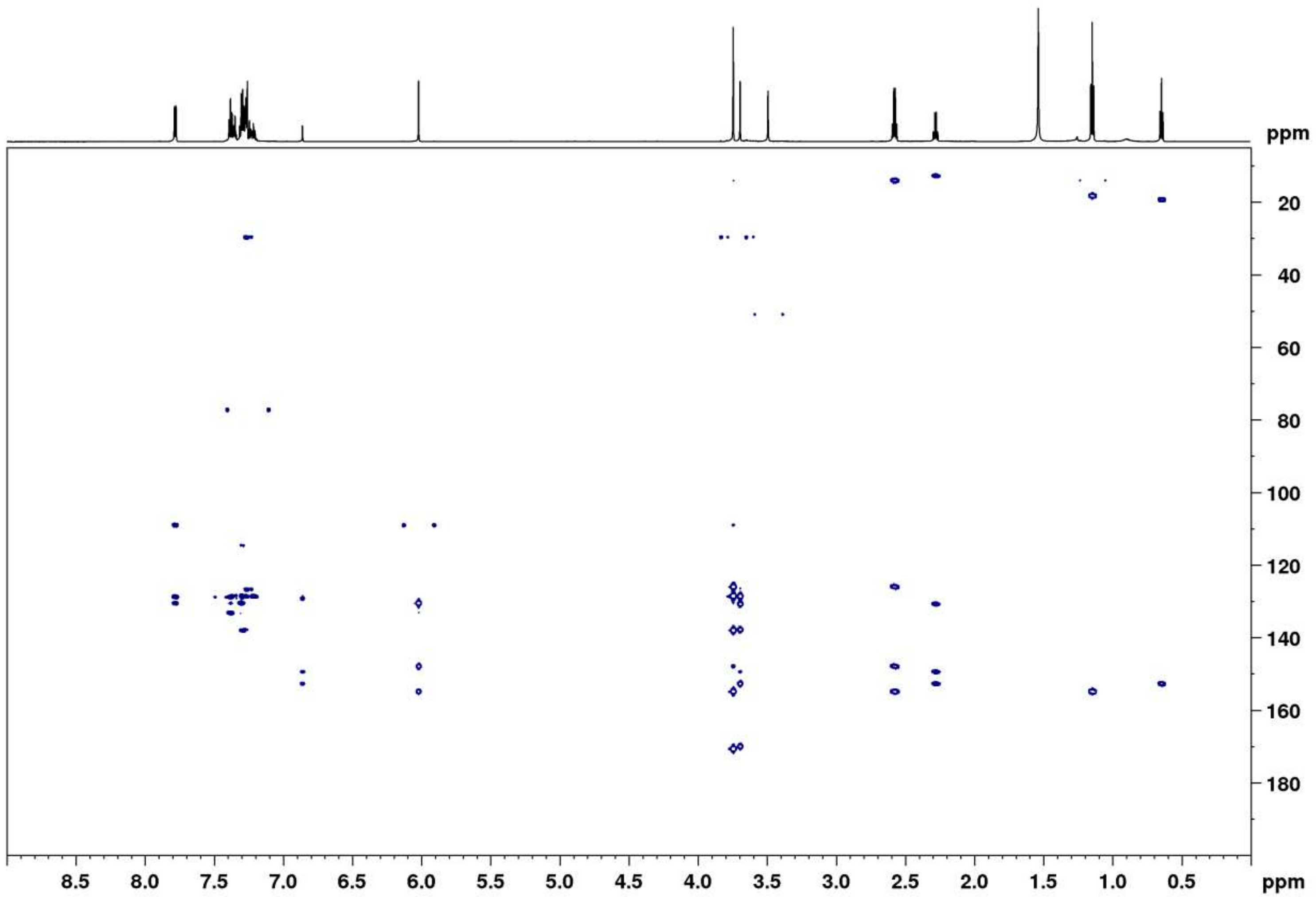


**Figure S31.** HMBC spectrum of **1** and **2** (700 MHz, $CDCl_3$).

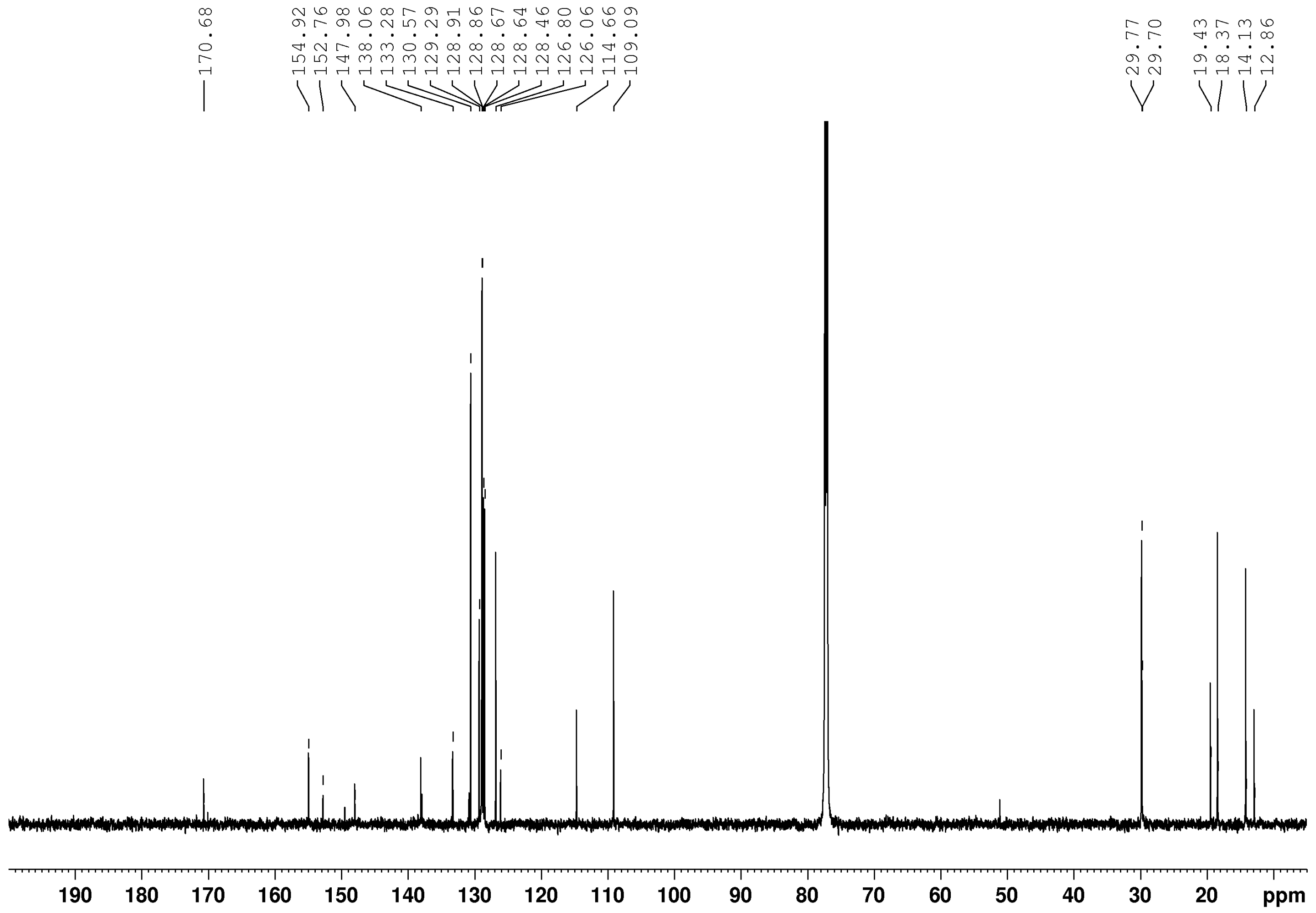


**Figure S32.** $^{13}C$ NMR spectrum of **1** and **2** (700 MHz, $CDCl_3$).

4.2 NMR spectra of **3** and **4**

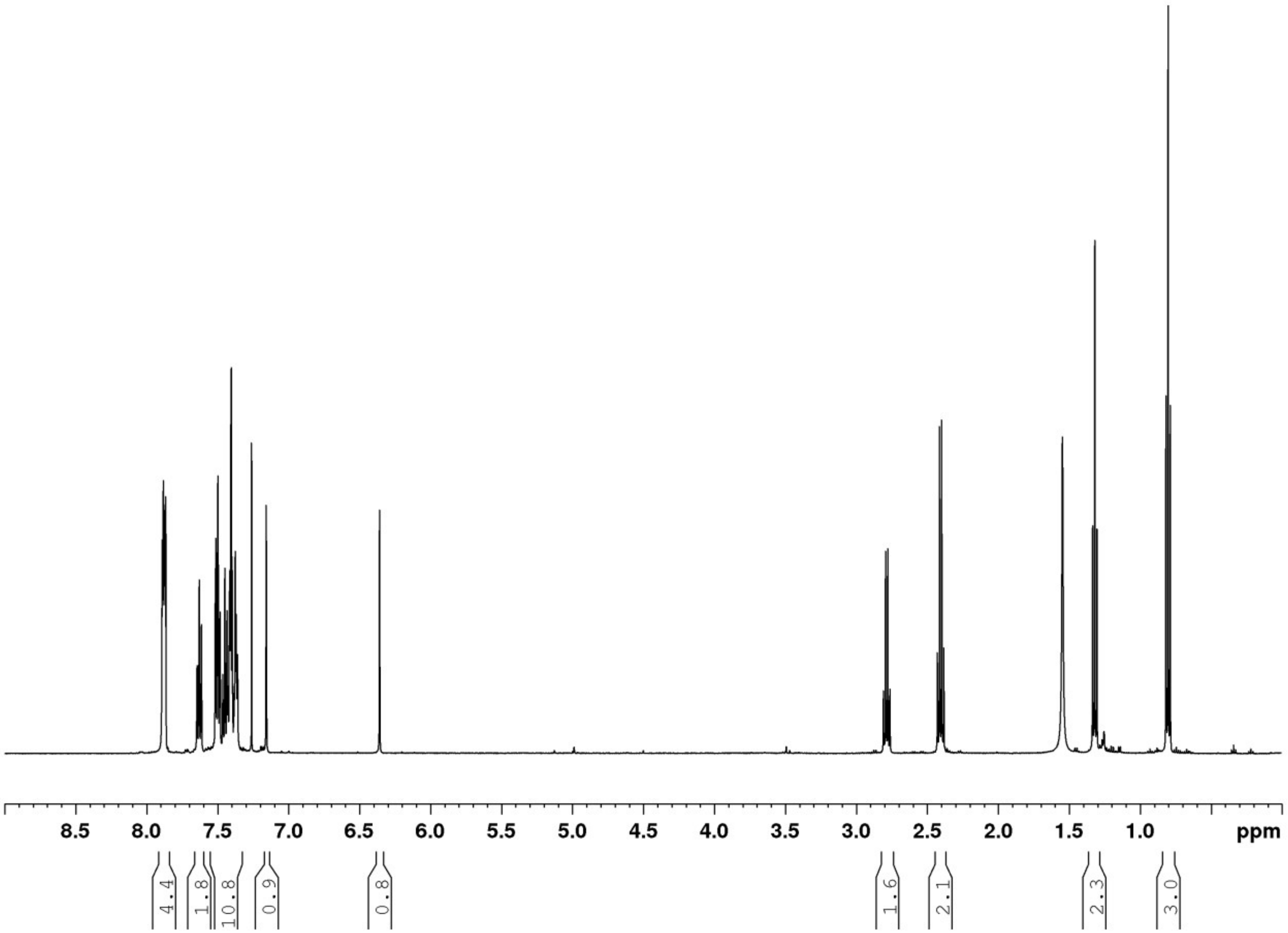


**Figure S33.** $^1$H NMR spectrum of **3** and **4** (500 MHz, $CDCl_3$).

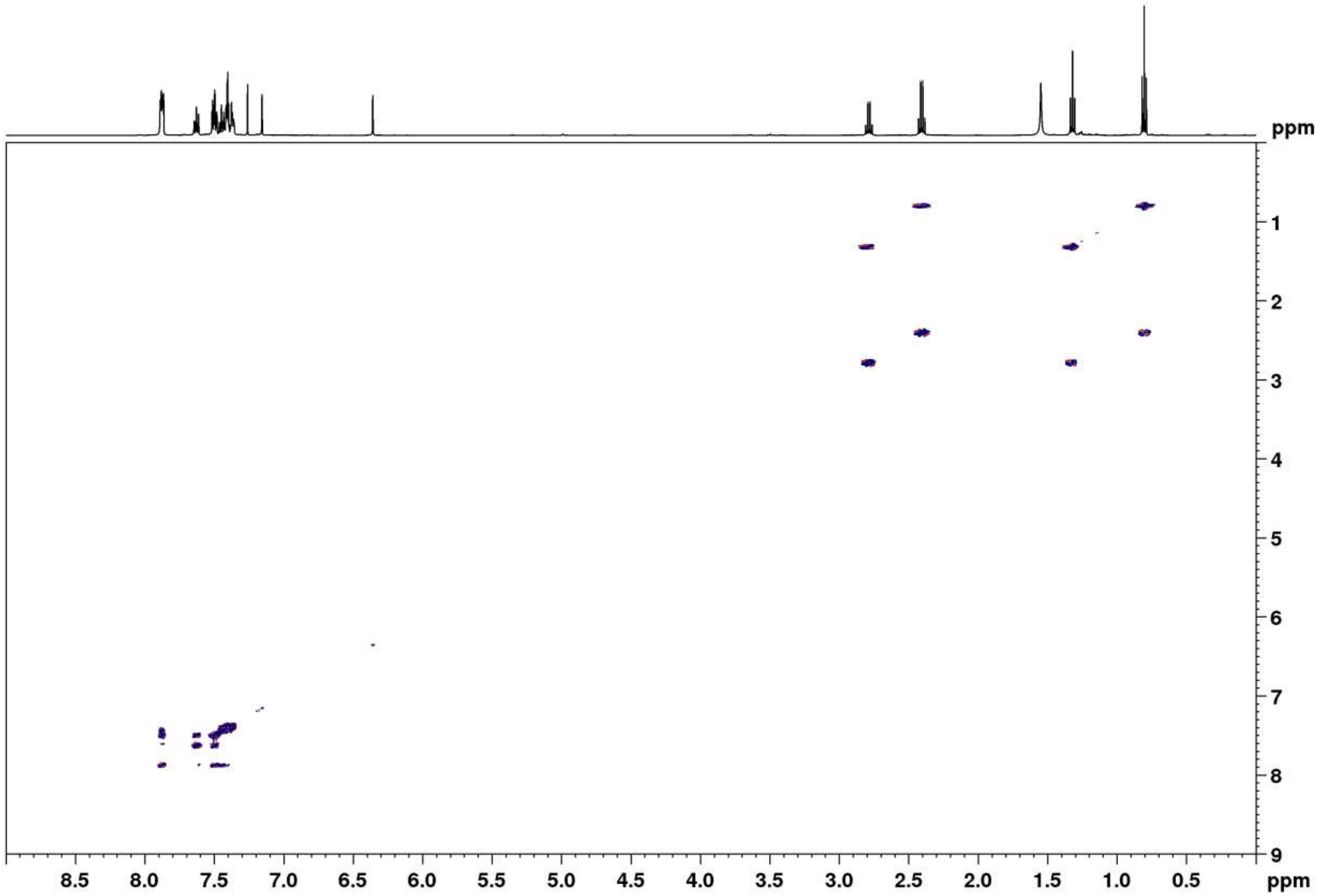


**Figure S34.** DQF-COSY spectrum of **3** and **4** (500 MHz, $CDCl_3$).

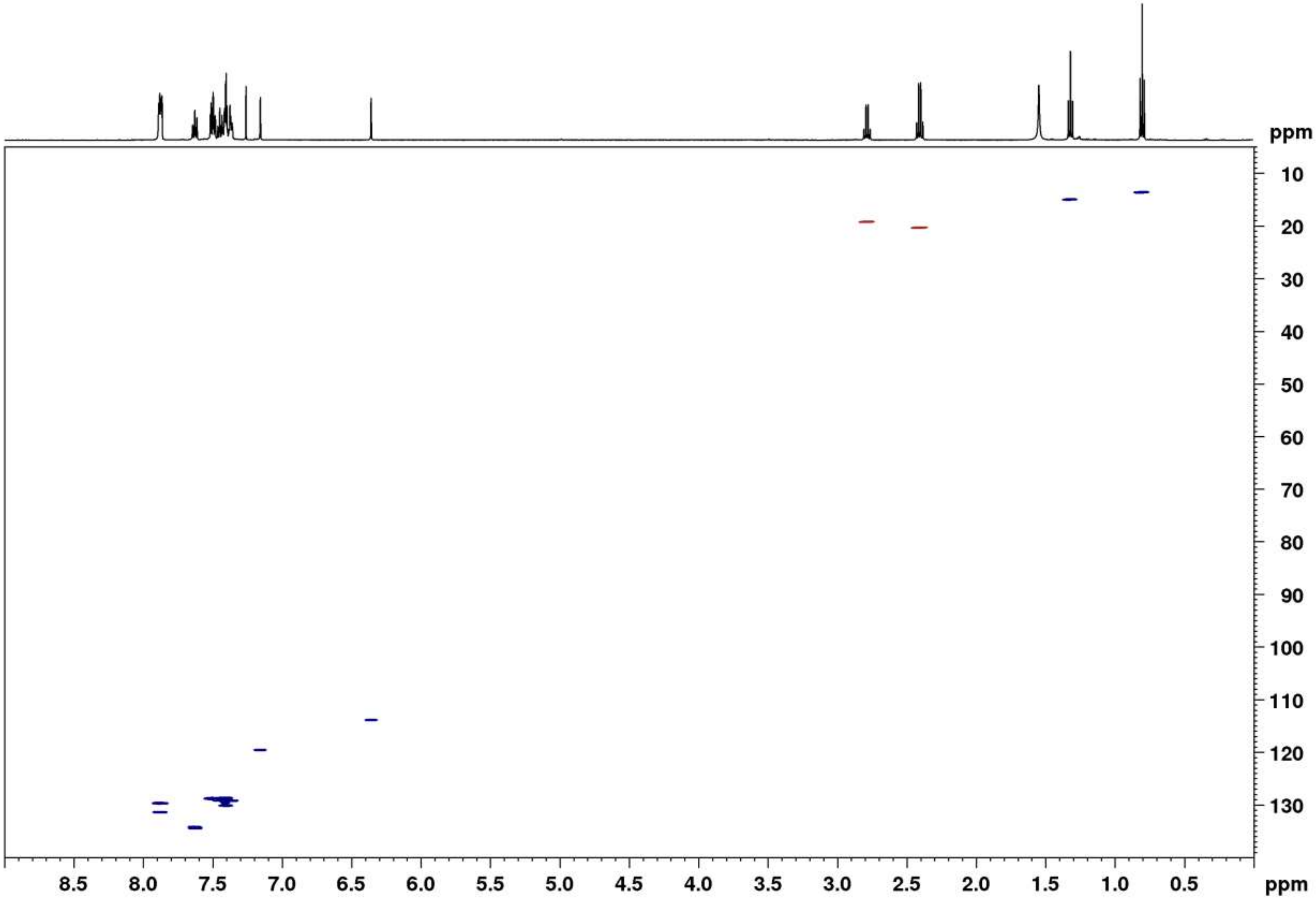


**Figure S35.** HSQC spectrum of **3** and **4** (500 MHz, $CDCl_3$).

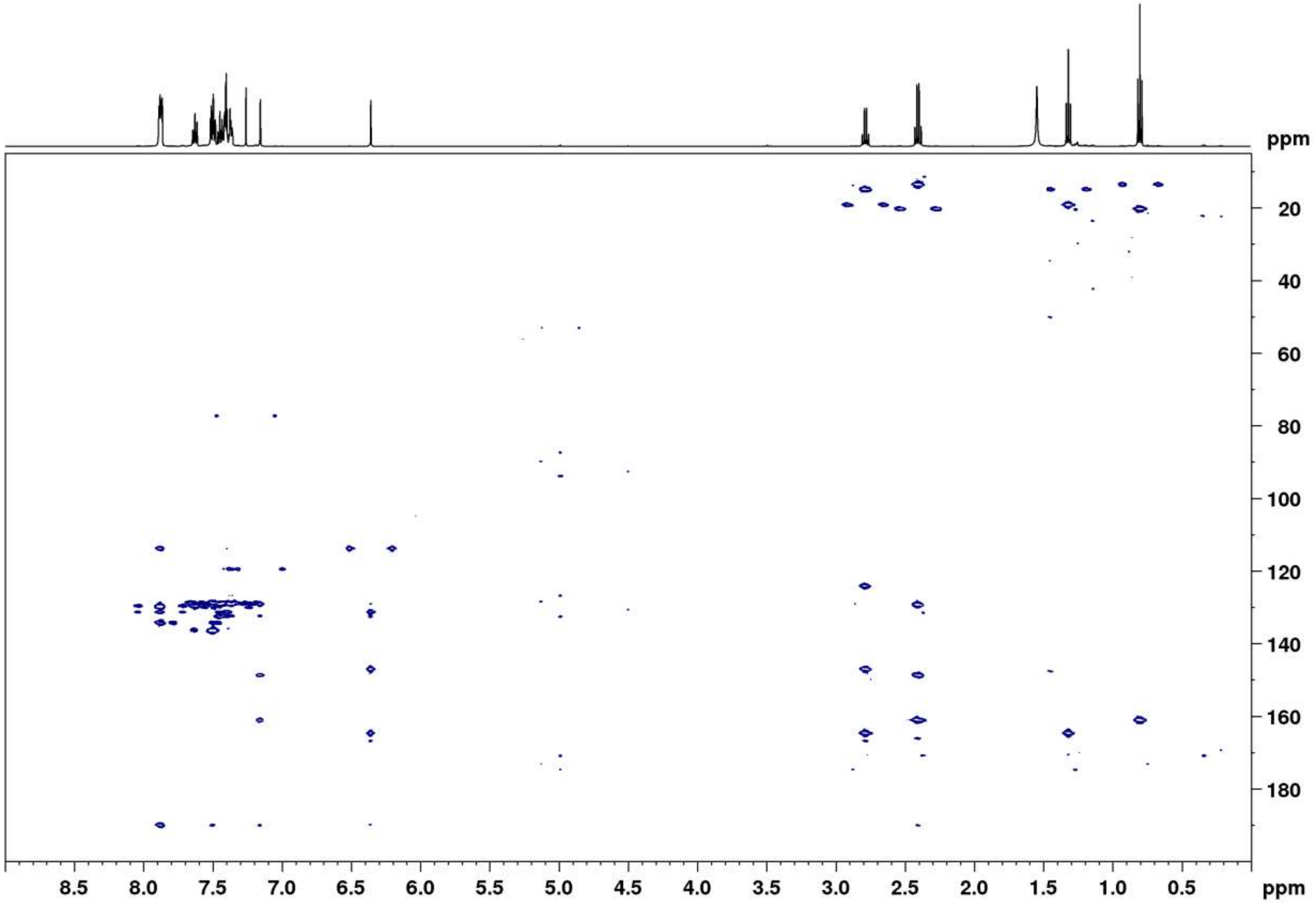


**Figure S36.** HMBC spectrum of **3** and **4** (500 MHz, $CDCl_3$).

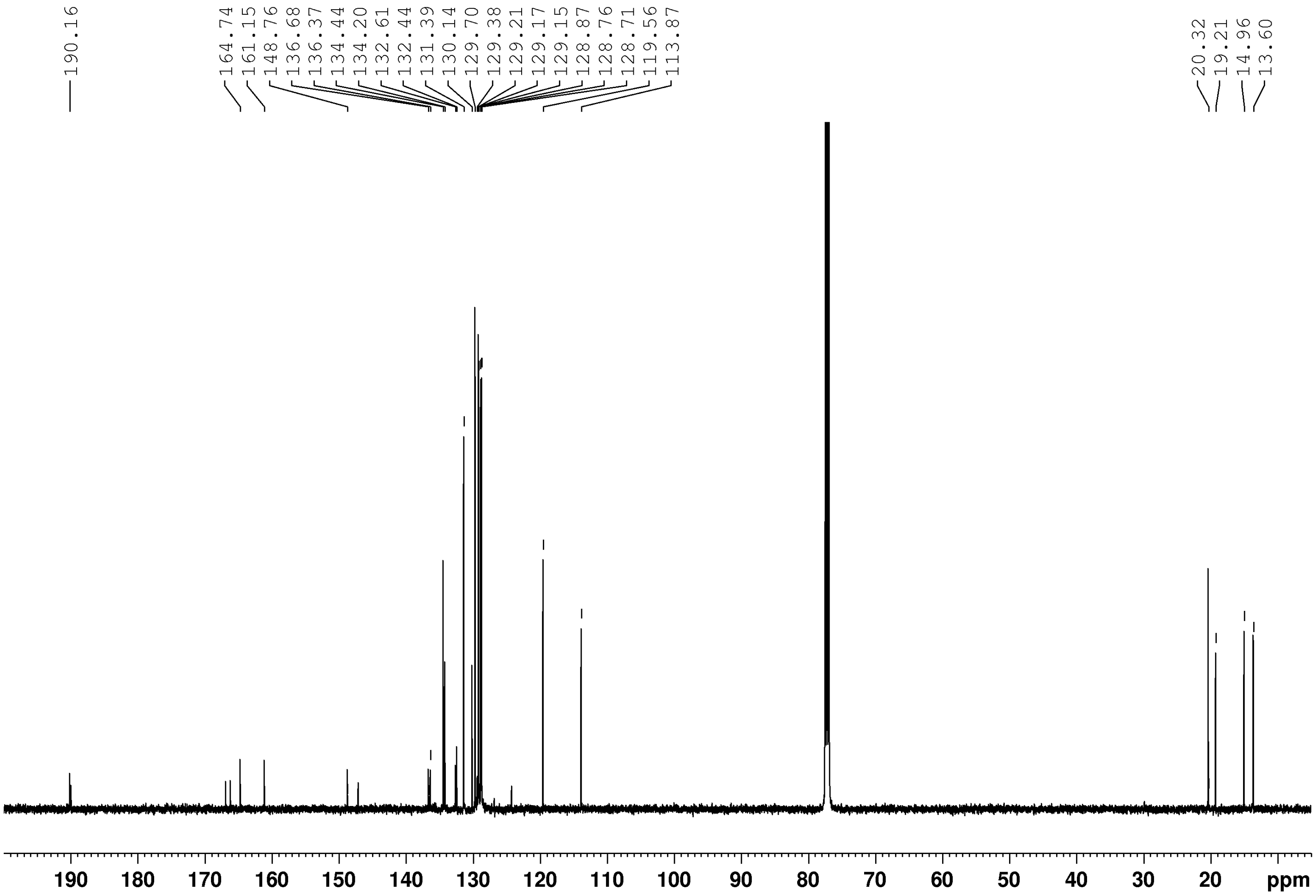


**Figure S37.** $^{13}C$ NMR spectrum of **3** and **4** (125 MHz, $CDCl_3$).

4.3 NMR spectra of **5**

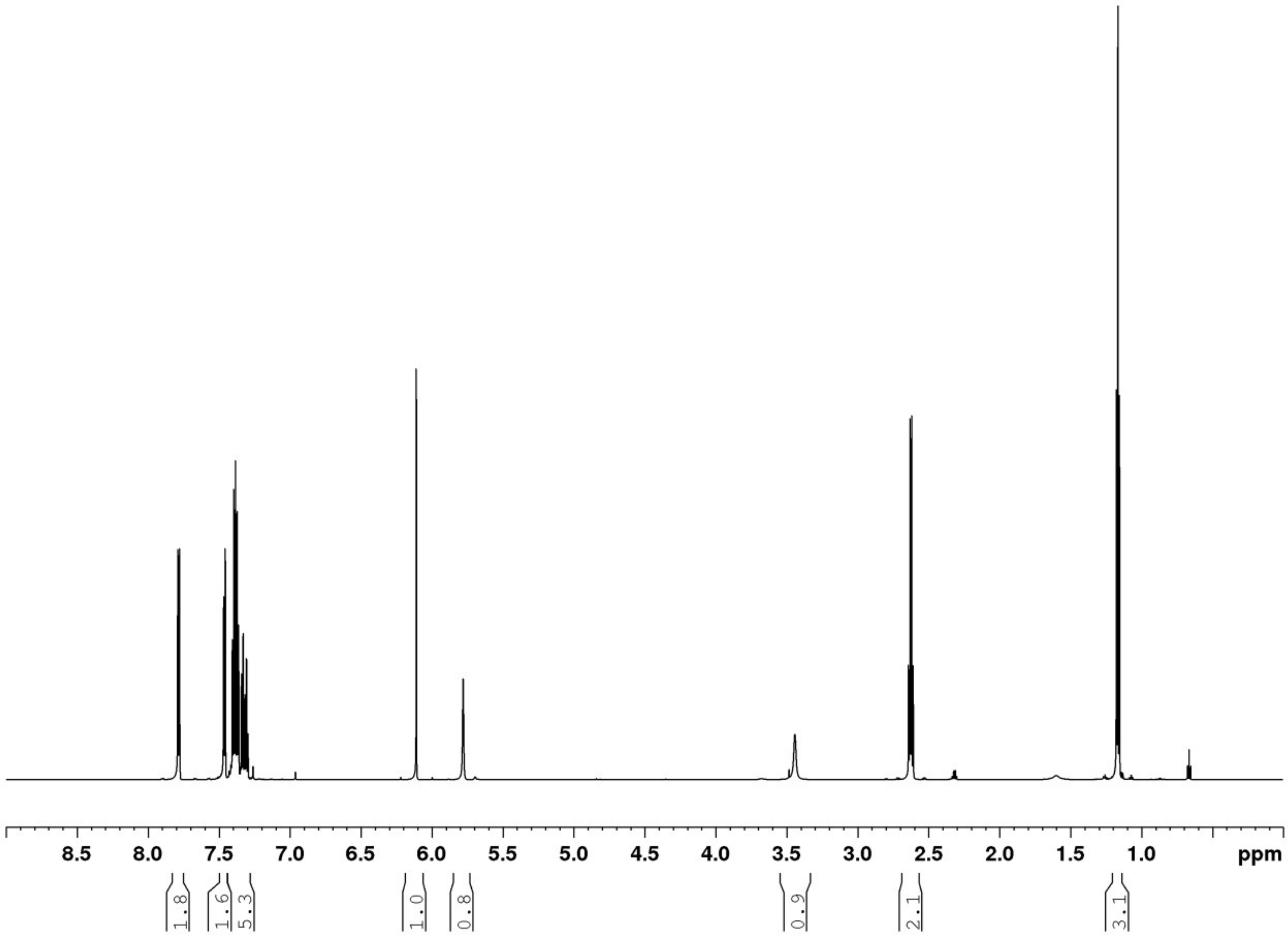


**Figure S38.** $^1$H NMR spectrum of **5** (700 MHz, $CDCl_3$).

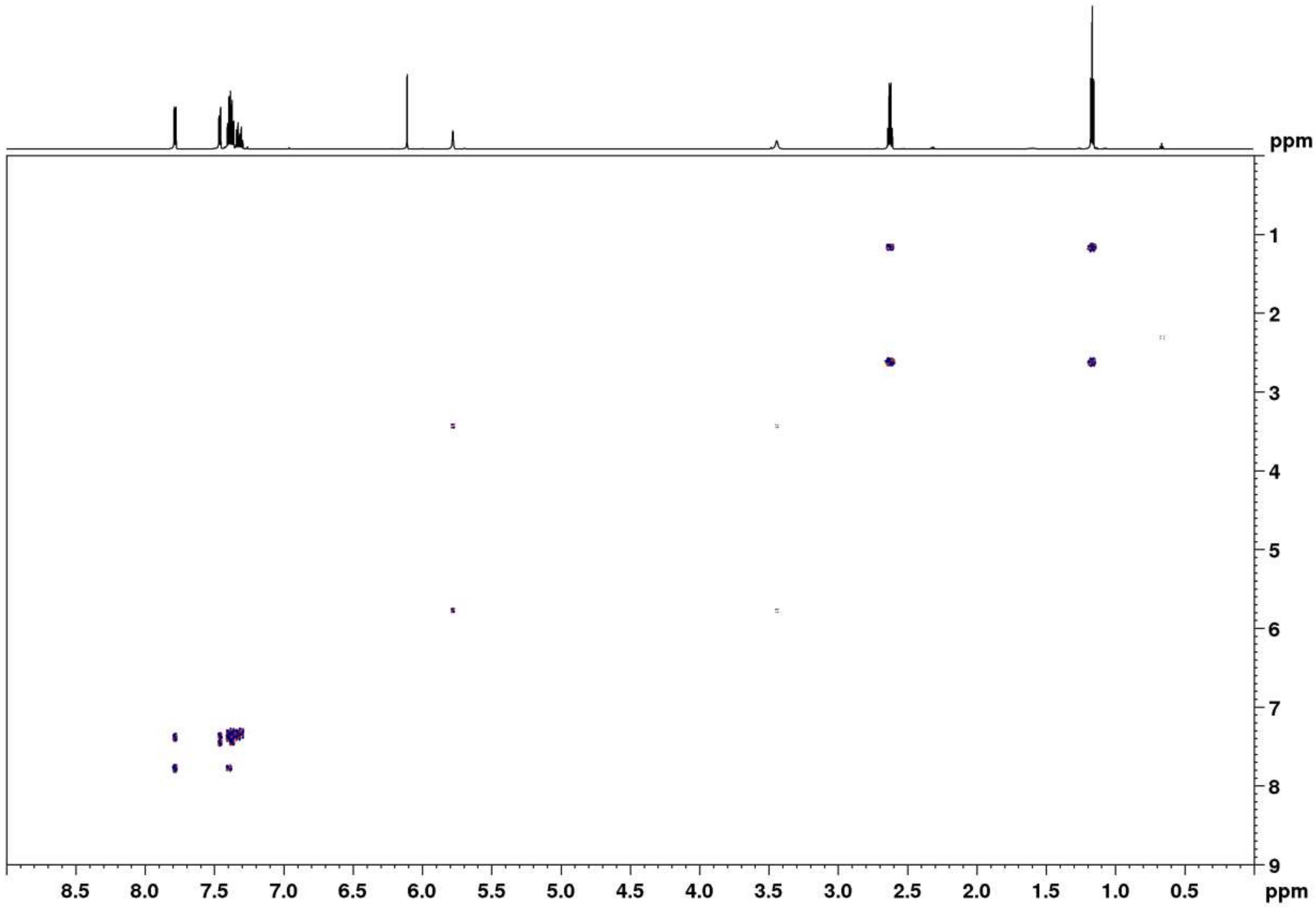


**Figure S39.** DQF-COSY spectrum of **5** (700 MHz, $CDCl_3$).

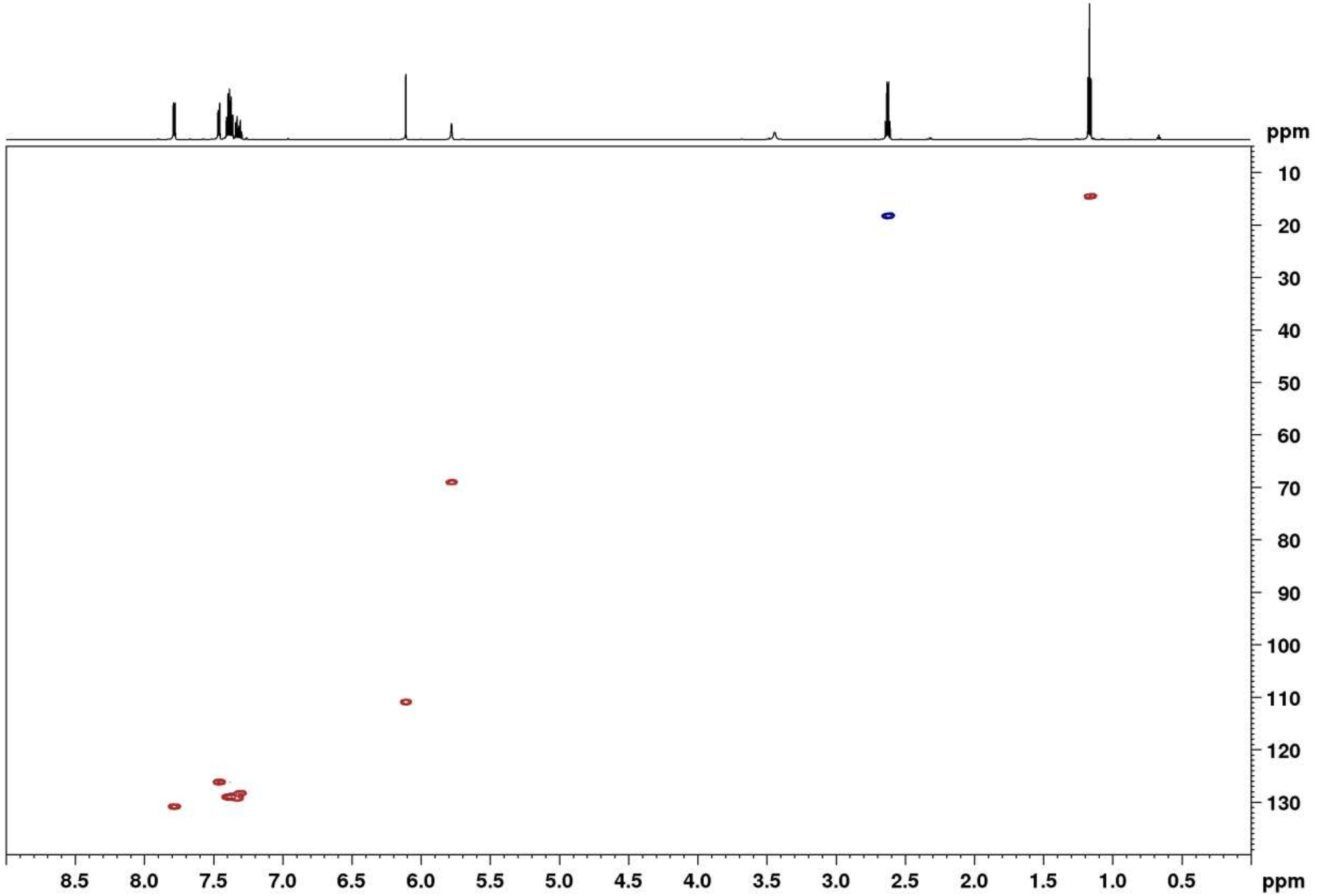


**Figure S40.** HSQC spectrum of **5** (700 MHz, $CDCl_3$).

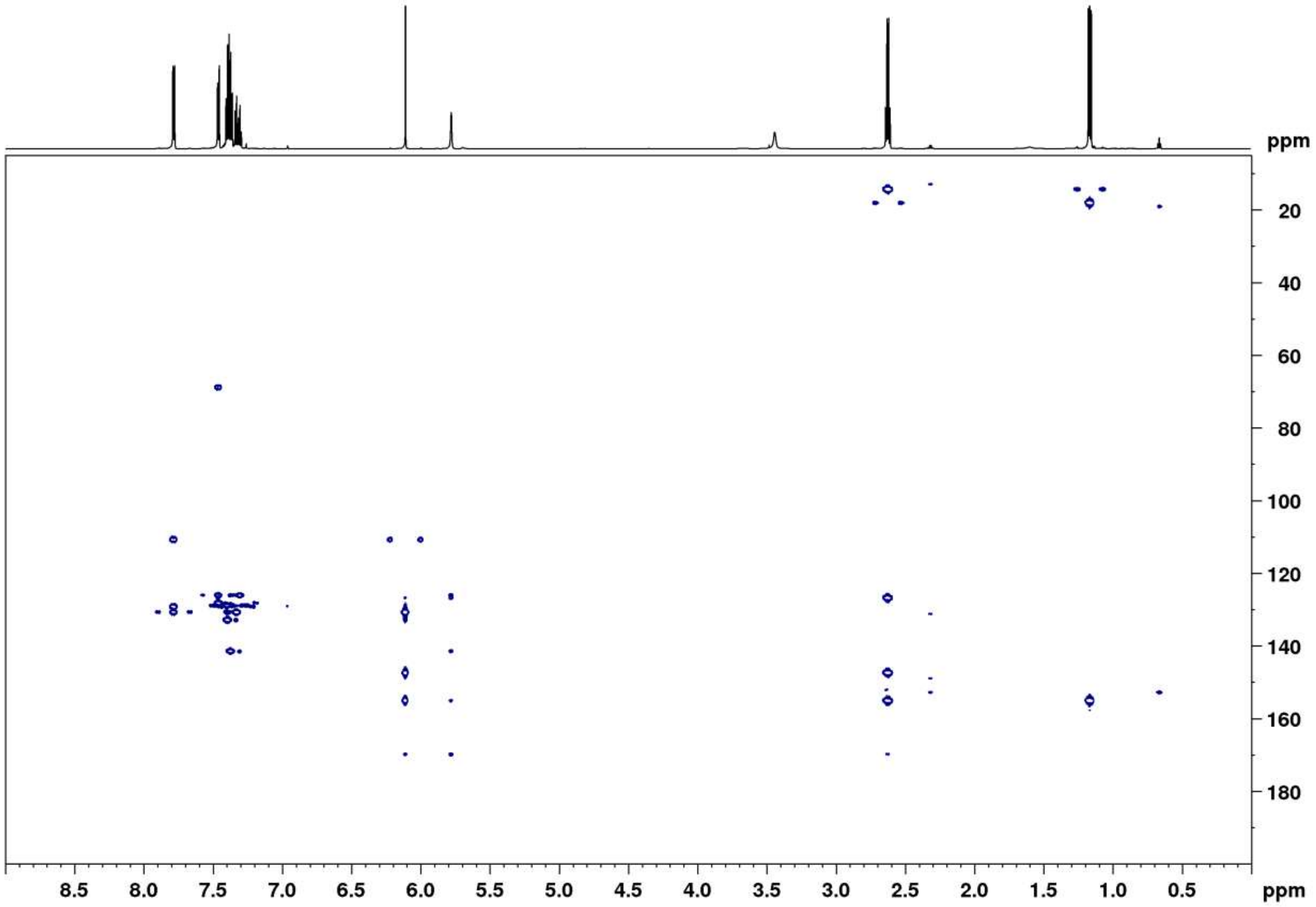


**Figure S41.** HMBC spectrum of **5** (700 MHz, $CDCl_3$).

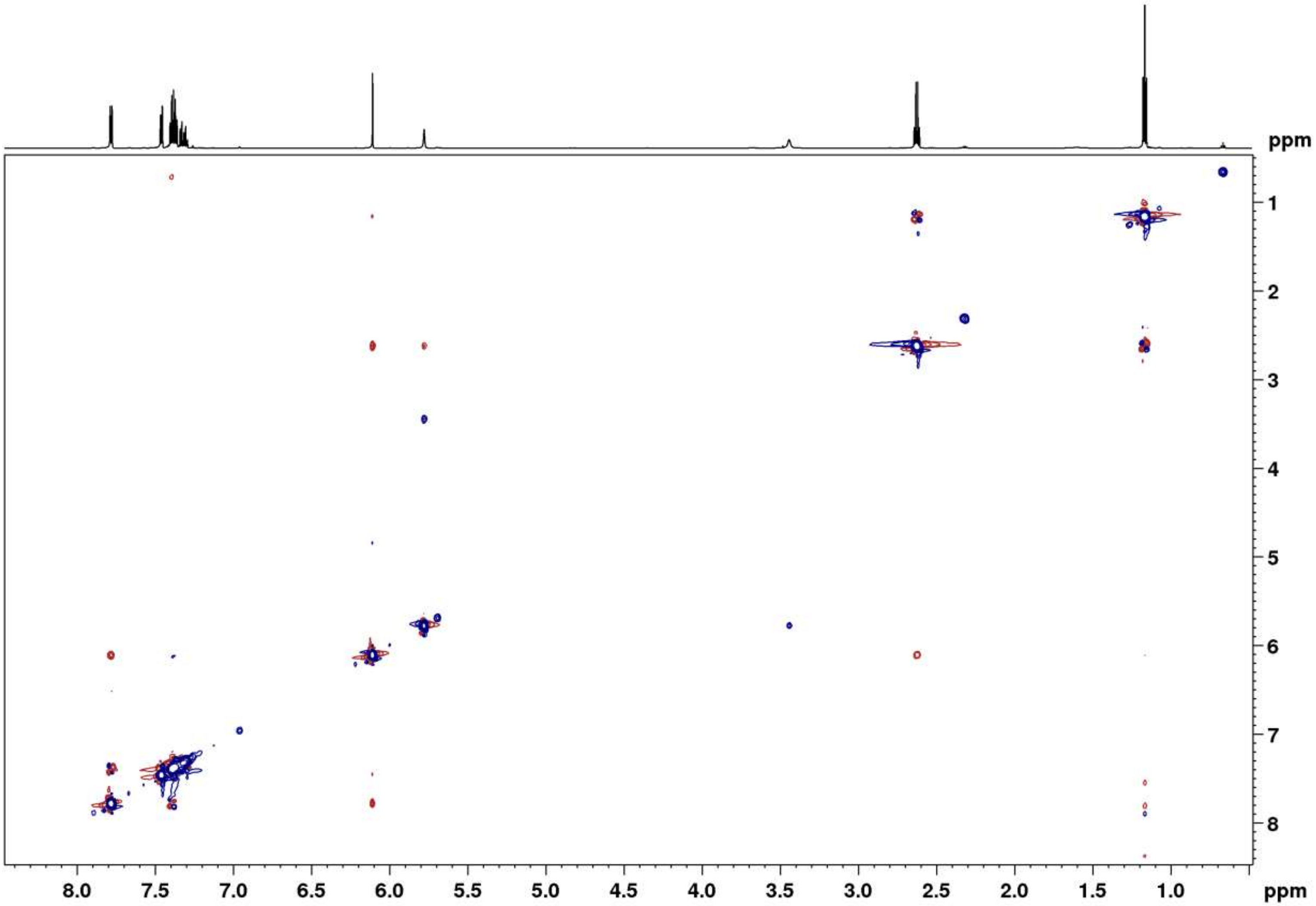


**Figure S42.** ROESY spectrum of **5** (700 MHz, $CDCl_3$, 400 ms).

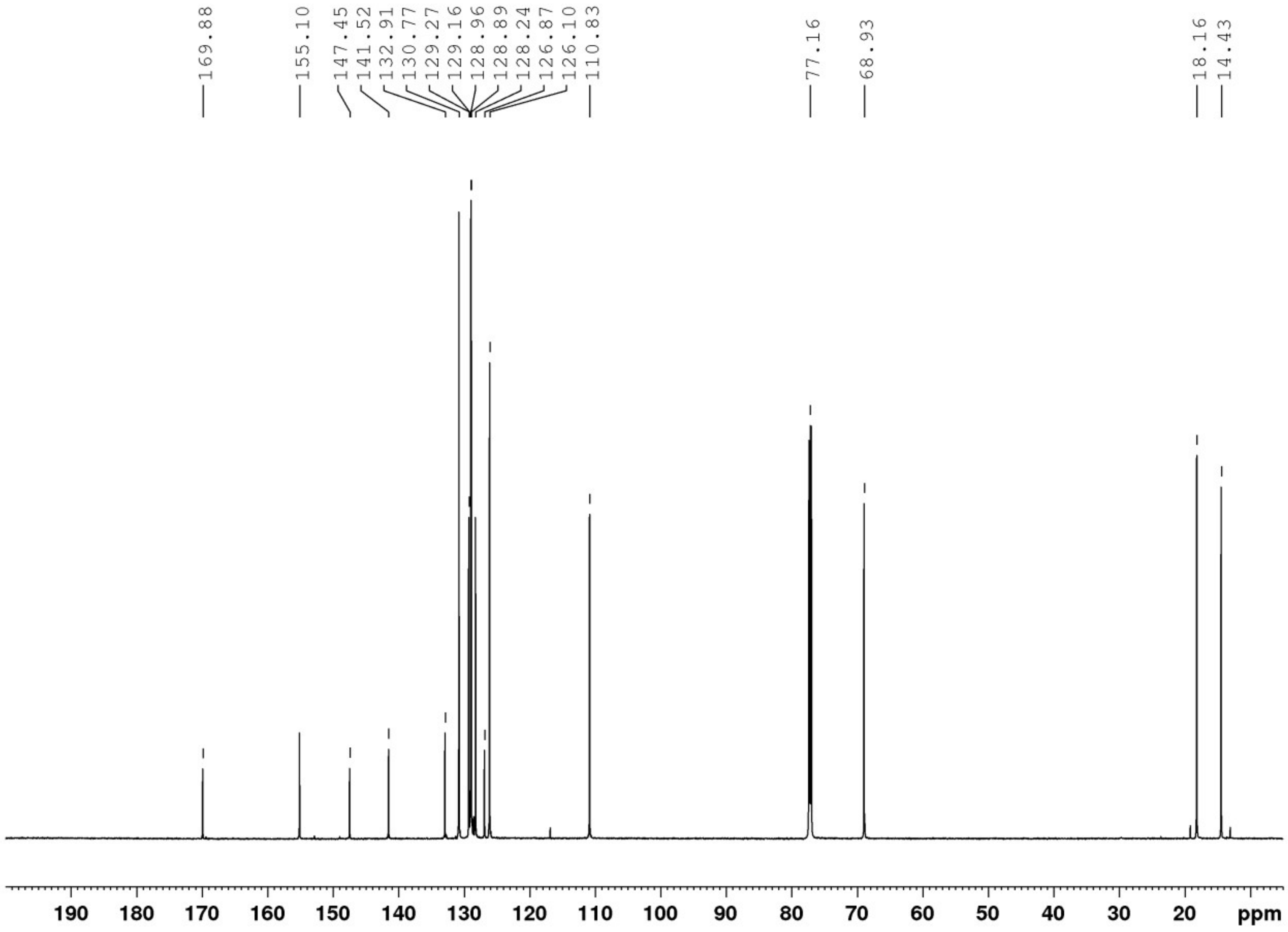


**Figure S43.** $^{13}C$ NMR spectrum of **5** (175 MHz, $CDCl_3$).

4.4 NMR spectra of **6**

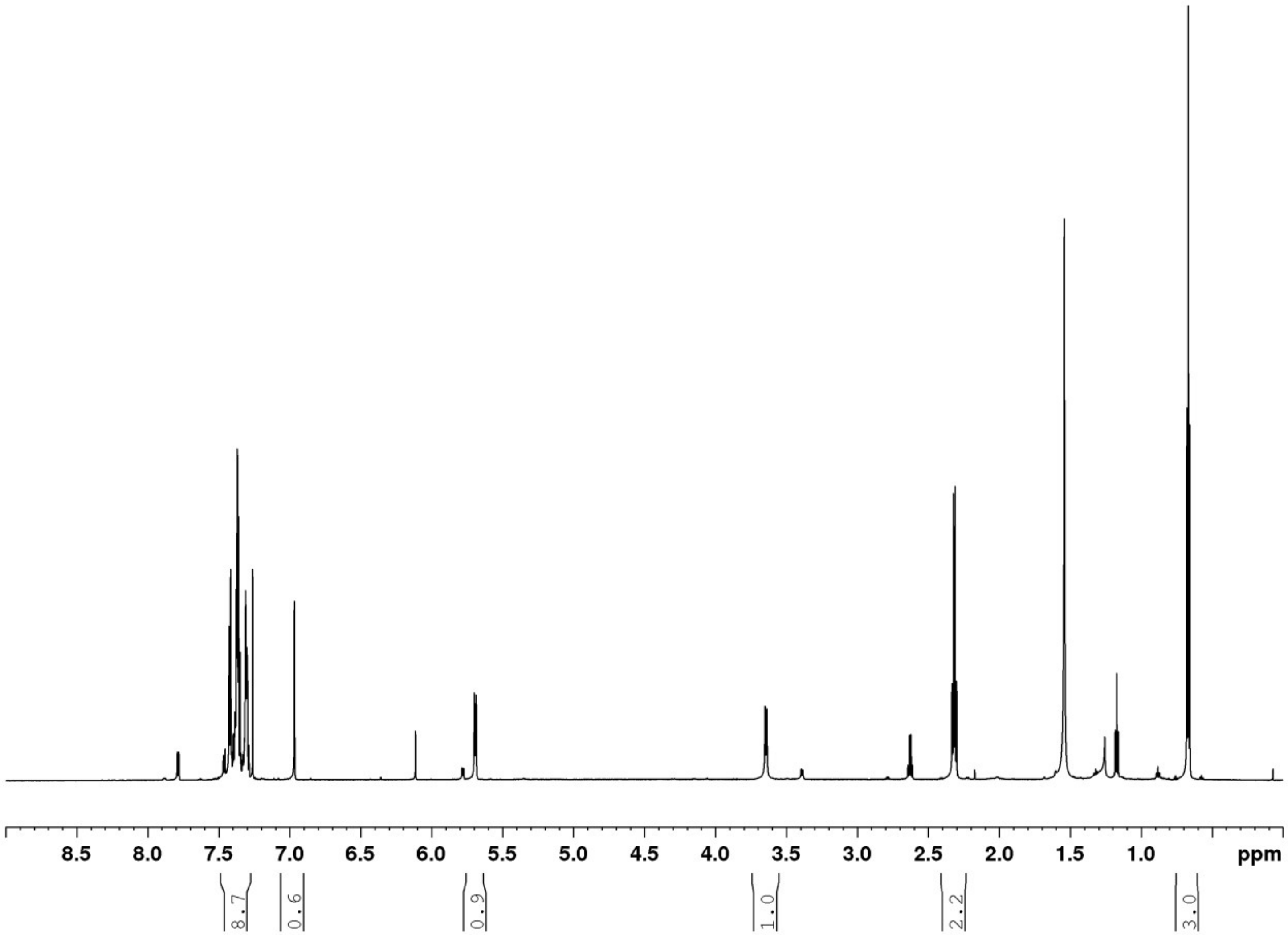


**Figure S44.** $^1$H NMR spectrum of **6** (700 MHz, $CDCl_3$).

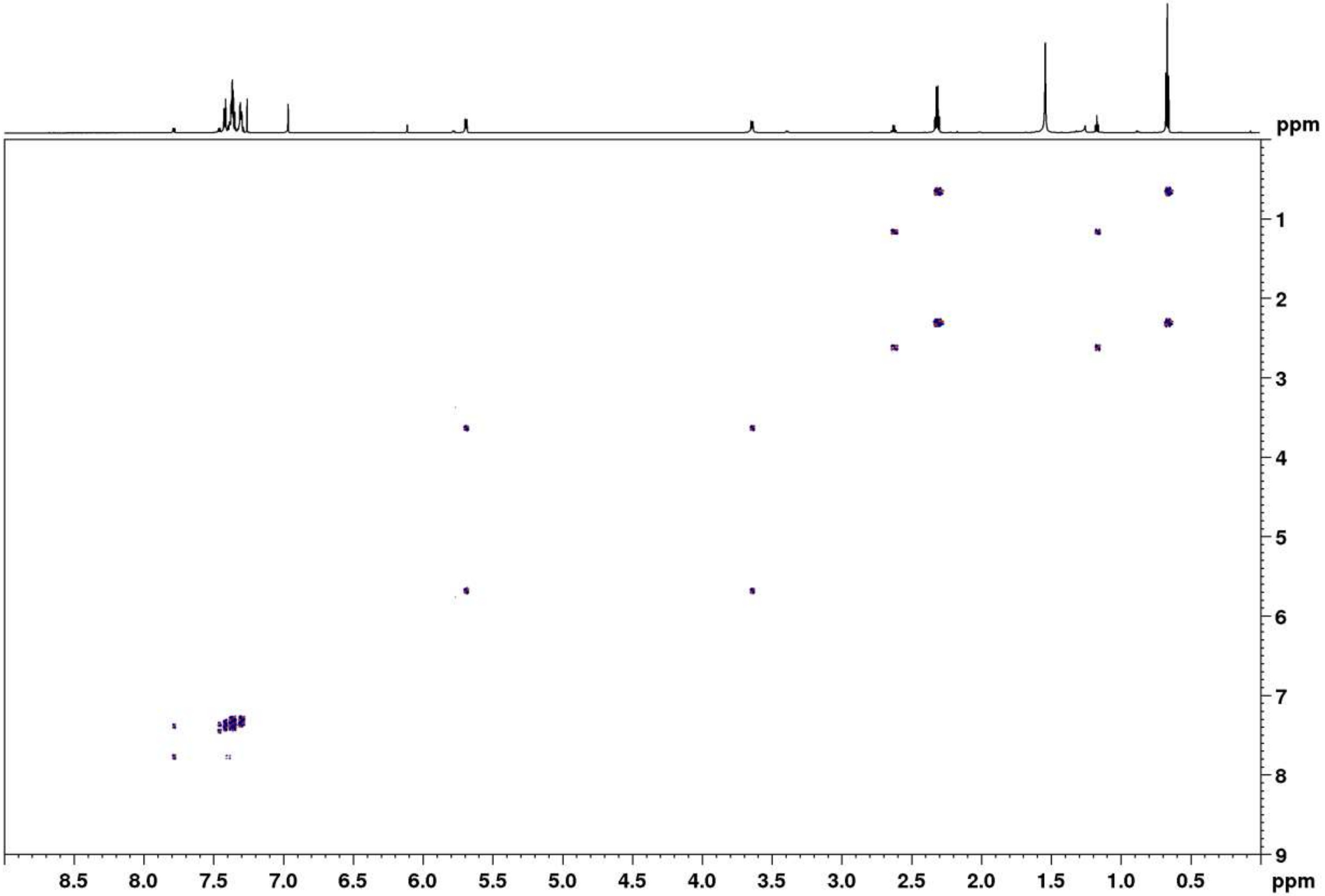


**Figure S45.** DQF-COSY spectrum of **6** (700 MHz, $CDCl_3$).

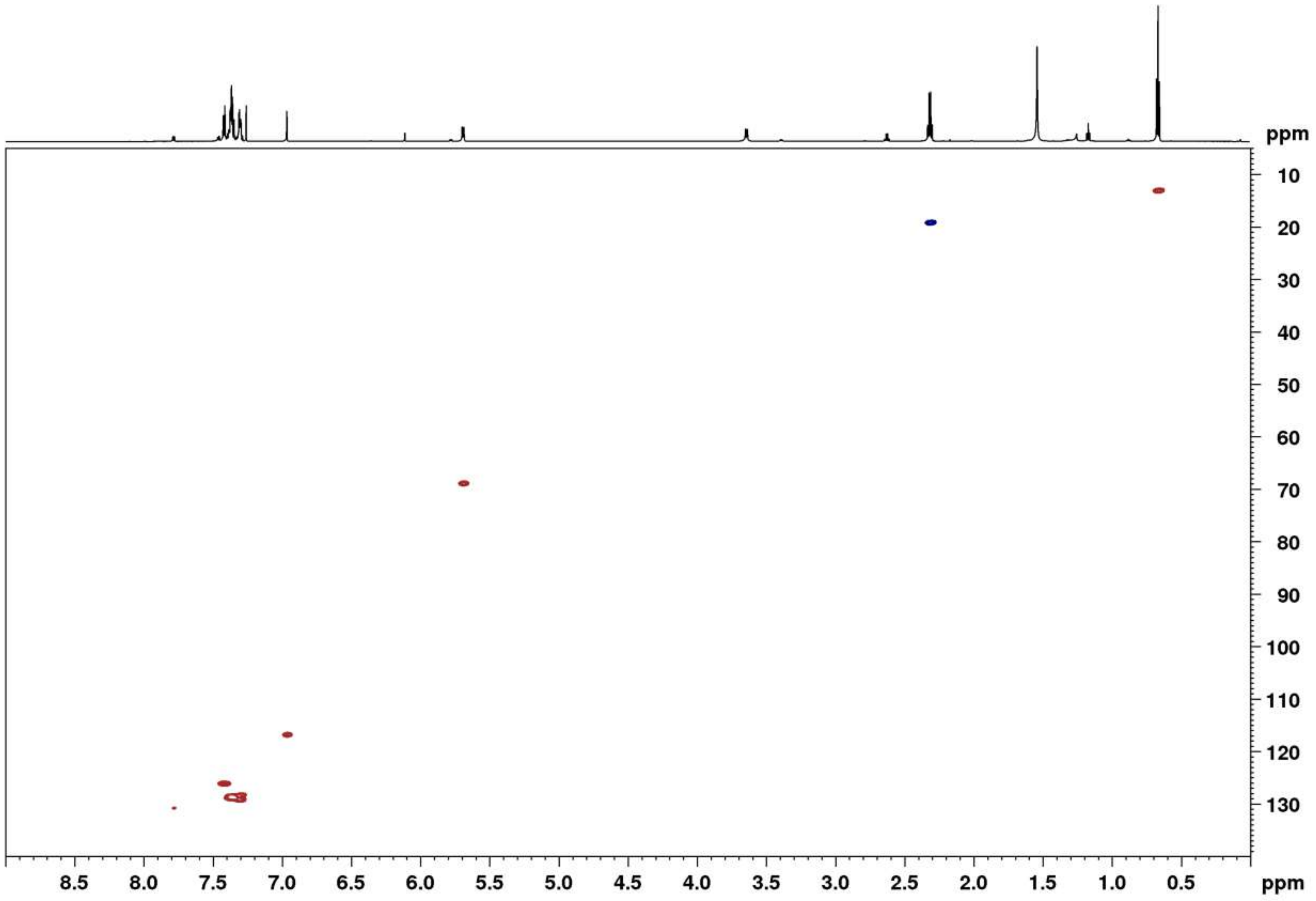


**Figure S46.** HSQC spectrum of **6** (700 MHz, $CDCl_3$).

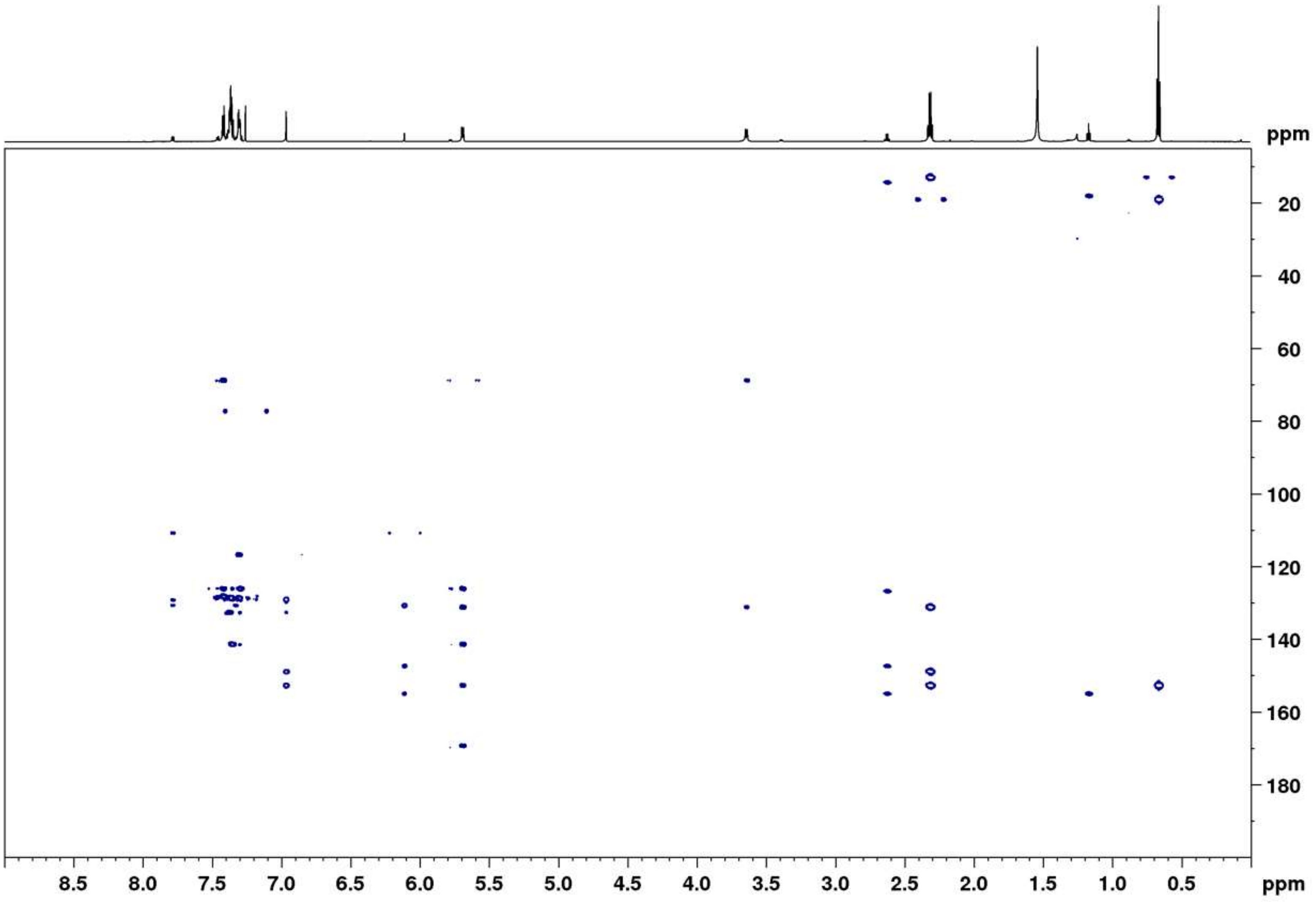


**Figure S47.** HMBC spectrum of **6** (700 MHz, $CDCl_3$).

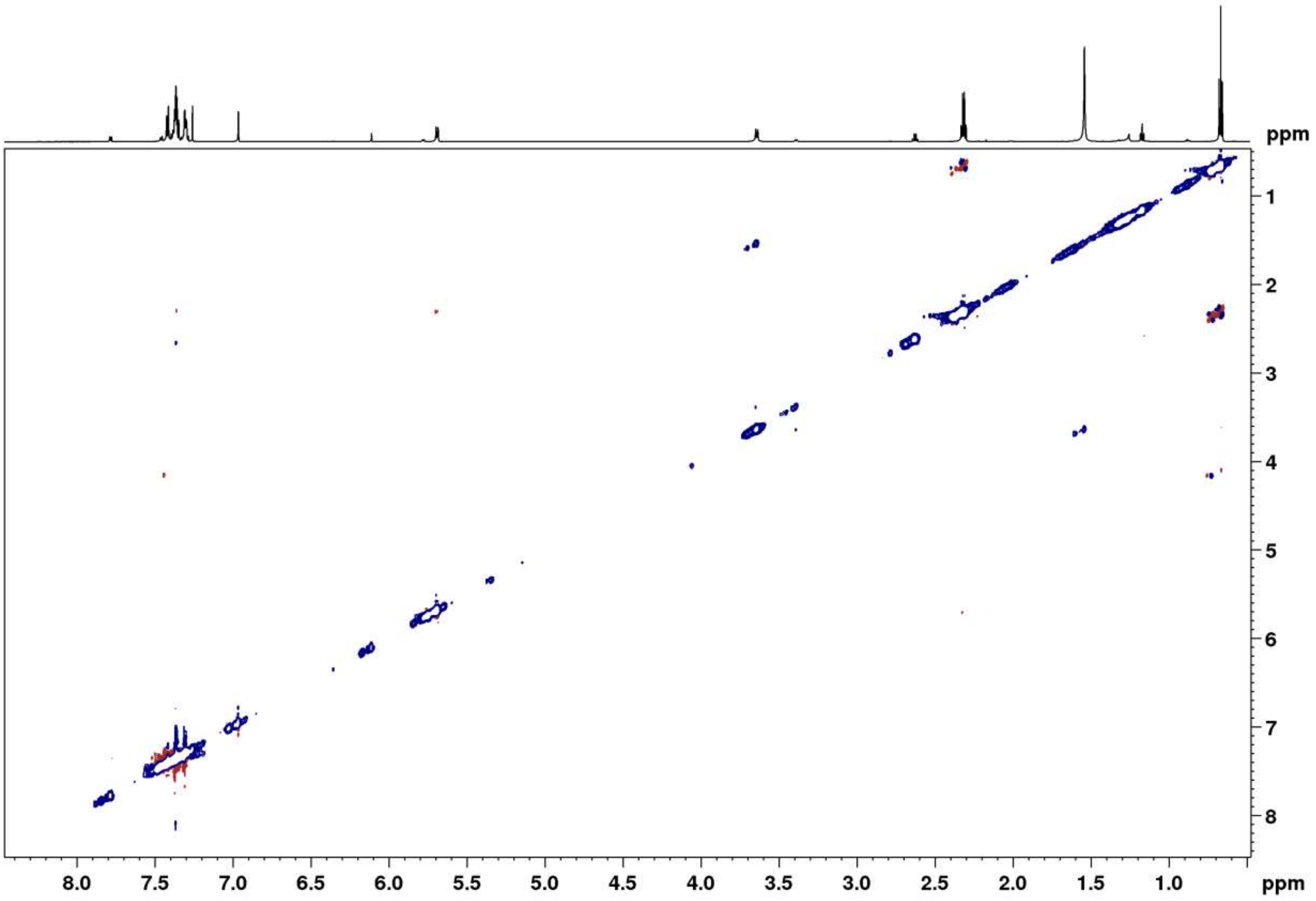


**Figure S48.** ROESY spectrum of **6** (700 MHz, $CDCl_3$, 300 ms).

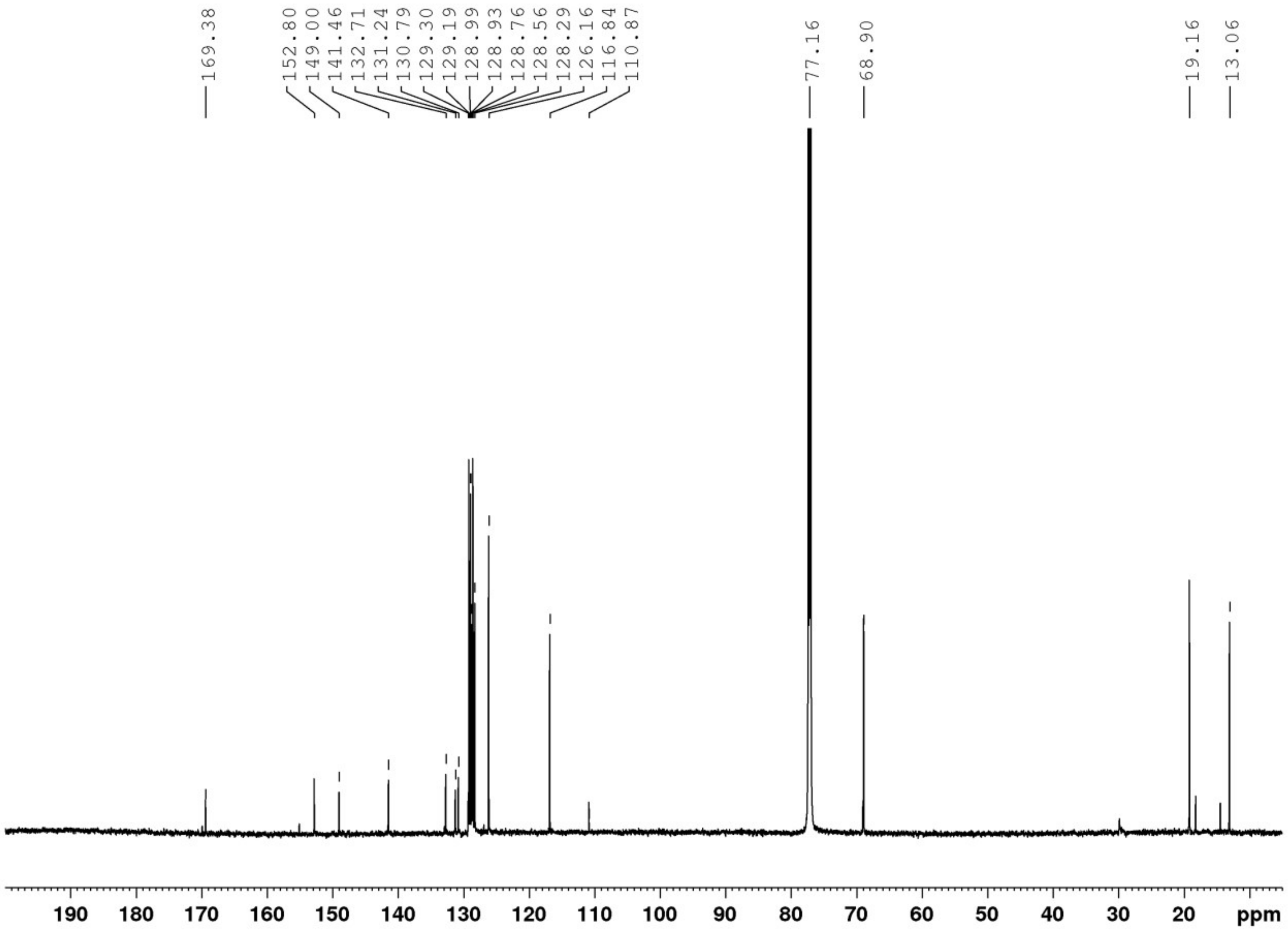


**Figure S49.** $^{13}C$ NMR spectrum of **6** (175 MHz, $CDCl_3$).

4.5 NMR spectra of **7**

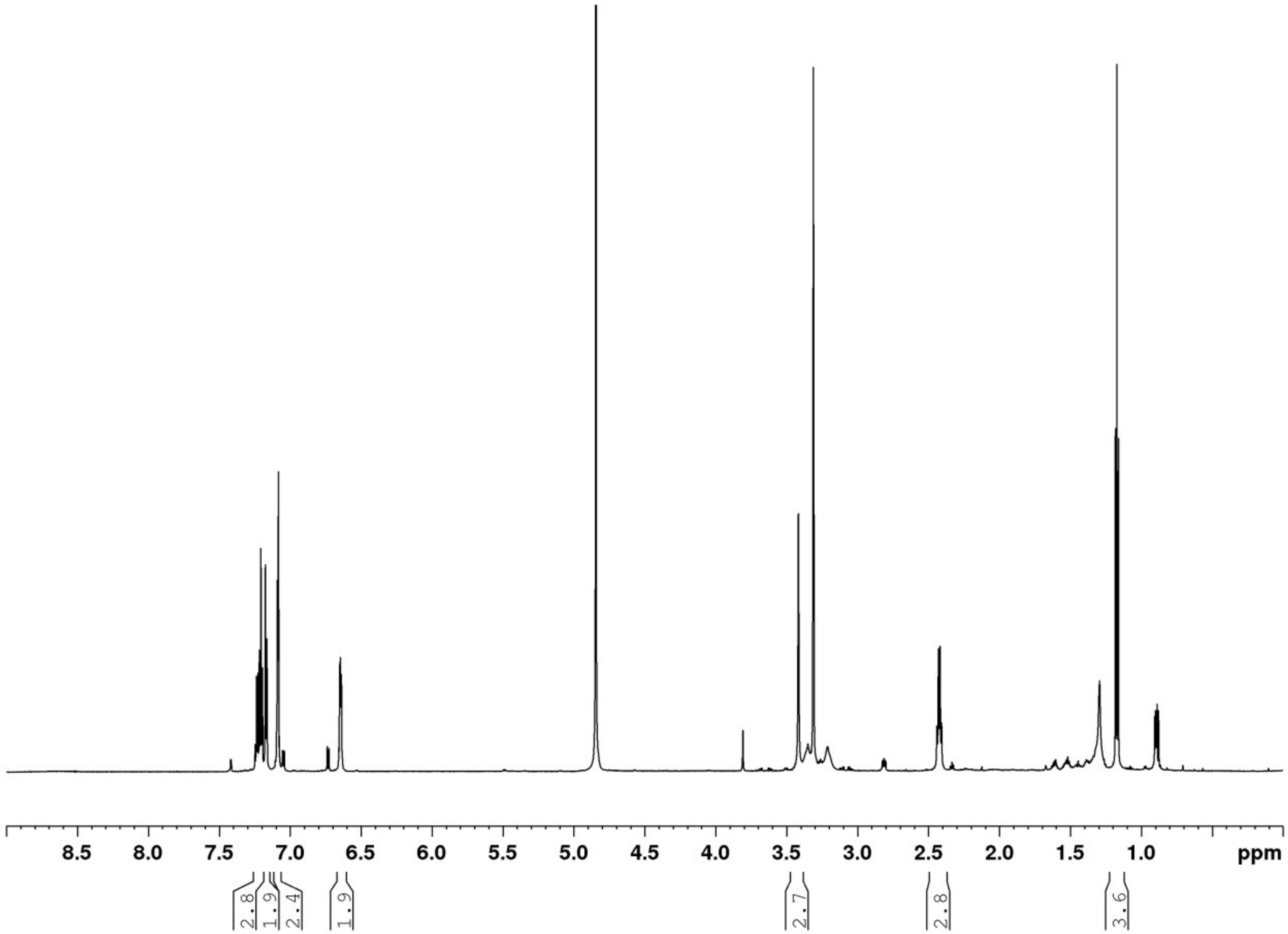


**Figure S50.** $^{1}$H NMR spectrum of **7** (700 MHz, $CD_3OD$).

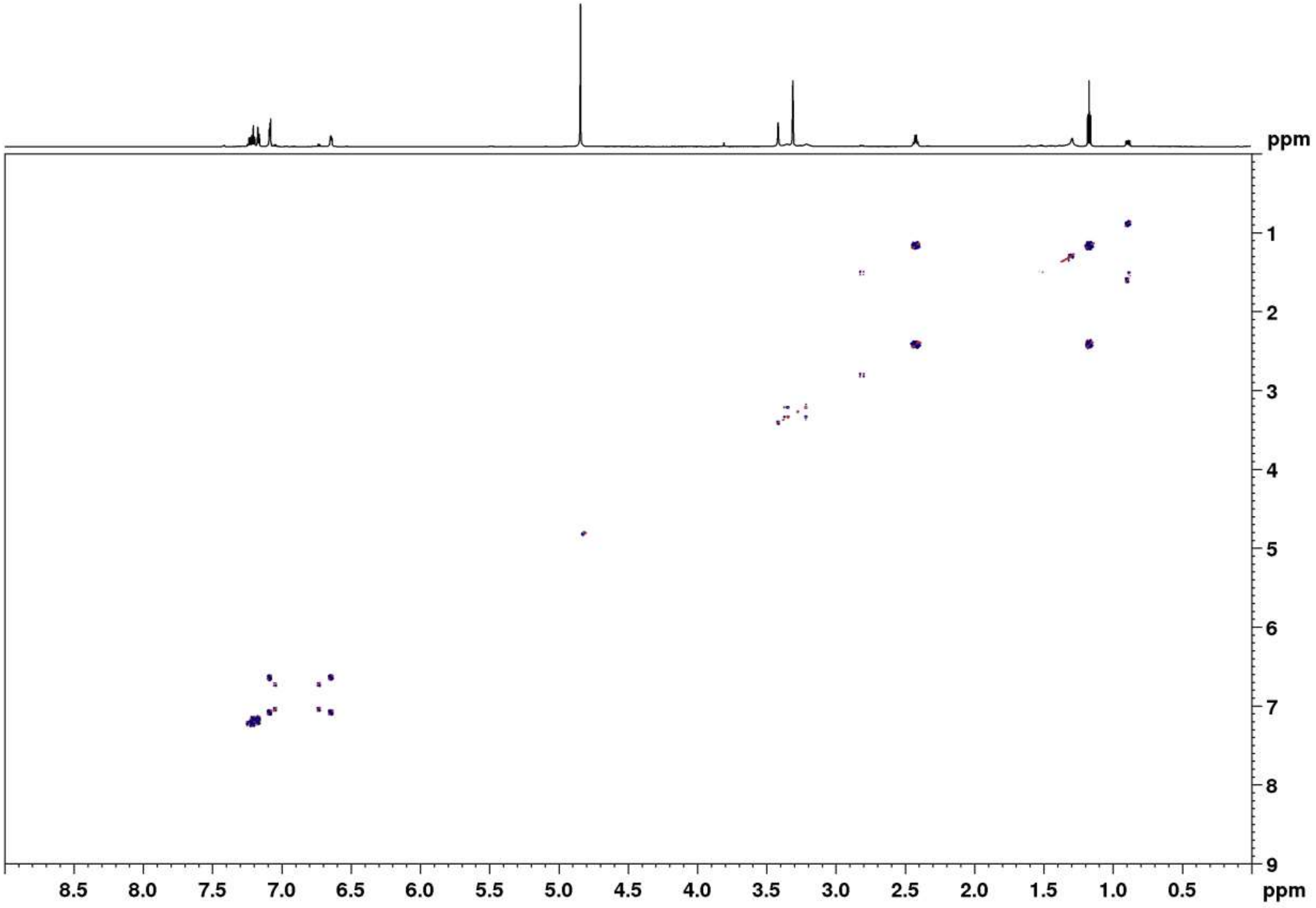


**Figure S51.** DQF-COSY spectrum of **7** (700 MHz, $CD_3OD$).

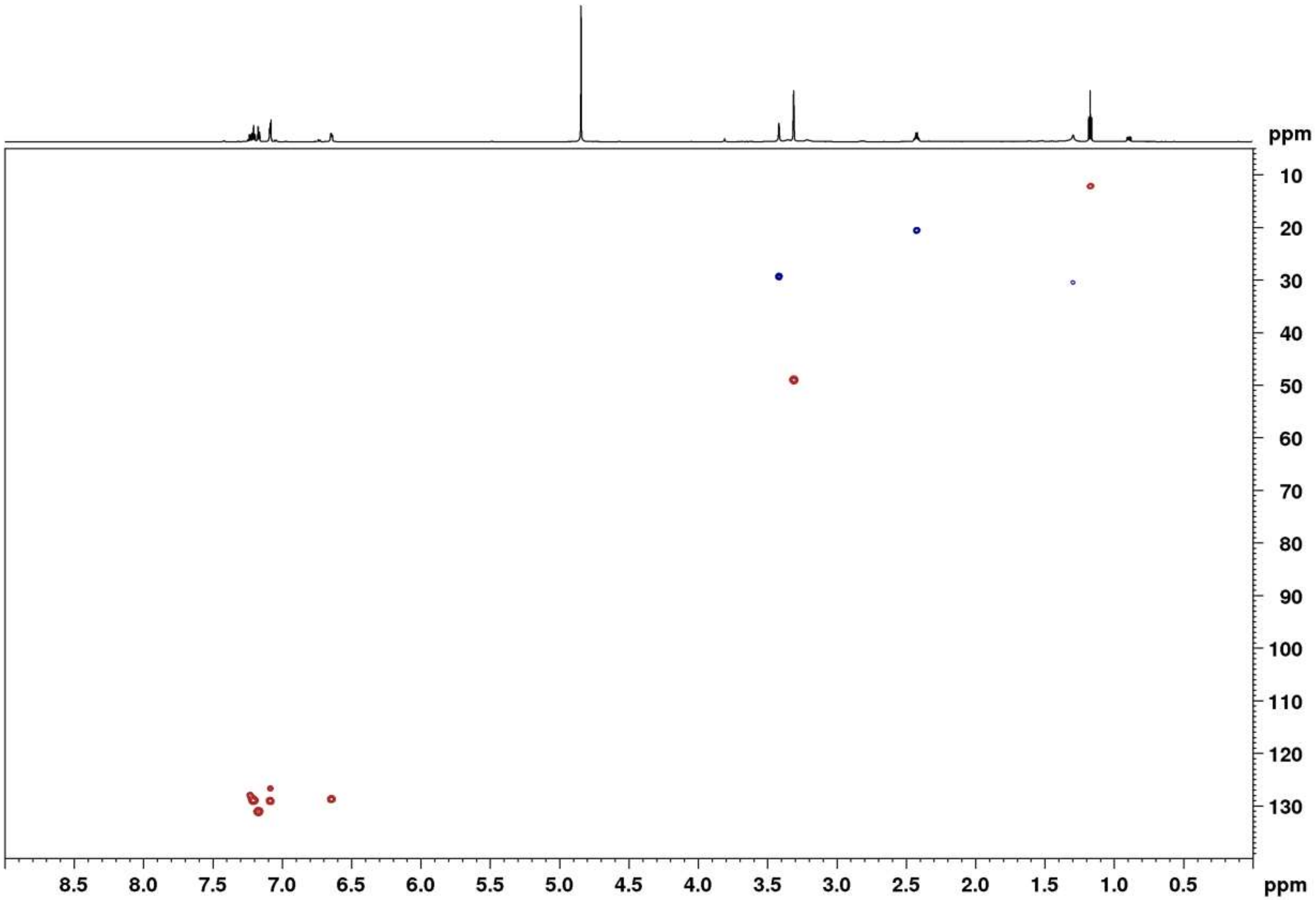


**Figure S52.** HSQC spectrum of **7** (700 MHz, $CD_3OD$).

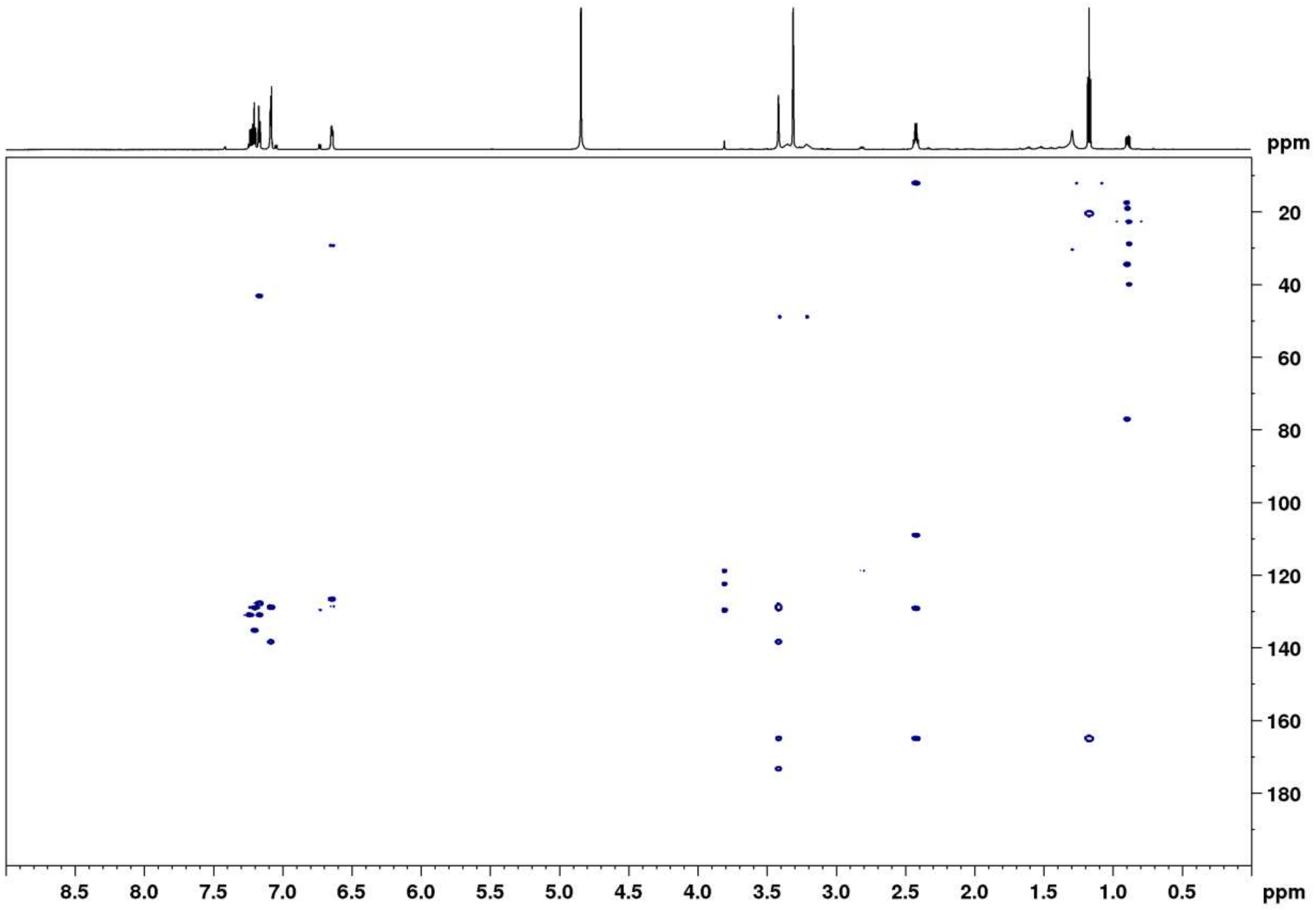


**Figure S53.** HMBC spectrum of **7** (700 MHz, $CD_3OD$).

4.6 NMR spectra of **8**

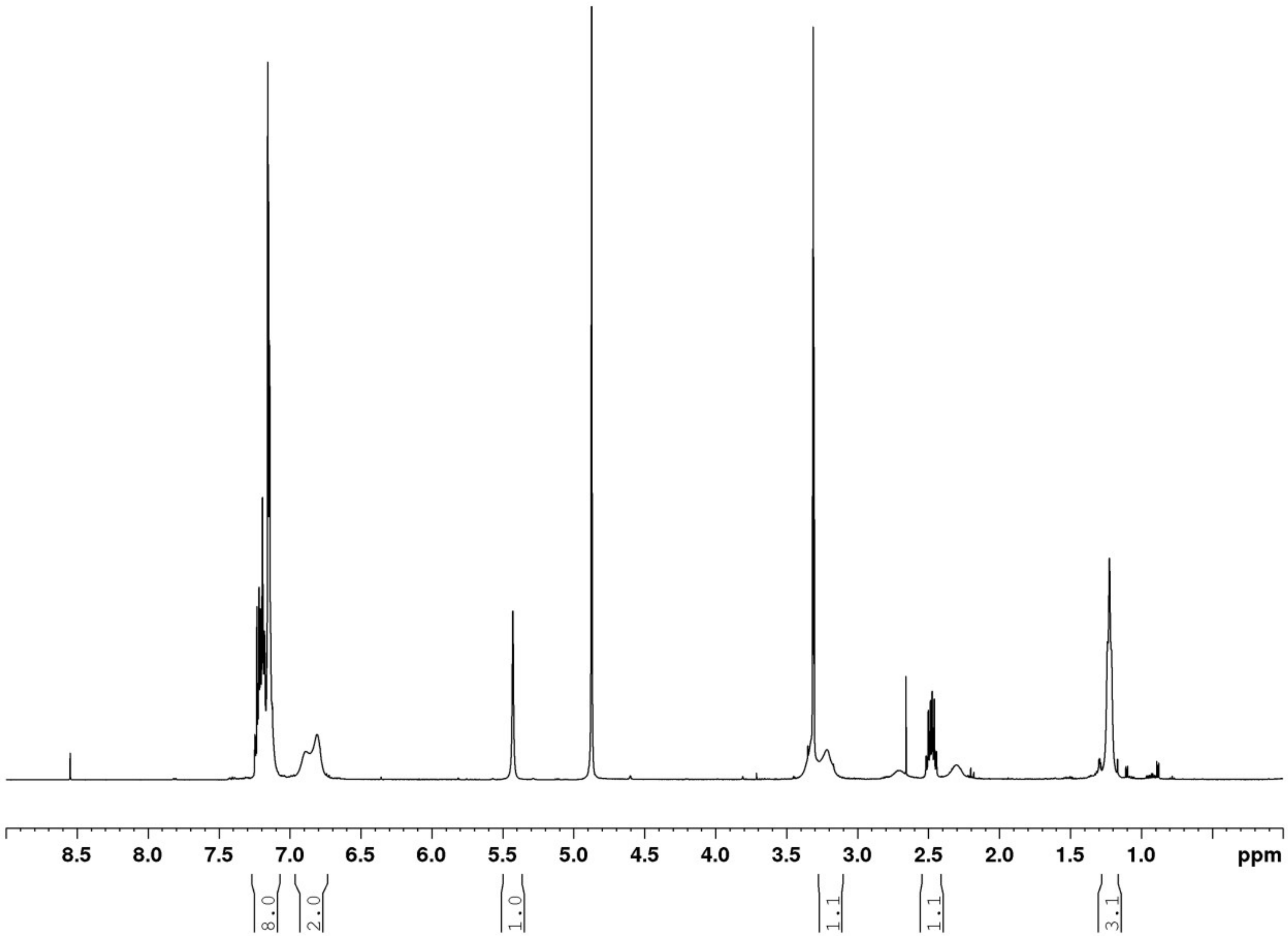


**Figure S54.** $^{1}H$ NMR spectrum of **8** (500 MHz, $CD_3OD$).

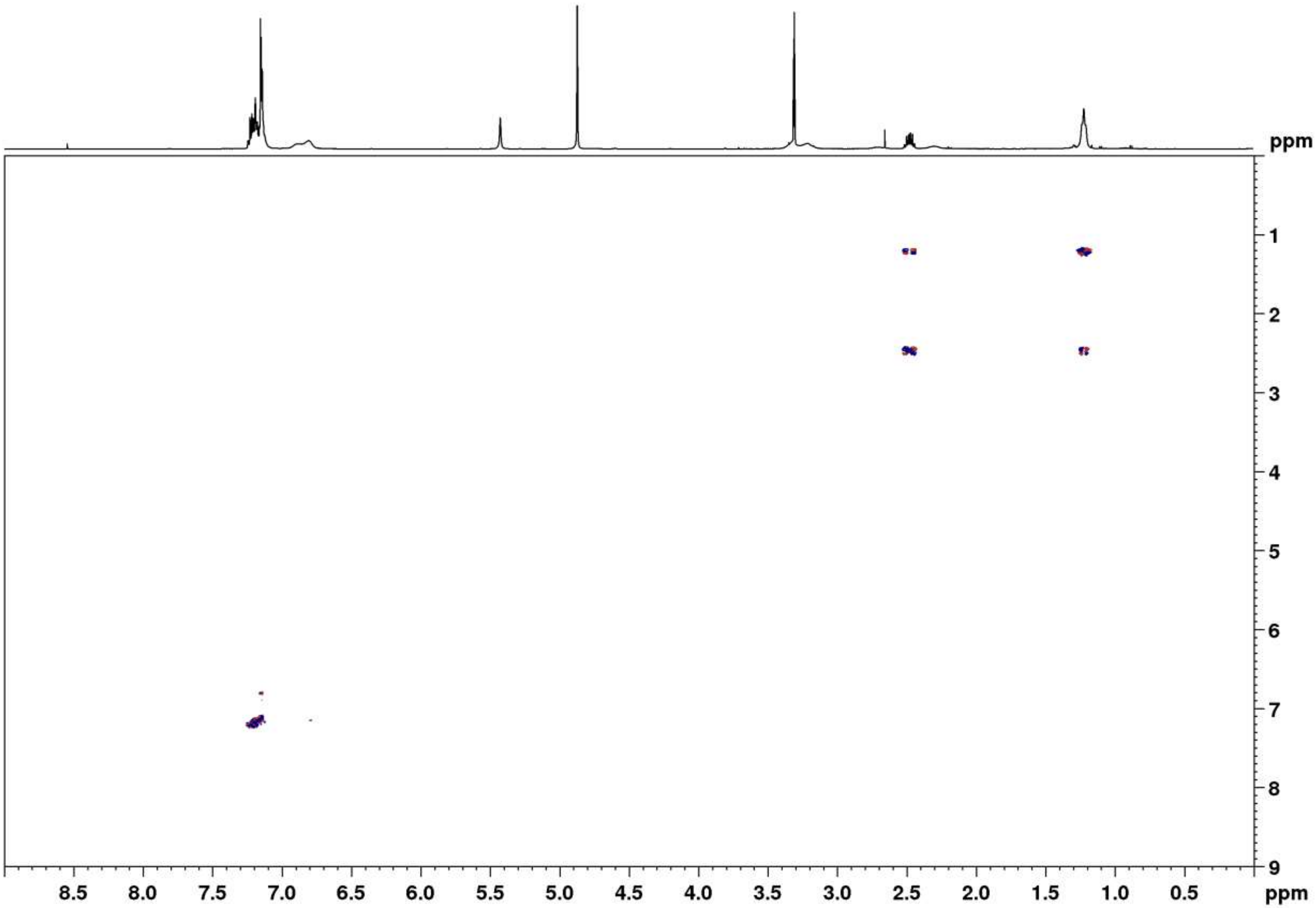


**Figure S55.** DQF-COSY spectrum of **8** (500 MHz, $CD_3OD$).

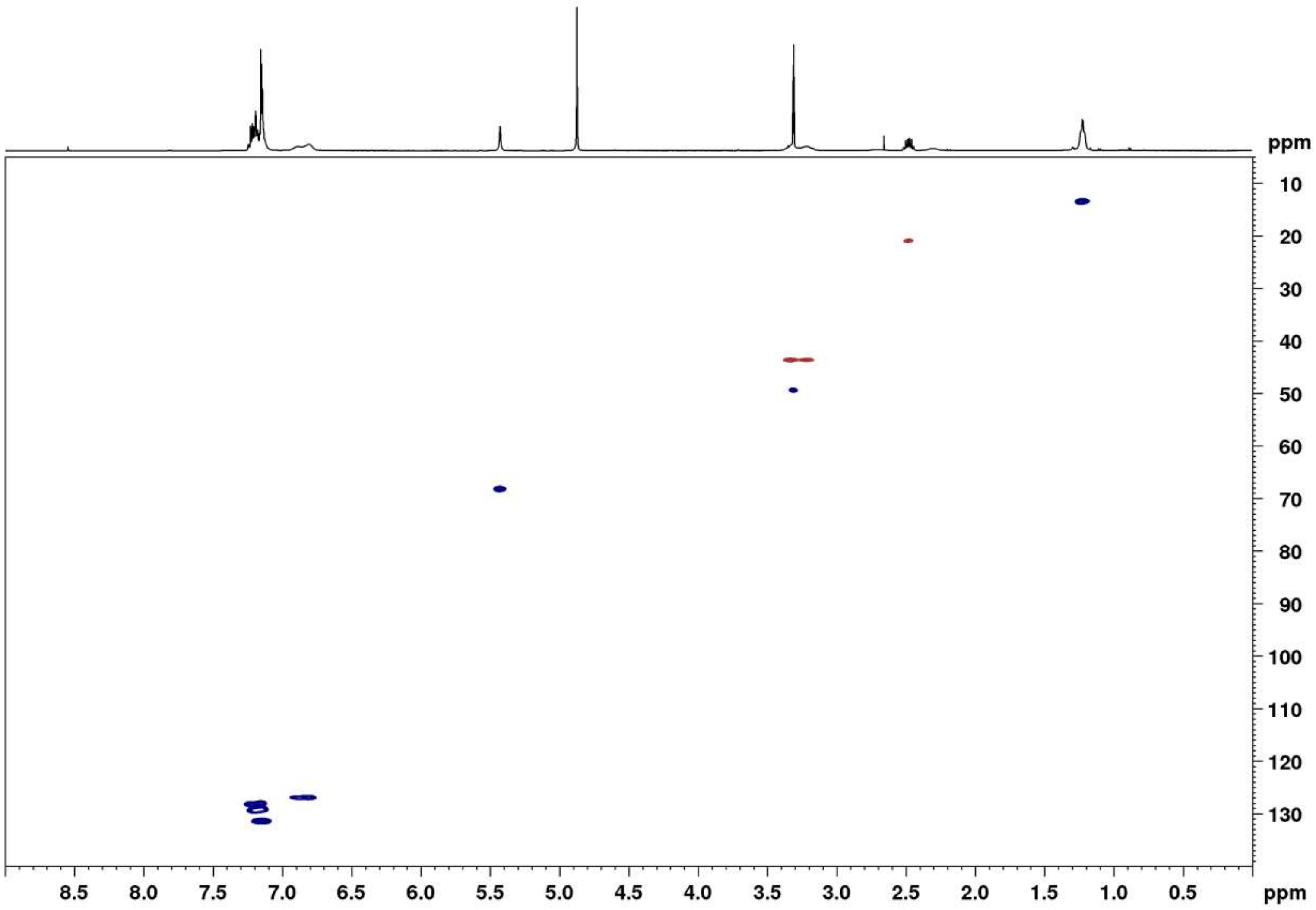


**Figure S56.** HSQC spectrum of **8** (500 MHz, $CD_3OD$).

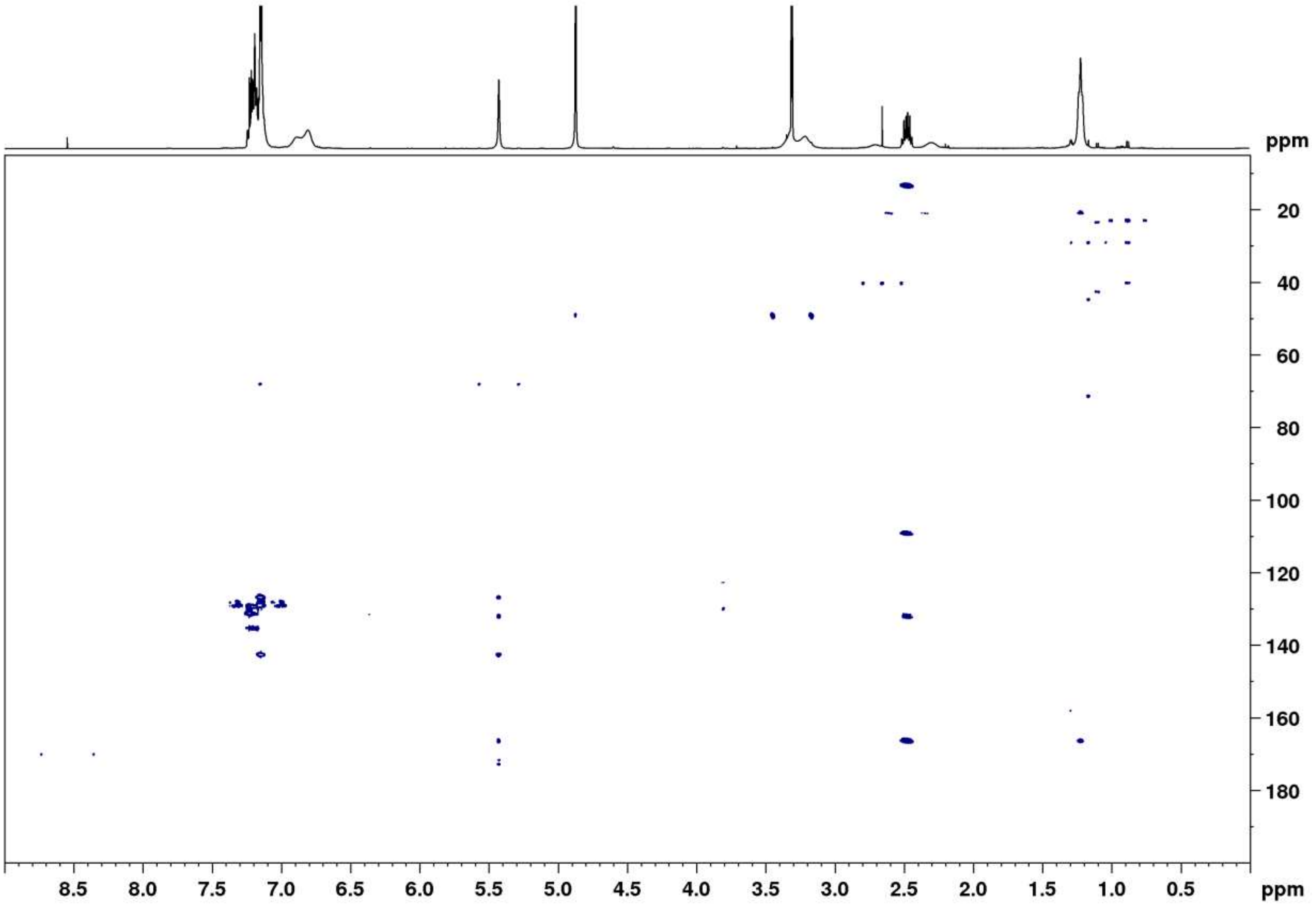


**Figure S57.** HMBC spectrum of **8** (500 MHz, $CD_3OD$).

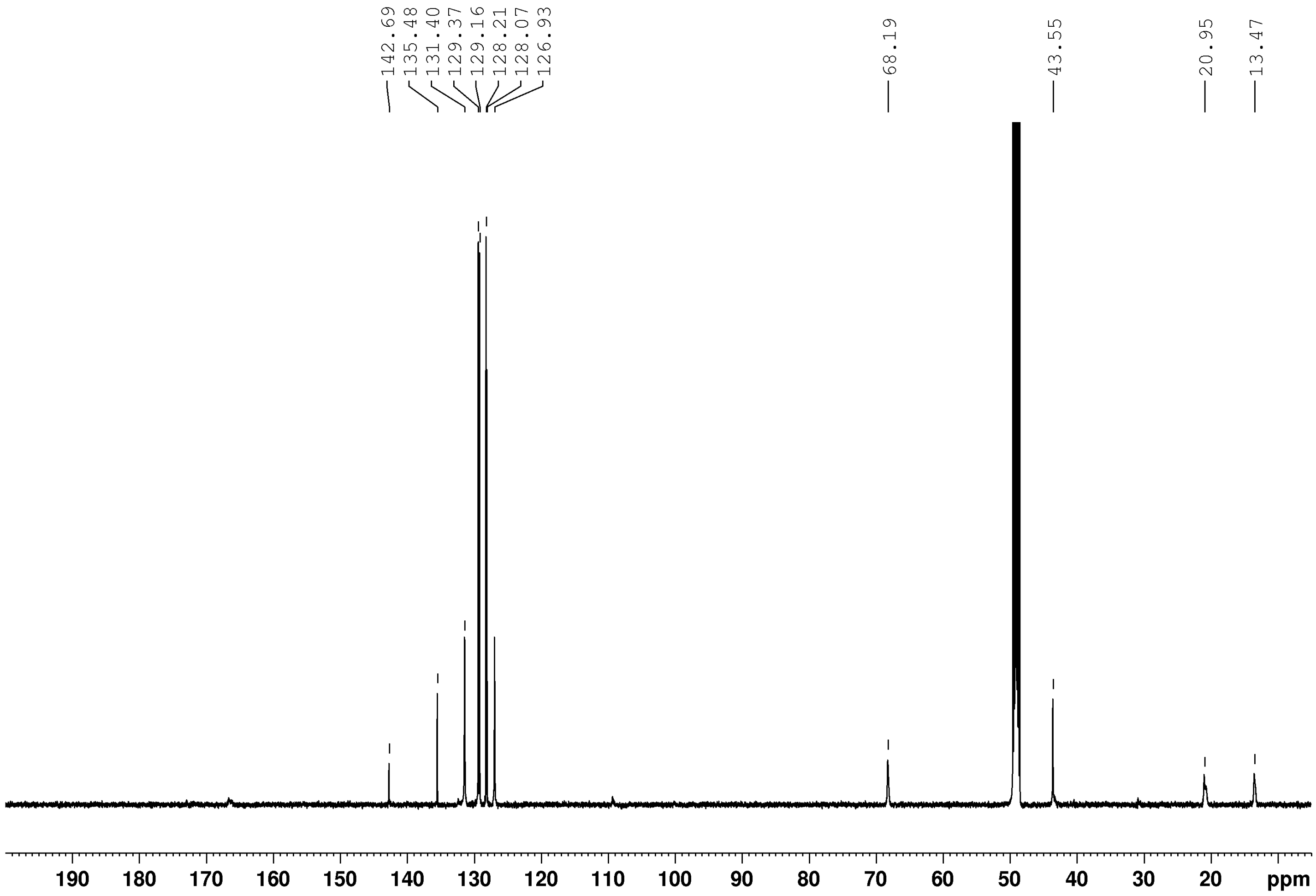


**Figure S58.** $^{13}C$ NMR spectrum of **8** (125 MHz, $CD_3OD$).

# 5. References


1 Dong, C., Flecks, S., Unversucht, S., Haupt, C., van Pée, K.-H., Naismith, J.H. (2005) Tryptophan 7-halogenase (PrnA) structure suggests a mechanism for regioselective chlorination. *Science (New York, N.Y.)*, **309** (5744), 2216–2219.

2 Henikoff, S. and Henikoff, J.G. (1992) Amino acid substitution matrices from protein blocks. *PNAS*, **89** (22), 10915–10919.

3 D'Agostino, P.M., Seel, C.J., Gulder, T., Gulder, T. (2021) *(Bio-)Synthesis of the Aquatic Phytotoxin Cyanobacterin – A Paradigm for Furanolide Core Structure Assembly*.

4 Neumann, P., Weidner, A., Pech, A., Stubbs, M.T., Tittmann, K. (2008) Structural basis for membrane binding and catalytic activation of the peripheral membrane enzyme pyruvate oxidase from Escherichia coli. *PNAS*, **105** (45), 17390–17395.

5 Lee, W.C., Cheon, D., Kim, Y. (2019) *Crystal structure of KAS III from Propionibacterium acnes*.

6 Hou, J., Chruszcz, M., Zheng, H., Cooper, D.R., Chordia, M.D., Zimmerman, M.D., Anderson, W.F., Minor, W. (2014) *Beta-ketoacyl-(acyl carrier protein) synthase III-2 (FabH2) from Vibrio cholerae soaked with Acetyl-CoA*.
